# Responsive ROS‐Augmented Prodrug Hybridization Nanoassemblies for Multidimensionally Synergitic Treatment of Hepatocellular Carcinoma in Cascade Assaults

**DOI:** 10.1002/advs.202501420

**Published:** 2025-05-05

**Authors:** Yingjie Zeng, Yuening Cao, Senmiao Ren, Chaozheng Zhang, Jianan Liu, Ke Liu, Yan Wang, Hongyu Chen, Fengjiao Zhou, Xiuli Yang, Xian Ge, Tingting Zhang, Tianbao Wang, Yao He, Defang Li, Chuantao Zhang, Jun Lu

**Affiliations:** ^1^ State Key Laboratory of Southwestern Chinese Medicine Resources School of Pharmacy Chengdu University of Traditional Chinese Medicine Chengdu 611137 China; ^2^ Department of Respiratory Medicine Hospital of Chengdu University of Traditional Chinese Medicine Chengdu 610075 China; ^3^ College of Pharmacy Hebei University of Chinese Medicine Shijiazhuang 050200 China; ^4^ Sichuan Clinical Research Center for Cancer Sichuan Cancer Hospital & Institute Sichuan Cancer Center Affiliated Cancer Hospital of University of Electronic Science and Technology of China Chengdu 610041 China

**Keywords:** cascade assaults, cuproptosis, hepatocellular carcinoma, oxidative stress, prodrug hybridization nanoassembly

## Abstract

The rapid deterioration and progression of hepatocellular carcinoma (HCC) is intimately associated with copper ion overload, and integrating the cuproptosis mechanism for the treatment of HCC presents a promising prospect. Nevertheless, cell death complexity renders efficient removal of all HCC cells insufficient solely relying on the cuproptosis pathway. Herein, the GSH‐responsive prodrug hybridization nanoassembly CA‐4S_2_@ES‐Cu is exploited, which targets the delivery of copper ions to mitochondria via Elesclomol, contributing to mitochondrial dysfunction and evoking cuproptosis. Simultaneously, CA‐4S_2_ depletes GSH to release CA‐4, disrupting microtubule function and suppressing HCC cell proliferation and angiogenesis, to realize a dual attack against copper ion‐mediated deterioration and metastasis of HCC. Furthermore, both in the HCC mouse model synergistically elicit oxidative stress to amplify the cuproptosis effect and release activated immunogenetic cell death to initiate a vigorous antitumor immune response in cascade assault modality. Conclusively, the multilevel synergistic assault penetrates the limitations of single therapy and implements a multidimensional targeted treatment for HCC.

## Graphical abstract

This study proposes a novel HCC therapy using CA‐4S2@ES‐Cu nanomedicines to amplify cuproptosis, enhancing chemotherapy and immunotherapy. CA‐4 and ES‐Cu synergistically inhibit tumor growth, induce immune responses, and reverse the immunosuppressive microenvironment, offering an effective multidimensional therapeutic strategy for HCC treatment.

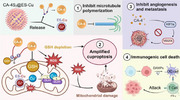

## 1 Introduction

Copper homeostatic imbalance is intimately associated with tumorigenesis and progression.^[^ ^1^ ^]^ With a pivotal function in copper metabolism, the liver is extremely susceptible to copper homeostatic imbalances owing to its synthesis and secretion of copper‐binding proteins as well as its regulation of copper bioprocesses.^[^ ^2^ ^]^ Clinical studies have indicated that copper ion levels in hepatocellular carcinoma (HCC) patients are substantially elevated compared to those in healthy individuals, and copper ion concentrations are negatively correlated with the pathological stage and prognosis of the tumors.^[^ ^3^, ^4^ ^]^ Besides, copper ions notably augmented the metastatic and invasive ability of HCC by inducing genomic instability, promoting angiogenesis, and initiating pro‐tumorigenic signaling pathways.^[^ ^5^, ^6^, ^7^, ^8^ ^]^ In view of the massive copper accumulation in the liver, triggering cell‐specific cuproptosis mechanisms by harnessing copper ions has emerged as a prospective research field for HCC treatment in recent years.

Elesclomol (ES), a unique copper ion carrier, is highly selective for mitochondria and is capable of transporting Cu^2+^ into mitochondria by forming complexes with Cu^2+^ at a ratio of 1:1.^[^ ^9^ ^]^ After the reduction of Cu^2+^ to Cu^+^ by ferredoxin 1 (FDX1), the ES excreted from the cytosol continuously transports Cu^2+^ to the mitochondria. Reactive oxygen species (ROS) generation by ES via Cu‐Fenton reaction could lead to mitochondrial damage and apoptosis, thus exerting anticancer effects, as well as triggering oxidative stress, thereby contributing to immunogenic cell death (ICD). Studies have revealed that GSH possesses a powerful antioxidant capacity to scavenge intracellular cuprous ions and ROS, thereby protecting organisms from damage due to oxidative stress.^[^ ^10^, ^11^ ^]^ In addition, the robust self‐regulation of redox homeostasis by tumor cells diminished the antitumor effect from ES alone.^[^ ^12^ ^]^ Hence, disrupting redox homeostasis could be a valuable strategy to improve ES antitumor effects.

Combretastatin A‐4 (CA‐4), a classical inhibitor of microtubule polymerization, possesses a potent tumor vascular disrupting property. Given the rich vascularity of HCC, CA‐4 exhibits strong anti‐HCC potential. Nevertheless, CA‐4 has been demonstrated to render chemotherapy unsuccessful because of poor lethality of residual tumor tissue, despite its capability to cause massive tumor cell necrosis. Besides, the extremely poor water solubility results in lower oral bioavailability, which also attenuates its antitumor activity and limits its clinical application.^[^ ^13^, ^14^ ^]^

Currently, the combined application of chemotherapy and cuproptosis has demonstrated excellent efficacy in antitumor therapy depending on its unique synergistic effect. Chemotherapy potentiates the intensified attack on tumor cells, while cuproptosis mitigates drug tolerance and toxic damage to normal tissues by inducing specific cell death pathways. Moreover, the combined effect of chemotherapy and cuproptosis contributes to the breakthrough of the tumor microenvironment‐mediated immune escape mechanism. Chemotherapy not only stimulates the immune response by directly destroying tumor cells, but also alters the tumor microenvironment and enhances the recognition and attack function of immune cells; whereas the metabolic disorders and cellular stress signals induced by cuproptosis provide immunostimulatory effects, which are able to activate the immune response while further increasing the sensitivity of tumor cells to chemotherapy.^[^ ^15^, ^16^, ^17^ ^]^ Consequently, selected appropriate chemotherapeutic methods in combination with cuproptosis could not only eliminate tumor cells directly, but also strengthen the antitumor effect of the immune system by inducing ICD, activating dendritic cells and T cells, thus providing a more favorable immunotherapy environment and generating a durable antitumor immune response.

**Scheme 1:**
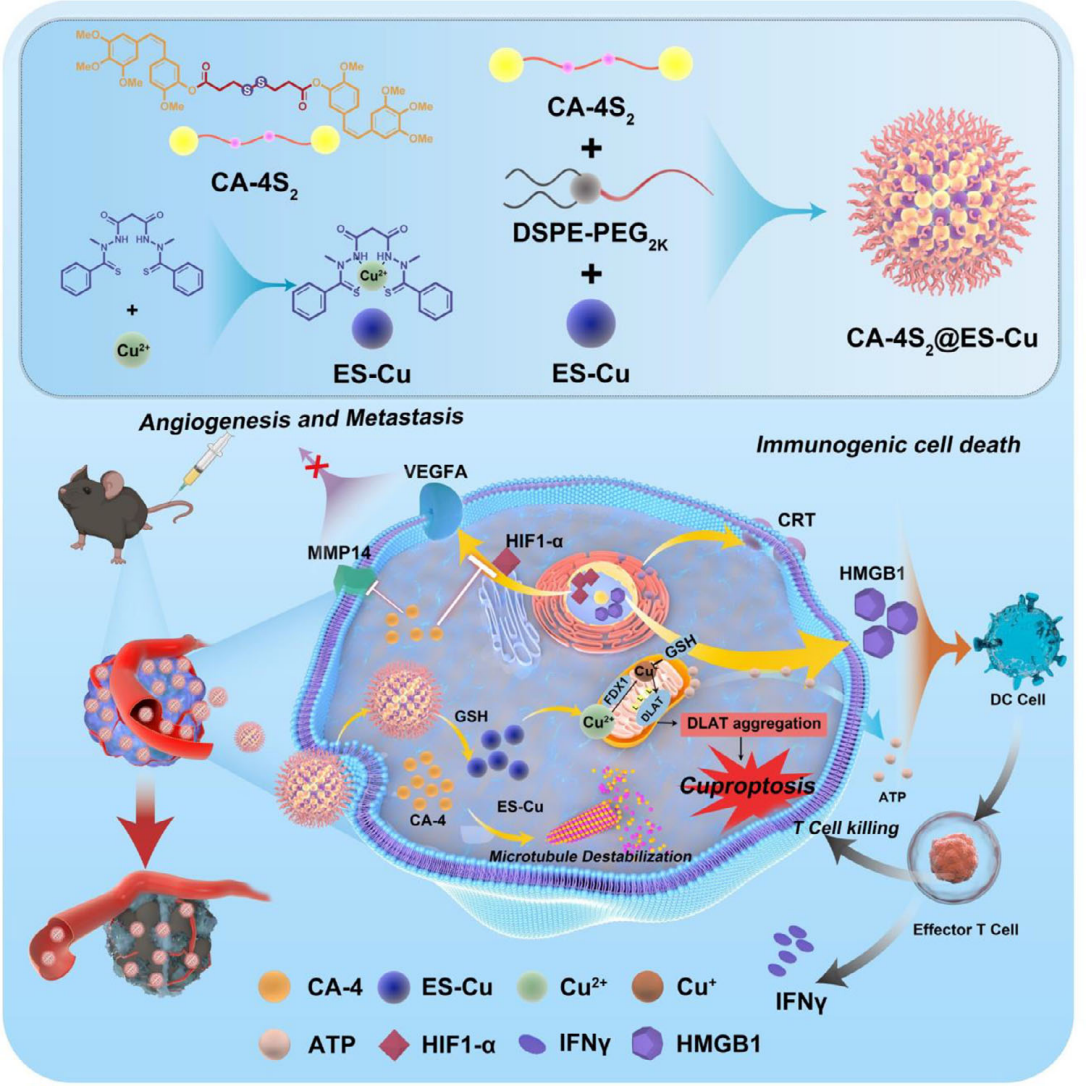
Mechanistic illustration of the CA‐4S_2_@ES‐Cu for synergistic cascade therapy against HCC.

The prodrug hybridization nanoassemblies integrate prodrugs with nanotechnology and offer efficient drug loading capacity, high‐delivery efficiency, superior biocompatibility, and controllable drug release characteristics. By simultaneously delivering multiple small‐molecule prodrugs to the tumor site and releasing the primary drug at a defined point in time, this system not only achieves synergistic treatment and improves efficacy, but also dramatically reduces organ toxicity, showcasing enormous potential for cancer treatment.^[^ ^18^, ^19^ ^]^ To surmount the respective limitations of CA‐4 and ES‐Cu in liver cancer treatment, CA‐4S_2_@ES‐Cu nanomedicine was designed and prepared in this study (**Scheme** **1**). Under the tumor microenvironment stimulation, CA‐4S_2_@ES‐Cu could release CA‐4 and ES‐Cu simultaneously. Subsequently, CA‐4 repressed cellular microtubule polymerization, blocked tumor cell mitosis and induced ROS generation; ES‐Cu was directly targeted and transported to mitochondria, triggering dihydrolipoamide S‐acetyltransferase (DLAT) oligomerization and mitochondrial metabolism disruption with massive ROS accumulation. Furthermore, disulfide bond breakage depleted intracellular GSH, further impairing cellular antioxidant capacity and dramatically intensifying cuproptosis and intracellular oxidative stress. CA‐4S_2_@ES‐Cu potentially possessed the capability to target copper ion depletion and regulate copper metabolism, simultaneously, restrained the angiogenic pathway to effectively cut off the blood supply to the tumor and interrupted the progression and metastasis of HCC triggered by copper ion superload. Surprisingly, CA‐4S_2_@ES‐Cu could stimulate the release of abundant damage associated molecular patterns (DAMPs), efficiently modulate the tumor microenvironment and enormously potentiate the immune cell infiltration, thereby eliciting an immune response. Therefore, CA‐4S_2_@ES‐Cu is a promising nanomedicine for the effective treatment of HCC through a multidimensional manner.

## 2 Results and Discussion

### 2.1 Cu^2^⁺ Promoted HCC Progression and Deterioration

**Figure 1:**
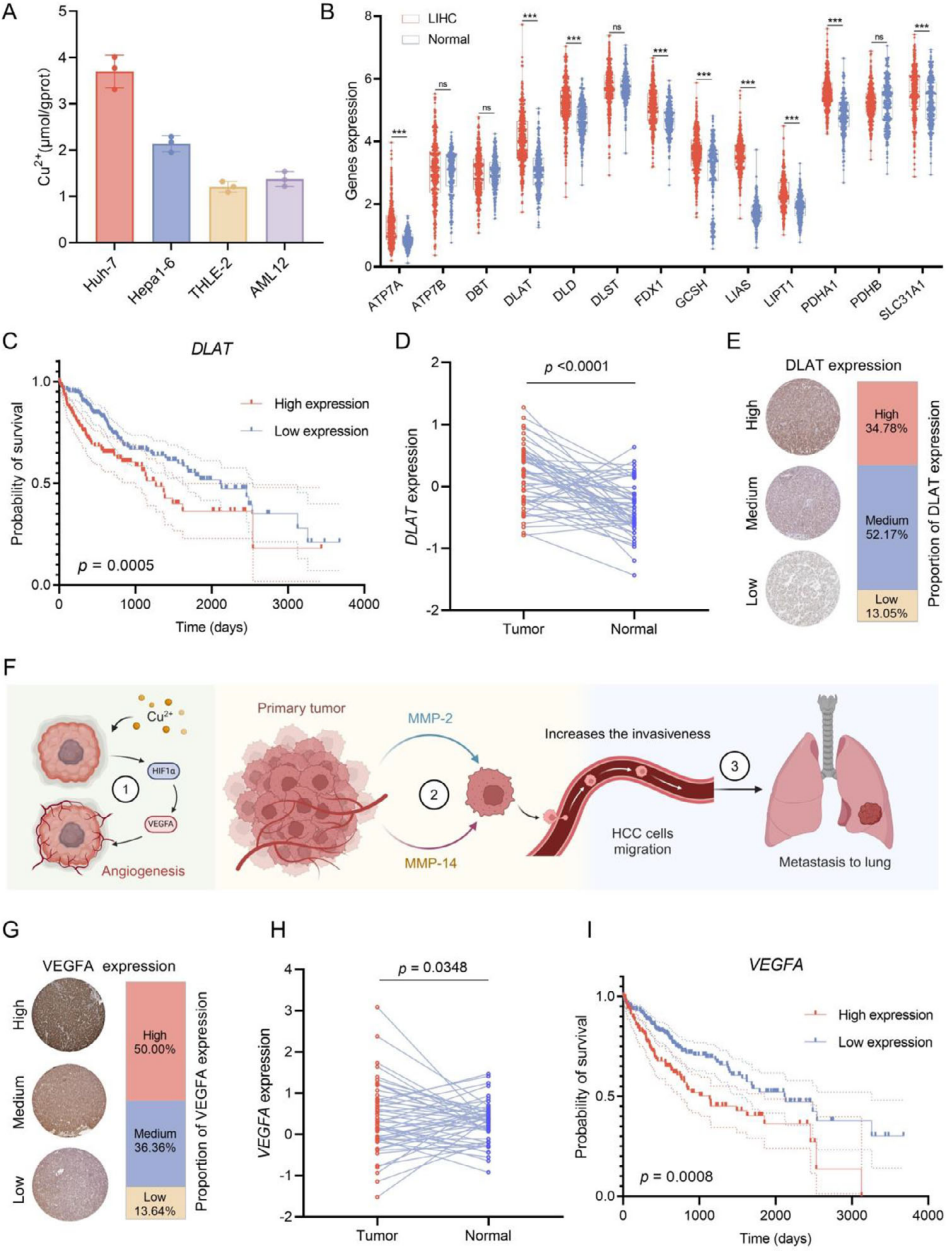
Cu^2^⁺ promoted HCC progression and deterioration. A) Copper ion levels in various HCC cell lines and normal liver cells. B) Expression levels of copper death‐related genes in HCC tissues and normal tissues. C) Survival analysis in HCC patients with *DLAT*. D) Differential expression of *DLAT* in paired HCC tissues and paracancerous tissues. E) DLAT expression levels in HCC tissues. F) Schematic diagram of copper ion promoting metastasis of HCC. G) VEGFA expression levels in HCC tissues. H) Differential expression of *VEGFA* in paired HCC tissues and paracancerous tissues. I) Survival analysis in HCC patients with *VEGFA*. *** *p* < 0.001, versus the control group.

Hepatology is the primary organ for copper metabolism, and an imbalance in copper homeostasis would cause serious hepatic diseases.^[^ ^20^ ^]^ Concurrently, copper ions are essential for the maintenance of biological functions, and slight elevations in copper ion levels promote tumorigenesis and progression.^[^ ^21^, ^22^ ^]^ Compared to normal hepatocytes, HCC cells harbored substantially higher levels of basal copper ions (**Figure** **1**A). Simultaneously, the analysis of 13 copper ion‐related genes among a dataset of 371 HCC tissues and 276 normal tissues derived from the TCGA and GETx databases revealed that nine related genes were aberrantly expressed in HCC tissues compared with normal tissues (Figure 1B).^[^ ^23^, ^24^ ^]^ Subsequently, prognostic analysis of the differential genes indicated that only *ATP7A* and *DLAT* were prognostic (*p*<0.05), with *DLAT* being the significance. The expression level of *DLAT* was associated with a poorer prognosis for HCC patients (Figure 1C; Figure S1, Supporting Information). Additionally, in terms of paired tumors and paracancerous tissues in TCGA, *DLAT* presented an elevated expression in the tested cancers (Figure 1D). Similarly, DLAT was overexpressed in HCC tissues (Figure 1E). Copper ions facilitated the tumor vascular endothelial cell proliferation and migration by activating hypoxia‐inducible factor α (HIF‐1α) and vascular endothelial growth factor (VEGFA), and promoted HCC metastasis through the up‐regulation of matrix metalloproteinase 2 (MMP‐2) and matrix metalloproteinase 14 (MMP‐14) (Figure 1F). VEGFA, a critical factor modulating angiogenesis and promoting tumor metastasis, is highly expressed in HCC tissues (Figure 1G). For the paired tumors and paraneoplastic tissues in TCGA, *VEGFA* was observed to be expressed at high levels in HCC (Figure 1H). Tragically, VEGFA was as well prognostic with high expression being unfavorable in HCC (Figure 1I). Furthermore, copper ions treatment in vitro alone dramatically up‐regulated VEGFA expression in HCC cells in assays, whereas the HIF‐1α inhibitor (PX‐478) administration group significantly suppressed VEGFA levels (Figure S2, Supporting Information). Upon co‐administration of Cu^2^⁺ with PX‐478, Cu^2^⁺‐induced VEGFA overexpression was notably restrained, and the copper chelator (DDTC) displayed a comparable inhibitory effect, signifying that copper accumulation might lead to VEGF overexpression in association with the HIF‐1α activity. In summary, copper ions, by regulating proteins such as DLAT and VEGFA and their associated pathways, promoted the development, angiogenesis, and metastasis of HCC, and were closely associated with poor prognosis in patients. Therefore, targeting copper metabolism might have been a potential effective therapeutic strategy for HCC.

### 2.2 Synthesis and Validation of CA‐4 Dimer Prodrugs

**Figure 2:**
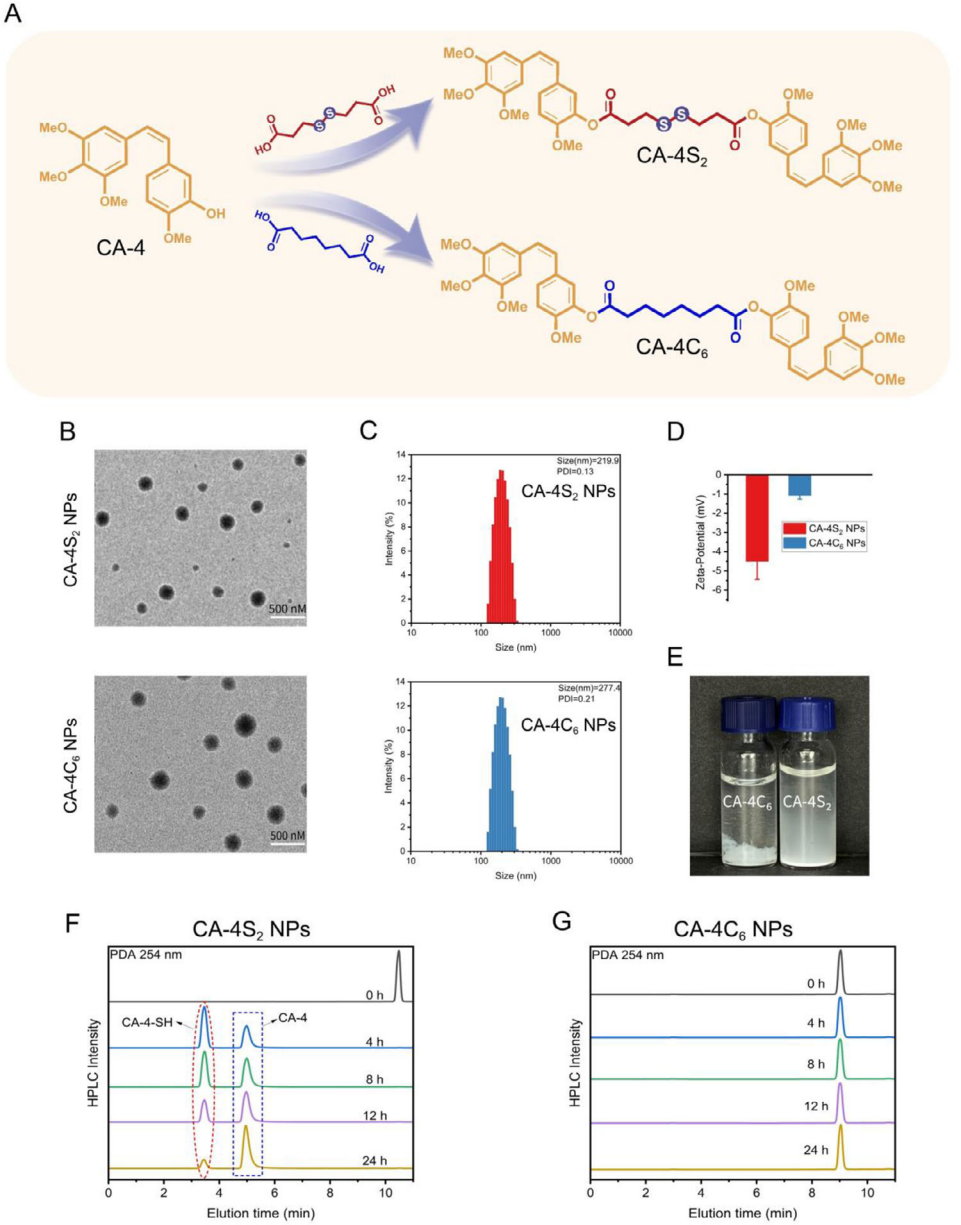
Synthesis and self‐assembly ability of prodrugs. A) Synthesis of CA‐4 dimer prodrugs. B) Representative TEM images of CA‐4S_2_ NPs and CA‐4C_6_ NPs. C,D) The hydrodynamic size distribution and zeta potentials of CA‐4S_2_ NPs and CA‐4C_6_ NPs (in ultrapure water) (n = 3). E) Appearance of prodrug nanoassemblies (0.3 mg mL^−1^) stored at room temperature for 8 h. F) GSH‐responsive mechanism of CA‐4S_2_ NPs in 10 mm GSH. G) GSH‐responsive mechanism of CA‐4C_6_ NPs in 10 mm GSH.

CA‐4, a biphenylstilbene‐based microtubule protein inhibitor, is limited in its clinical application due to its structural instability and poor water solubility.^[^ ^25^ ^]^ Therefore, two CA‐4 dimers were designed and synthesized employing disulfide bonds and aliphatic carbon chains as connecting linkages, with the expectation of obtaining CA‐4 prodrugs with favorable stability, strong activity, and the ability to self‐assemble into nanoforms (**Figure** **2**A; Schemes S1–S4, Supporting Information). By transmission electron microscopy (TEM), the two dimer prodrugs were capable of forming nanospherical structures, CA‐4S_2_ NPs and CA‐4C_6_ NPs, respectively, with more regular nanomorphology of the formed CA‐4S_2_ NPs (Figure 2B). Additionally, the average particle sizes of CA‐4S_2_ NPs and CA‐4C_6_ NPs were 219.9 nm and 277.4 nm, respectively, determined by dynamic light scattering (DLS) (Figure 2C). Meanwhile, both nanoparticles presented zeta potentials of – 4.50 and – 1.07 mV in pure water, respectively (Figure 2D). Nevertheless, with increasing time, flocculent generation was observed in solution for both self‐assembled nanodrugs, except for CA‐4C_6_ NPs forming a large number of aggregated precipitates within 12 h (Figure 2E). In comparison with CA‐4C_6_, CA‐4S_2_ demonstrated a superior self‐assembly capability, presumably due to the diagonal angle of the disulfide bond that improved the spatial softness, and thus facilitated the self‐assembly and enhanced the nano‐stability.^[^ ^22^, ^26^ ^]^ Generally, the connecting bond controls drug release, and disulfide bonds are conventionally responsive to GSH in the tumor microenvironment. By combining HPLC and LC‐MS, CA‐4S_2_ NPs were gradually cleaved to the intermediates CA‐4‐SH and CA‐4 at a concentration of 10 mm GSH, but remained relatively stable in the absence of GSH (Figure 2F; Figures S3A,C, S4A, Supporting Information). In contrast, CA‐4C_6_ showed little cleavage under both concentrations of GSH (Figure 2G; Figures S3B, S4B, Supporting Information). Differences in GSH responsiveness between CA‐4S_2_ NPs and CA‐4C_6_ NPs indicated that CA‐4S_2_ NPs were potentially responsive to the tumor microenvironment.

### 2.3 Preparation and Characterization of CA‐4S_2_@ES‐Cu and CA‐4C_6_@ES‐Cu

**Figure 3:**
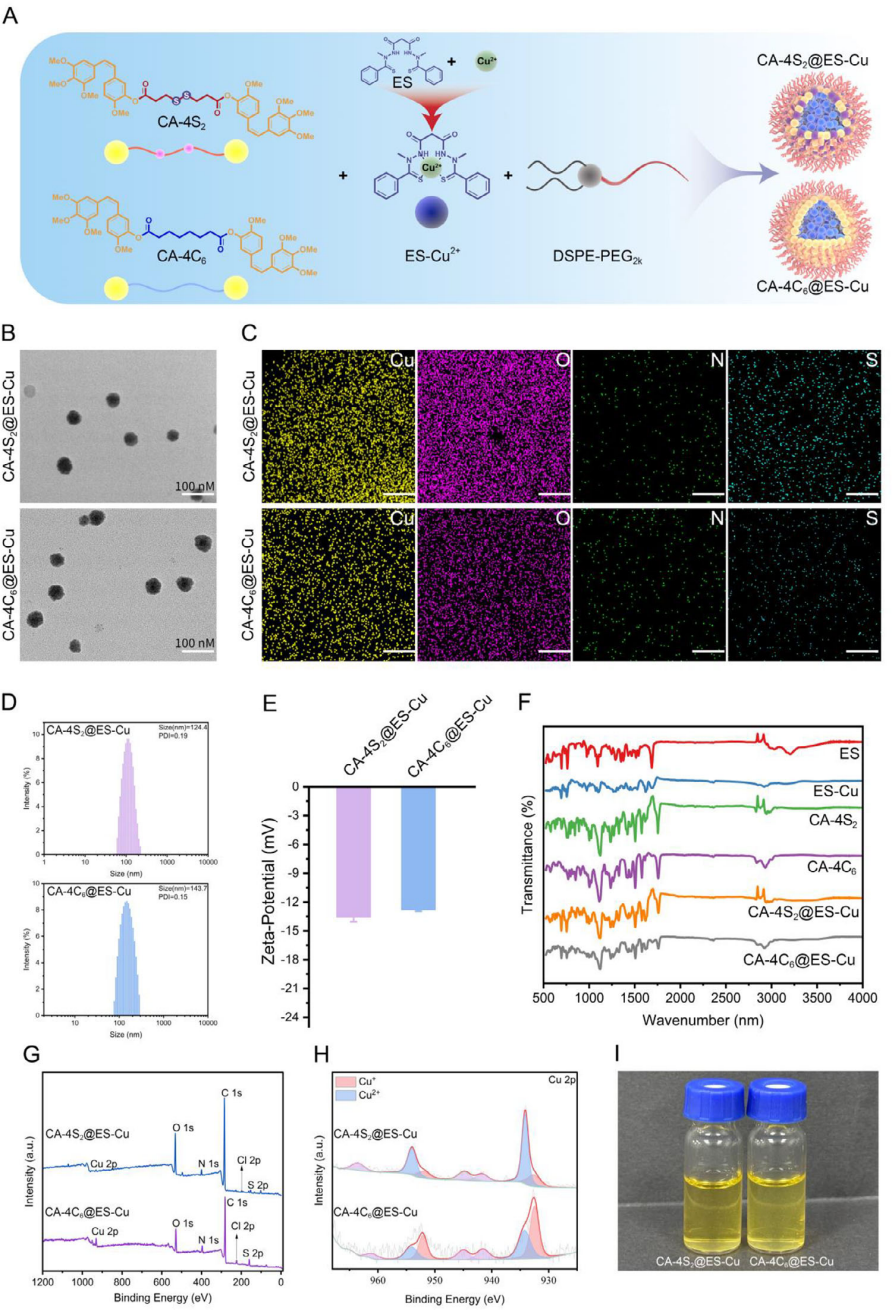
Preparation and characterization of CA‐4S_2_@ES‐Cu and CA‐4C_6_@ES‐Cu. A) Schematic diagram of the preparation for CA‐4S_2_@ES‐Cu and CA‐4C_6_@ES‐Cu. B) Representative TEM images of CA‐4S_2_@ES‐Cu and CA‐4C_6_@ES‐Cu. C) Representative element maps (Cu, O, N, and S) of CA‐4S_2_@ES‐Cu and CA‐4C_6_@ES‐Cu. Scale bar 200 nm. ES formula: C_19_H_20_N_4_O_2_S_2_, CA‐4S_2_ formula: C_42_H_46_O_12_S_2_, CA‐4C_6_ formula: C_44_H_50_O_12_. D,E) The hydrodynamic size distribution and zeta potentials of CA‐4S_2_@ES‐Cu and CA‐4C_6_@ES‐Cu (in ultrapure water) (n = 3). F) FTIR spectra of ES, ES‐Cu, CA‐4S_2_, CA‐4C_6_, CA‐4S_2_@ES‐Cu and CA‐4C_6_@ES‐Cu. G) XPS analysis of CA‐4S_2_@ES‐Cu and CA‐4C_6_@ES‐Cu. H) High‐resolution N 1s XPS spectra of CA‐4S_2_@ES‐Cu and CA‐4C_6_@ES‐Cu. I) The appearance of CA‐4S_2_@ES‐Cu and CA‐4C_6_@ES‐Cu (0.5 mg mL^−1^) stored at 4 °C for 15 d.

Two nanodrugs, CA‐4S_2_@ES‐Cu and CA‐4C_6_@ES‐Cu in which ES‐Cu is a 1:1 complex of elesclomol (ES) and Cu^2+^, were successfully prepared by the nanoprecipitation approach after groping the conditions (**Figure** **3**A; Figure S5, Supporting Information). The encapsulation efficiency (EE) of ES and CA‐4S_2_ for CA‐4S_2_@ES‐Cu NPs was calculated to be 75% and 77%, with a drug loading capacity (DLC) of 16.04% and 34.63%, respectively, and the DLC of Cu^2+^ was 2.9%; while for CA‐4C_6_@ES‐Cu NPs, the EE of ES and CA‐4C_6_ was 62% and 67%, with a DLC of 7.13% and 14.98%, respectively, and the DLC of Cu^2+^ was 1.3%. Subsequently, CA‐4S_2_@ES‐Cu and CA‐4C_6_@ES‐Cu presented a homogeneous spherical structure as depicted by TEM (Figure 3B). Moreover, the elemental spectra consisting of Cu, O, N, and S elements further supported the successful encapsulation of ES‐Cu and CA‐4S_2_, as well as ES‐Cu and CA‐4C_6_ in CA‐4S_2_@ES‐Cu and CA‐4C_6_@ES‐Cu, respectively (Figure 3C). The average particle sizes of CA‐4S_2_@ES‐Cu and CA‐4C_6_@ES‐Cu were determined by DLS to be 124.4 and 143.7 nm, respectively, and in ultrapure water their zeta potentials were −13.6 and −12.8 mV, respectively (Figure 3D,E). The UV–vis spectra revealed that the absorption of CA‐4S_2_@ES‐Cu and CA‐4C_6_@ES‐Cu was intensified at 420 nm, giving the characteristic absorption peaks of ES‐Cu (Figure S6, Supporting Information). Furthermore, the characteristic peaks of ES‐Cu, CA‐S_2_, CA‐4C_6_ were identified in the FTIR spectra of CA‐4S_2_@ES‐Cu and CA‐4C_6_@ES‐Cu by inter‐component comparisons (Figure 3F). Besides, X‐ray photoelectron spectroscopy (XPS) further confirmed that the two prepared nanoparticles incorporated Cu, O, N and S elements (Figure 3G,H; Figure S7, Supporting Information).

Stable nanomedicines are effective in avoiding early degradation or clearance in the body, thus assuring drug accumulation and maximum efficacy at the tumor site. The respective dispersions of CA‐4S_2_@ES‐Cu and CA‐4C_6_@ES‐Cu were able to maintain stability for up to 15 days in PBS (pH 7.4) at 4 °C and maintained a favorable nanomorphology (Figure 3I; Figures S8A, S9A, Supporting Information). In addition, the average particle sizes of CA‐4S_2_@ES‐Cu and CA‐4C_6_@ES‐Cu in PBS (pH 7.4) solution containing 10% FBS as well as PBS (pH 7.4) solution containing 10% rat plasma/heparin increased only slightly due to protein adsorption at the initiation stage, and there was no noticeable variation in either case over 72 h (Figure S9B,C, Supporting Information). After 48 h in the absence of GSH, the release of ES from CA‐4S_2_@ES‐Cu still remained no more than 10% and CA‐4 no more than 5%. However, at higher GSH concentrations (1, 5, and 10 mm), CA‐4 and ES were progressively released from CA‐4S_2_@ES‐Cu over time and more than 70% of CA‐4 and ES were released within 24 h (Figure S10A,B, Supporting Information). Precisely, such sensitivity could ensure that CA‐4S_2_@ES‐Cu maintained a relatively stable state in the blood stream, while facilitating the release of the active drugs in GSH‐rich tumor tissues. In comparison, CA‐4C_6_@ES‐Cu released little CA‐4 and less than 10% of ES under the three GSH concentration conditions (Figure S10C,D, Supporting Information). Taken together, the capability of CA‐4S_2_@ES‐Cu to achieve effective drug release in the tumor microenvironment fully accentuates the essential role of disulfide bonds in tumor‐targeted drug delivery. ^1^H NMR, ^13^C NMR and HPLC analysis of all synthesized compounds were provided in the supporting information (Figures S11–S16, Supporting Information).

### 2.4 Cellular uptake, Pharmacokinetics and In Vivo Distribution of CA‐4S_2_@ES‐Cu

**Figure 4:**
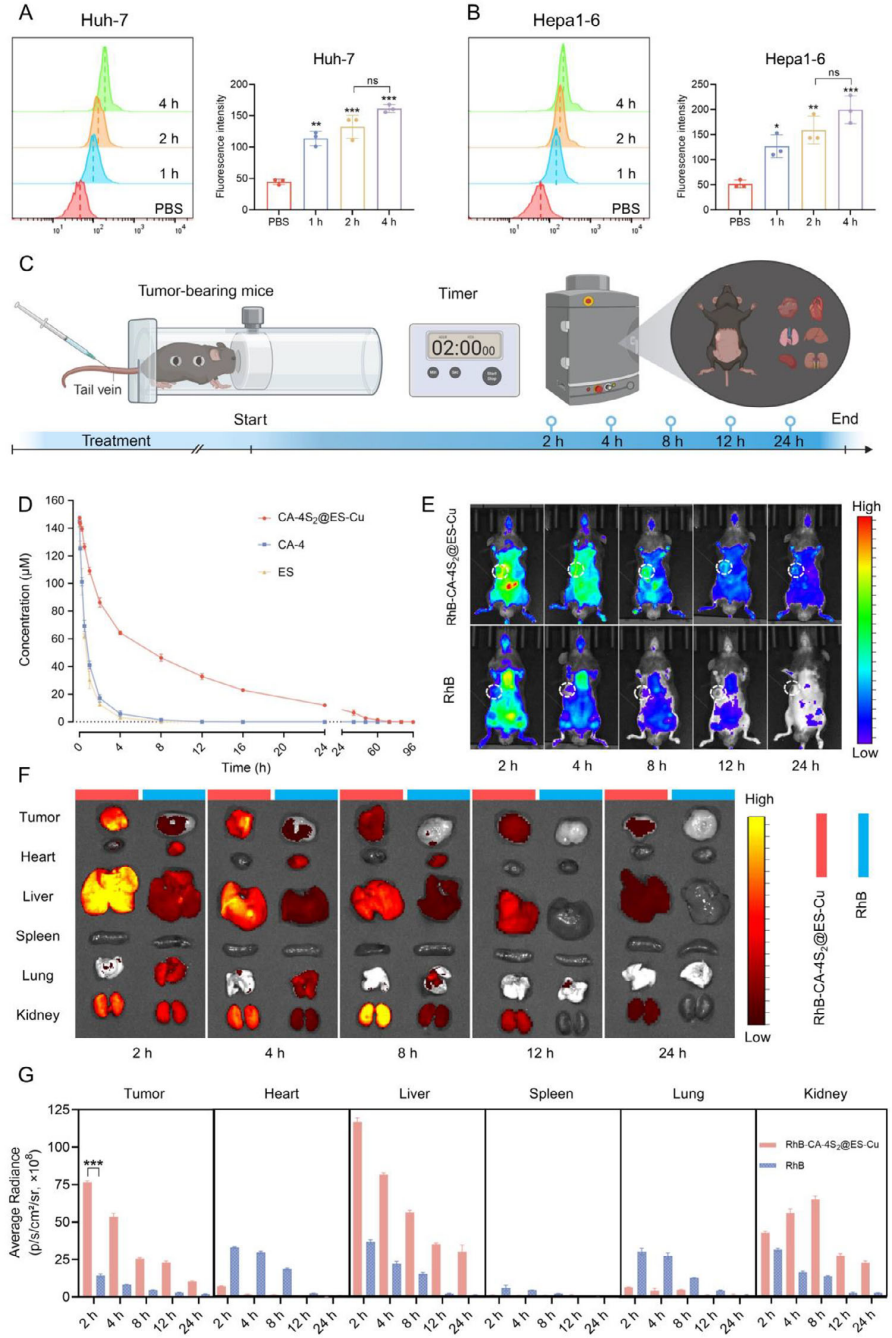
Cellular uptake, pharmacokinetics, and in vivo distribution of CA‐4S_2_@ES‐Cu. A,B) Uptake capacity of HCC cells for rhodamine B (RhB)‐labeled CA‐4S_2_@ES‐Cu. C) Schematic illustration of the experimental process for the biodistribution study in tumor‐bearing mice. D) Plasma concentration‐time curves of CA‐4, ES, and CA‐4S_2_@ES‐Cu. E) In vivo biodistribution of free RhB and RhB‐labeled CA‐4S_2_@ES‐Cu at indicated time points after intravenous injection. F) Ex vivo imaging of major organs and tumors collected at the indicated time points. G) Semi‐quantitative analysis of fluorescence intensity in ex vivo organs and tumors. *p < 0.05, **p < 0.01, ***p < 0.001, versus the specified group.

Given that the size of nanoparticles is a critical determinant of cellular uptake efficiency, flow cytometry analysis revealed that RhB‐labeled CA‐4S_2_@ES‐Cu exhibited time‐dependent uptake characteristics in HCC cells, with uptake efficiency significantly increasing over incubation time and reaching a plateau at ≈4 h (**Figure** **4**A,B). To further evaluate the in vivo performance of CA‐4S_2_@ES‐Cu as a drug delivery system, its pharmacokinetic profile and tissue distribution characteristics were systematically investigated (Figure 4C). The plasma half‐lives (t1/2) of CA‐4 and ES were 0.85 and 0.67 h, respectively, whereas CA‐4S_2_@ES‐Cu exhibited a dramatically prolonged half‐life of 9.14 h, indicating that CA‐4S_2_@ES‐Cu possessed markedly enhanced stability and extended blood circulation time (Figure 4D). Following intravenous injection of RhB‐labeled CA‐4S_2_@ES‐Cu into Hepa1‐6 tumor‐bearing mice, real‐time monitoring was performed using an IVIS imaging system. Within 2 h after administration, the tumor site presented a remarkable enrichment of fluorescent signals already, although certain degree of nonspecific distribution was observed in primary viscera (Figure 4E). Notably, free RhB was rapidly eliminated from the tumor site at 4 h after administration, whereas CA‐4S_2_@ES‐Cu maintained detectable tumor fluorescence intensity after 24 h administration (Figure 4F,G). which robustly demonstrated the superior tumor‐targeting capability and long retention property of CA‐4S_2_@ES‐Cu. These findings vigorously demonstrated that CA‐4S_2_@ES‐Cu possessed superior tumor‐targeting capability and long‐lasting maintenance ability, providing critical experimental evidence for their potential as an effective antitumor drug delivery system.

### 2.5 In Vitro Antitumor Potential of CA‐4S_2_@ES‐Cu

**Figure 5:**
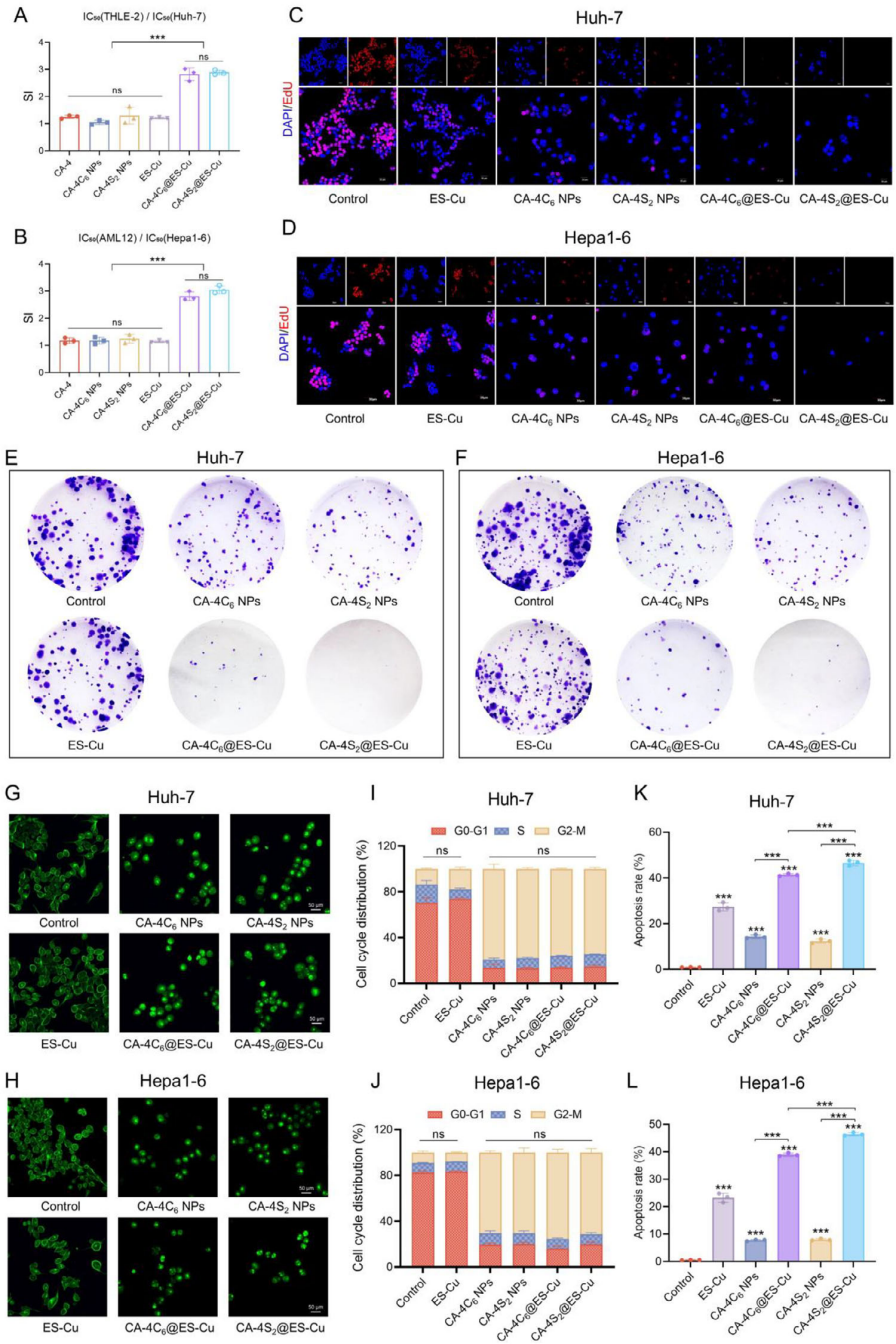
CA‐4S_2_@ES‐Cu exhibited anti‐HCC cell effects in vitro. A,B) Selectivity index (SI) of different drug groups. C,D) HCC cell proliferation after different drug treatments detected by EdU (×40). E,F) Clonogenic capacity of HCC cells after different treatments. G,H) Microtubule morphology changes in HCC cells after various drug treatments (×100). I,J) Cell cycle distribution of HCC cells after various drug treatments K, L) Quantitative analysis of apoptosis in HCC cells after different drug treatments. *p < 0.05, **p < 0.01, ***p < 0.001, versus the specified group.

Thereafter, in the assay of different cells treated with various drugs, CA‐4 was indicated to possess prominent toxic potency on HCC cells as well as on normal hepatocytes THLE‐2 and AML12 (Table S1, Supporting Information). For ES‐Cu, CA‐4, and their dimers, the selectivity indexes (SI = IC₅₀ (normal cells)/IC₅₀ (tumor cells)) ranged from 1 to 1.3 with no observable differences between the groups. In comparison, the nanodrugs CA‐4C_6_@ES‐Cu and CA‐4S_2_@ES‐Cu formed after DSPE‐PEG2k encapsulation exhibited dramatically improved selectivity indexes, with a value of more than 2.5, specifically, SI values of 2.89 ± 0.09 and 3.04 ± 0.14 for CA‐4S_2_@ES‐Cu, respectively, indicating a favorable safety profile (**Figure** **5**A,B). Simultaneously, the cells in the different administration groups underwent corresponding morphological changes, encompassing deformation, expansion, and contraction (Figure S17, Supporting Information). Moreover, the percentage of EdU‐positive cells was obviously minimized after CA‐4C_6_@ES‐Cu and CA‐4S_2_@ES‐Cu treatment of HCC cells for 24 h, while the cell nuclei were enlarged, demonstrating an extremely powerful cell proliferation inhibitory capability (Figure 5C,D). Furthermore, CA‐4S_2_@ES‐Cu was found to have the strongest ability to inhibit cell cloning based on the results of colony integration experiments (Figure 5E,F).

Microtubules depolymerize and reorganize to form spindles in the pre‐division phase of cell division and participate in cell mitosis, as a unique biological function enabling them to be pivotal anti‐tumor drug targets.^[^ ^27^ ^]^ CA‐4 is a potent microtubule polymerization inhibitor, and its prodrugs CA‐4C_6_ NPs, CA‐4S_2_ NPs, CA‐4C_6_@ES‐Cu and CA‐4S_2_@ES‐Cu were also proven to significantly repress microtubule polymerization in HCC cells (Figure 5G,H). As indicated by the flow cytometry analysis, all the above nanomedicines could arrest the HCC cell cycle in G2/M phase, consistent with the CA‐4 effect (Figure 5I,J; Figures S18, S19, Supporting Information), indicating that CA‐4 dimerization and encapsulation process did not influence the antitumor mechanism of CA‐4 itself.^[^ ^28^ ^]^ In addition, the various drug treatments were able to induce apoptosis in HCC cells as detected by Annexin V/PI double staining assay (Figure 5K,L). The pro‐apoptotic activities of CA‐4S_2_ and CA‐4C_6_ NPs were comparable, yet markedly inferior to that of ES‐Cu. After encapsulated with ES‐Cu, CA‐4S_2_@ES‐Cu evoked apoptosis dramatically greater than CA‐4C_6_@ES‐Cu, probably associated with its release mechanism (Figure S20, Supporting Information). Collectively, CA‐4S_2_@ES‐Cu demonstrated potent antitumor potential in suppressing HCC cell proliferation and inducing apoptosis.

### 2.6 CA‐4S_2_@ES‐Cu Aggravated Oxidative Stress and Evoked Cuproptosis

**Figure 6:**
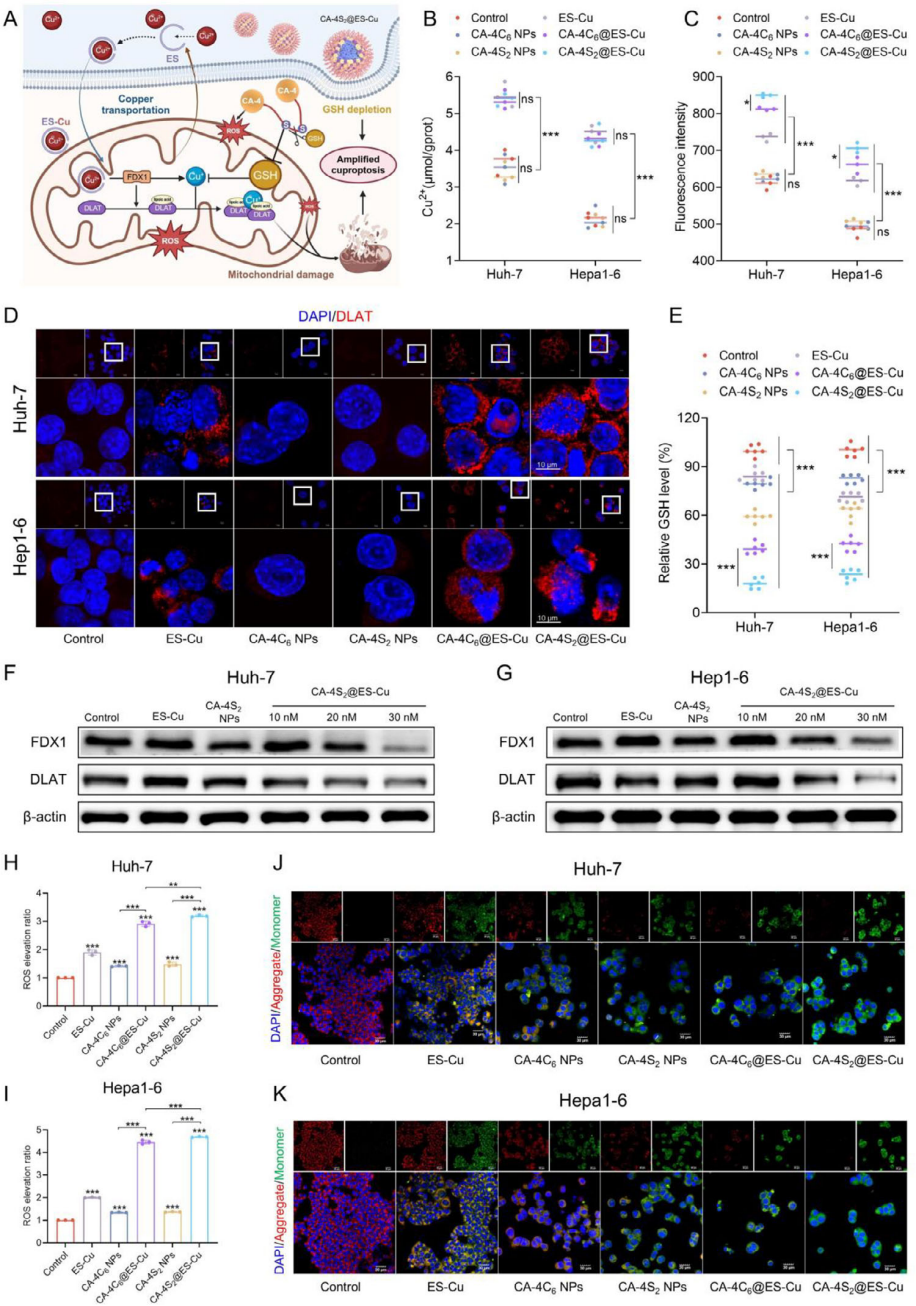
CA‐4S_2_@ES‐Cu induced cuproptosis in HCC cells. A) Schematic mechanism of CA‐4S_2_@ES‐Cu induced cuproptosis in HCC cells. B) Copper ion concentrations in HCC cells after various drug treatments. C) Cuprous ion levels in HCC cells after treatment with different drugs. D) DLAT oligomerization in HCC cells after various drug treatments in confocal microscopy images, scale bar: 10 µm. E) GSH levels after different drug treatments in HCC cells. F,G) Western blot analysis of variations in FDX1 and DLAT expression in HCC cells after different drug treatments. H,I) ROS levels in HCC cells after different drug treatments. J, K) Representative confocal microscopy images of mitochondrial membrane potential levels in HCC cells after different drug treatments, scale bar: 30 µm. *p < 0.05, **p < 0.01, ***p < 0.001, versus the specified group.

ES, as an efficient copper ion carrier, could form ES‐Cu complexes with extracellular Cu^2+^ in cancer cells, which then dissociate after cell‐directed transport to mitochondria, eventually triggering cellular cuproptosis by continuous copper ion accumulation in the mitochondria.^[^ ^9^ ^]^ In light thereof, we explored the contribution of CA‐4S_2_@ES‐Cu to the induced cuproptosis (**Figure** **6**A). Intracellular copper ion concentrations were regularly estimated to assess the efficiency of copper transport. The transport of copper ions by CA‐4C_6_ NPs and CA‐4S_2_ NPs was ineffective, however, the intracellular concentration of copper ions in HCC cells was dramatically elevated by about 1.5–2‐fold after treatment with ES‐Cu, CA‐4C_6_@ES‐Cu, and CA‐4S_2_@ES‐Cu (Figure 6B). Therefore, the absence of a meaningful difference between the three aforementioned dimers indicated that encapsulation of CA‐4 dimer with ES‐Cu had little impact on the copper‐transporting capability of ES. FDX1, a mitochondrial reductase involved in Fe–S cluster formation, could combine copper ions and reduce them to cuprous ions.^[^ ^29^ ^]^ In contrast to the copper ion content, both CA‐4C_6_@ES‐Cu and CA‐4S_2_@ES‐Cu exhibited superior cuprous ion production competence compared with ES‐Cu, and CA‐4S_2_@ES‐Cu, in particular, elevated intracellular cuprous ion levels in HCC cells by about 1.5‐fold (Figure 6C). In the process of cuproptosis, FDX1 proteins undertake the reduction of copper ions as well as the facilitation of multiple protein lipoylation modifications, a procedure that is dependent on lipoic acid in mitochondria. Cuprous ions elicit oligomerization of lipoic acid‐modified DLAT (A critical component in the pyruvate dehydrogenase complex), which impacts on the tricarboxylic acid cycle and triggers proteotoxic stress, consequently causing cuproptosis.^[^ ^30^ ^]^ Thereafter, with the visualization of DLAT oligomerization by immunofluorescence imaging, the findings were consistent with the cuprous ion generation results, and CA‐4C_6_@ES‐Cu and CA‐4S_2_@ES‐Cu revealed superior DLAT oligomerization than ES‐Cu, with CA‐4S_2_@ES‐Cu in particular showing the maximum degree of DLAT oligomerization in HCC cells (Figure 6D).

GSH, as a pivotal antioxidant in the body, not only maintains the redox homeostatic balance, but also prevents the combination of cuprous ions and lipoic acidified DLAT by means of its binding to cuprous ions, thereby decelerating the cuproptosis process.^[^ ^31^ ^]^ In HCC cells, ES‐Cu, CA‐4C_6_ NPs, and CA‐4S_2_ NPs all possessed a certain degree of GSH‐consuming ability, and among the three, CA‐4S_2_ NPs demonstrated outstanding GSH‐consuming ability, with the consumption of ≈70% GSH, which was correlated with its disulfide bonding capacity to react with GSH (Figure 6E). Nevertheless, CA‐4C_6_@ES‐Cu and CA‐4S_2_@ES‐Cu presented more pronounced GSH consumption phenotype, which might be associated with cuproptosis and the induced oxidative stress. Additionally, the expression of cuproptosis‐related proteins examined by Western blot revealed that CA‐4S_2_@ES‐Cu dramatically diminished the expression of FDX1 and soluble DLAT (Figure 6F,G). Cuproptosis was triggered by the aggregation of mitochondrial lipoylated proteins, after which the mitochondrial membrane suffered oxidative damage, leading to mitochondrial dysfunction.^[^ ^30^, ^32^ ^]^ As visualized by flow cytometry, all administration groups could efficiently induce ROS generation, with CA‐4S_2_@ES‐Cu being the most potent in triggering the ROS elevation (Figure S21, Supporting Information). In comparison with the control group, the intracellular levels of ROS were elevated about 3–4.5 times in the CA‐4S_2_@ES‐Cu treated cells (Figure 6H,I). Moreover, consistent with the level of mitochondrial membrane potential decrease, CA‐4S_2_@ES‐Cu evoked greater mitochondrial dysfunction (Figure 6J,K). In conclusion, CA‐4S_2_@ES‐Cu exacerbated oxidative stress via dual accumulation of ROS and amplified the cuproptosis effect by lowering GSH levels, resulting in a potent antitumor response.

### 2.7 CA‐4S_2_@ES‐Cu Stimulated ICD

**Figure 7:**
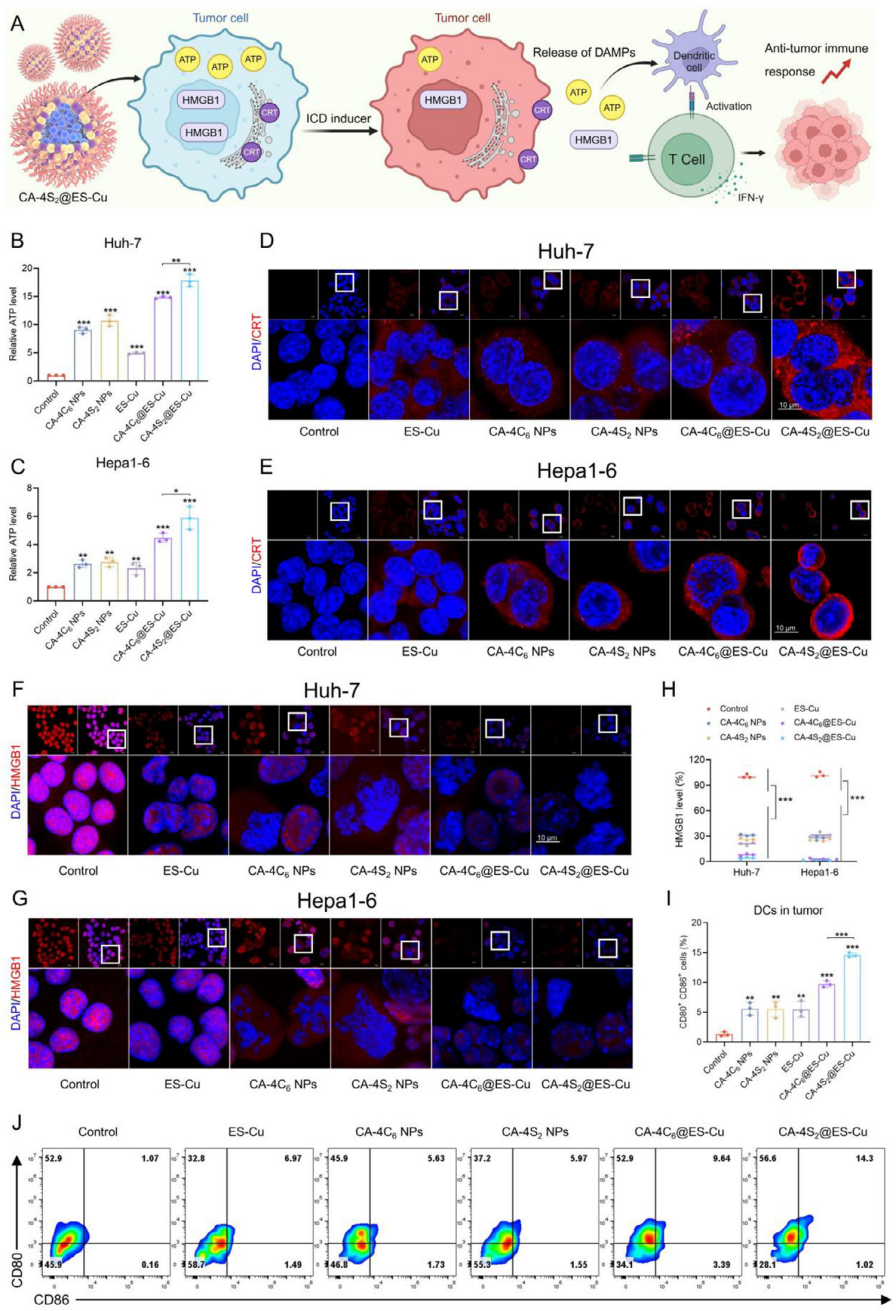
CA‐4S_2_@ES‐Cu stimulated ICD. A) Schematic diagram of the mechanism for stimulating ICD by CA‐4S_2_@ES‐Cu B,C) Release levels of ATP in HCC cells after treatment with various drugs. D,E) Representative confocal microscopy images of CRT exposure after various drug treatments of HCC cells, scale bar: 10 µm. F,G) Representative confocal microscopy images of HMGB1 release in HCC cells after various drug treatments, scale bar: 10 µm. H) Quantitative analysis of HMGB1 fluorescence intensity. I,J) Proportion of mature DCs measured by flow assay in HCC tissues after various drug treatments. *p < 0.05, **p < 0.01, ***p < 0.001, versus the specified group.

ICD is an innovative process to enhance tumor therapy by eliciting an adaptive immune response.^[^ ^33^ ^]^ Studies have demonstrated that cuproptosis could potentiate antitumor immune responses by stimulating ICD.^[^ ^34^, ^35^ ^]^ Besides, endoplasmic reticulum stress induced by high ROS concentration is a vital pathway to activate ICD in tumor cells.^[^ ^36^, ^37^ ^]^ Due to the intensive oxidative stress and cuproptosis induced by CA‐4S_2_@ES‐Cu, we evaluated its capacity to stimulate ICD (**Figure** **7**A).

An important feature of ICD is the release of DAMPs encompassing calreticulin (CRT) exposed on the cell surface, adenosine triphosphate (ATP) released into the extracellular space, and high‐mobility group box 1 (HMGB1).^[^ ^33^ ^]^ After CA‐4C_6_@ES‐Cu and CA‐4S_2_@ES‐Cu treatments, a large amount of ATP was released into the extracellular environment (Figure 7B,C). Moreover, the ATP release level induced by CA‐4S_2_@ES‐Cu was considerably higher than that induced by CA‐4C_6_@ES‐Cu, probably due to the severe mitochondrial damage evoked by CA‐4S_2_@ES‐Cu. Additionally, immunofluorescence results showed that CA‐4C_6_@ES‐Cu and CA‐4S_2_@ES‐Cu could dramatically promote large amounts of CRT exposure, releasing “eat me” signals. In contrast, the ability of ES‐Cu, CA‐4C_6_ NPs and CA‐4S_2_ NPs to induce CRT exposure was rather fragile (Figure 7D,E). Meanwhile, the fluorescence of HMGB1 in the nucleus of HCC cells presented a significant decrease after CA‐4C_6_@ES‐Cu and CA‐4S_2_@ES‐Cu treatments (Figure 7F–H). Unlike minimally induced CRT exposure, CA‐4C_6_ NPs and CA‐4S_2_ NPs were also efficient in inducing HMGB1 release, which might be associated with the microtubule inhibitory capacity of CA‐4. Among the key roles of ICD is the activation of dendritic cells (DCs), in which the release of DAMPs attracts DCs to migrate toward the tumor region and promotes their uptake and presentation of tumor antigens, thereby initiating a T‐cell‐mediated anti‐tumor immune response. As detected by flow cytometry, the percentage of mature DCs (CD80^+^ CD86^+^) in tumor tissues treated with CA‐4S_2_@ES‐Cu group was significantly higher than that in the control group and other drug‐treated groups (Figure 7I,J). Altogether, CA‐4S_2_@ES‐Cu could initiate the release of DAMPs, activate ICD, and augment the anti‐tumor immune response, and also, CA‐4 dimers were identified for the first time to exert ICD‐inducing activity.

### 2.8 Antitumor Effects and Safety Profile of CA‐4S_2_@ES‐Cu In Vivo

**Figure 8:**
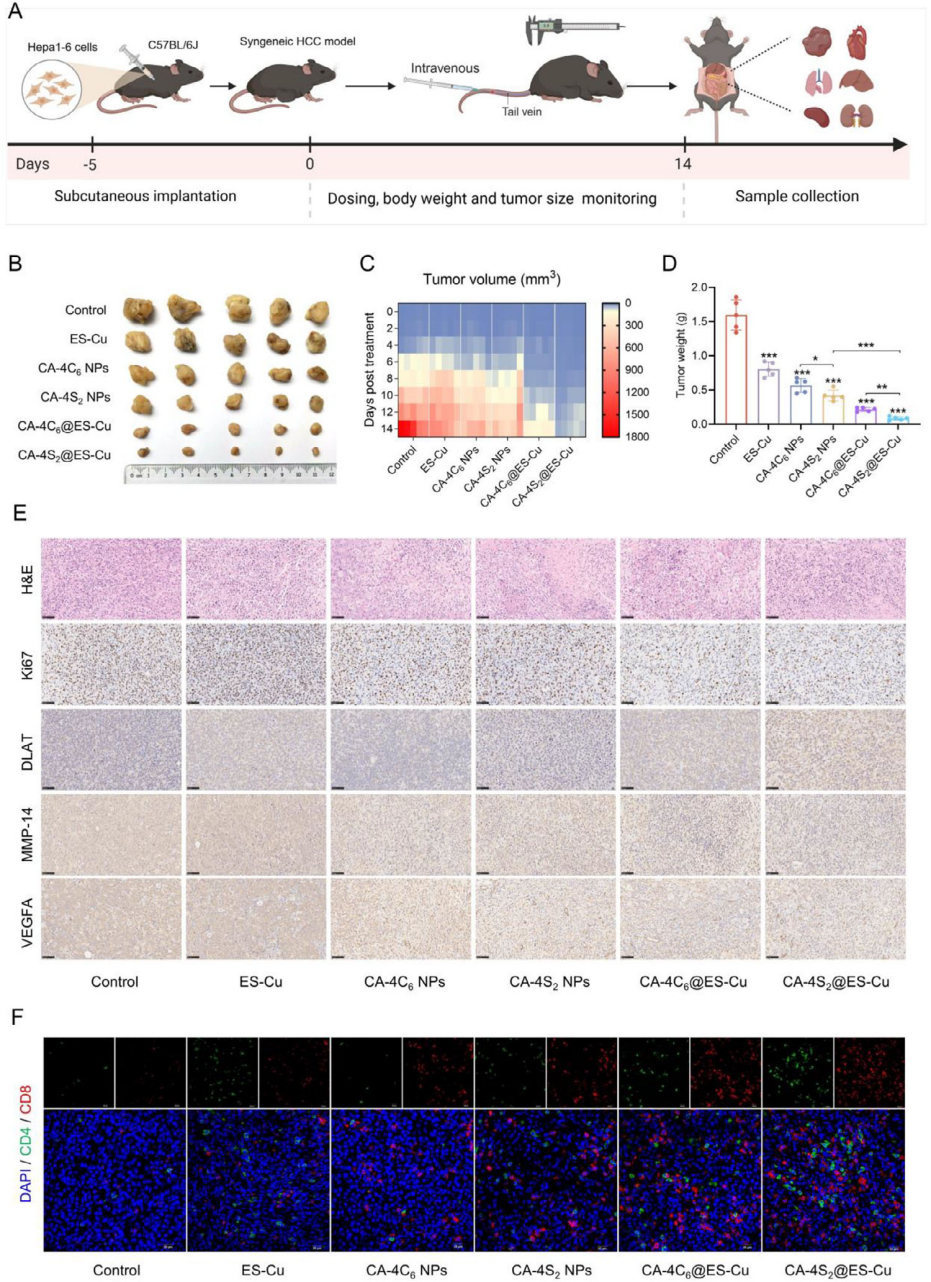
Antitumor effects of CA‐4S_2_@ES‐Cu in a subcutaneous HCC model. A) Illustration for development of HCC tumor models and treatment programs. B) Representative images of tumor tissues from different treatment groups. C) Variation in tumor volume during the treatment period. D) Tumor weight after 14 days of treatment. E) Representative H&E staining images of tumor tissues and immunohistochemical staining for Ki67, DLAT, MMP‐14 and VEGFA. F) Representative immunofluorescence images of CD4/CD8 in tumor tissues. *p < 0.05, **p < 0.01, ***p < 0.001, versus the specified group.

All animal procedures were approved by the Experimental Animals Administrative Committee of Chengdu University of Traditional Chinese Medicine (Registration number: SYXK‐2020‐124, Chengdu, China) and conducted under relevant animal regulations. A syngeneic transplantation tumor model was established by subcutaneous injection of Hepa1‐6 cells into the axilla of C57BL/6J mice to evaluate the in vivo antitumor efficacy of CA‐4S_2_@ES‐Cu (**Figure** **8**A). As compared with the saline group, the ES‐Cu, CA‐4C_6_ NPs and CA‐4S_2_ NPs groups presented limited tumor growth suppression. However, the CA‐4C_6_@ES‐Cu and CA‐4S_2_@ES‐Cu groups performed superior tumor suppression than the other groups, with the CA‐4S_2_@ES‐Cu group exhibiting the most prominent tumor suppression, featuring up to 95% tumor growth inhibition (Figure 8B–D; Figure S22, Supporting Information). Additionally, GSH levels in the tumors of mice treated with CA‐4S_2_@ES‐Cu were dramatically decreased, and redox homeostasis was obviously disrupted in vivo (Figure S23, Supporting Information). Subsequently, H&E staining revealed tumor tissue damage in all treatment groups compared to the control group, with substantial and significant tissue destruction within CA‐4S_2_@ES‐Cu tumors. Meanwhile, by the Ki67 immunohistochemistry assay, CA‐4S_2_@ES‐Cu could effectively restrain the proliferation of tumor tissues. By detecting DLAT oligomerization in mouse tumor tissues, the CA‐4S_2_@ES‐Cu group displayed the most intense DLAT aggregation status, indicating that it effectively induced cuproptosis in vivo. Moreover, the CA‐4‐containing groups significantly suppressed the expression of MMP‐14 and VEGFA, which performed critical roles in tumor invasion and metastasis, indicating that CA‐4S_2_@ES‐Cu possessed the potential to inhibit tumor metastasis (Figure 8E). Additionally, CD4/CD8 expression in tumor tissues was assessed by immunofluorescence, the most and strongest red and green fluorescence was observed in the CA‐4S_2_@ES‐Cu treatment group (Figure 8F). In conclusion, CA‐4S_2_@ES‐Cu evoked cuproptosis and triggered an immune response to dramatically suppress tumor growth in vivo. Additionally, CA‐4S_2_@ES‐Cu exhibited a 2.98‐fold superior efficacy in suppressing tumor growth compared to sorafenib, a standard HCC treatment drug (Figure S24, Supporting Information).

Afterward, the body weights of the mice were measured during the treatment process and no statistically significant distinction existed between the treatment groups (Figure S25, Supporting Information). Meanwhile, the collected major organs were weighed and H&E stained, revealing no significant differences in organ indices among the groups and no evidence of tissue toxicity (Figures S26, S27, Supporting Information). Eventually, no significant variations in alanine aminotransferase (ALT), aspartate aminotransferase (AST), creatinine (CREA), blood urea nitrogen (BUN), and lactate dehydrogenase (LDH) were observed among groups via serum biochemical assays in mice (Figure S28, Supporting Information). The above outcomes demonstrated that CA‐4S_2_@ES‐Cu possessed favorable biosafety and was suitable for intravenous administration. Moreover, in long‐term toxicity assays, mice presented no notable weight loss after 15 consecutive administrations of CA‐4S_2_@ES‐Cu, whereas weight loss was apparent in mice in the sorafenib group (Figure S29, Supporting Information). Besides, the mice in the PBS and CA‐4S_2_@ES‐Cu groups exhibited normal status during the administration period, while the sorafenib‐administered group experienced slight hair loss. The liver serves as a primary organ for drug metabolism. As analyzed by H&E staining, the CA‐4S_2_@ES‐Cu group presented only a slight loosening of hepatocyte cytoplasm, without notable pathological alterations (Figure S30, Supporting Information). In the sorafenib group, vacuolization, nuclear rupture and crumpling of hepatocytes were observed, accompanied by an inflammatory response. Although ALT and AST levels were within the normal range in all groups of mice, ALP levels were dramatically elevated beyond the normal range in the sorafenib group (Figure S31, Supporting Information). The above outcomes further demonstrated that CA‐4S_2_@ES‐Cu performed a superior safety profile and exerted considerably less toxic effects on the liver compared to sorafenib.

### 2.9 In Vivo Anti‐Tumor Immunity Profile of CA‐4S_2_@ES‐Cu

**Figure 9:**
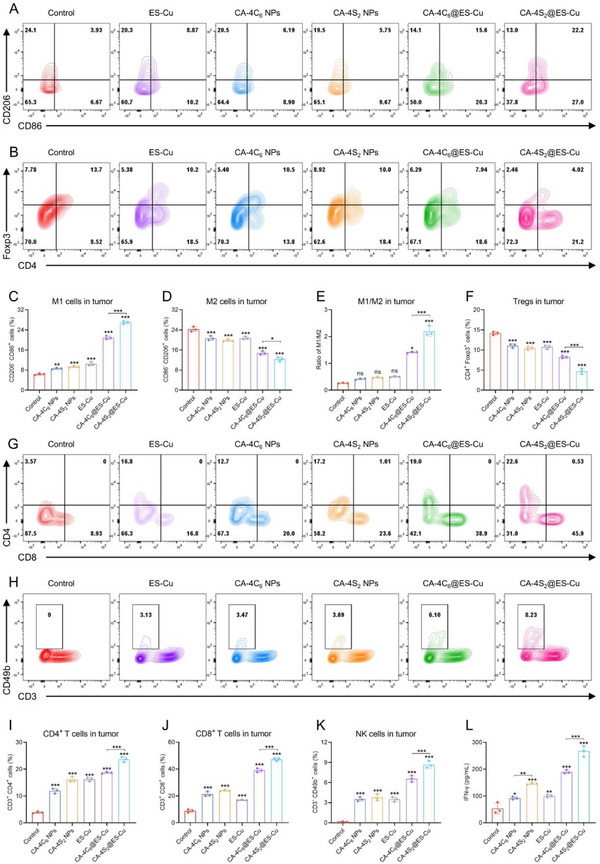
In vivo anti‐tumor immune analysis of CA‐4S_2_@ES‐Cu. A) Flow cytometry analysis of macrophages in tumor tissues. B) Flow cytometry analysis of Tregs in tumor tissues. C) Quantitative analysis of M1 macrophages (gated on CD11b^+^ F4/80^+^ CD86^+^ CD206^−^). D) Quantitative analysis of M2 macrophages (gated on CD11b^+^ F4/80^+^ CD206^+^ CD86^−^). (E) Ratio of M1 to M2 macrophages. F) Quantitative analysis of Tregs (gated on CD3^+^ CD4^+^ Foxp3^+^). G) Flow cytometry analysis of CD4^+^ and CD8^+^ T cells in tumor tissues. H) Flow cytometry analysis of NK cells in tumor tissues. I) Quantitative analysis of CD4^+^ T cells (gated on CD45^+^ CD4^+^). J) Quantitative analysis of CD8^+^ T cells (gated on CD45^+^ CD8^+^). K) Quantitative analysis of NK cells (gated on CD3^−^ CD49b^+^). L) IFN‐γ levels in tumor tissues. Note: All cells were pre‐gated on FVS780^−^ and CD45^+^. *p < 0.05, **p < 0.01, ***p < 0.001, versus the specified group.

Building on the confirmed remarkable suppression of tumor growth by CA‐4S_2_@ES‐Cu, its systemic modulation of the tumor immune microenvironment (TIME) was further explored. Tumor‐associated macrophages (TAMs), one of the most abundant immune cell populations in TIME, exhibit phenotypic polarization (M1 or M2) that critically influences tumor progression and therapeutic response. Encouragingly, CA‐4S_2_@ES‐Cu dramatically promoted the polarization of TAMs toward the M1‐type phenotype while suppressing the ratio of the M2‐type phenotype, in which the M1/M2 ratio amounted to 2.20 ± 0.19, suggesting its capability to efficiently reprogram the conversion of TAMs toward an anti‐tumor phenotype (**Figure** **9**A,C–E). Furthermore, regulatory T cells (Tregs), as key components of the immunosuppressive tumor microenvironment, facilitate tumor immune evasion by inhibiting the function of effector T cells. CA‐4S_2_@ES‐Cu treatment significantly reduced the proportion of Tregs, decreasing from 13.70% in the control group to 4.02%, suggesting that CA‐4S_2_@ES‐Cu effectively alleviated immunosuppression and enhanced the antitumor activity of effector T cells (Figure 9B,F). Simultaneously, the infiltration of CD4^+^ and CD8^+^ T cells in tumor tissues was notably augmented after treatment with CA‐4S_2_@ES‐Cu, accounting for 22.6% and 45.9%, respectively, which further strengthened the anti‐tumor immune response (Figure 9G,I,J). NK cells, essential components of the innate immune system, directly eliminate tumor cells through the release of perforin and granzymes and secrete immunomodulatory factors (e.g., IFN‐γ) to activate other immune cells.^[^ ^38^ ^]^ Following CA‐4S_2_@ES‐Cu treatment, the proportion of NK cells in tumor tissues significantly increased to 8.23% (Figure 9H,K). Upon ELISA assay, CA‐4S_2_@ES‐Cu treatment dramatically elevated the level of IFN‐γ in tumor tissues (Figure 9L), lending further support to its activating effect on the local immune response. Notably, IFN‐γ levels in mouse serum remained without notable variations, likely due to the complexity of systemic self‐regulation, indicating that CA‐4S_2_@ES‐Cu primarily enhanced localized immune responses at the tumor site without inducing widespread systemic inflammation (Figure S32, Supporting Information). In summary, CA‐4S_2_@ES‐Cu orchestrated multidimensional modulation of TIME, including promoting M1 macrophage polarization, reducing Treg proportions, increasing CD4^+^ and CD8^+^ T cell infiltration, enhancing NK cell activity, and elevating IFN‐γ levels, demonstrating significant immune activation.

### 2.10 CA‐4S_2_@ES‐Cu Suppressed HCC Cell Migration, Invasion, and Metastasis

**Figure 10:**
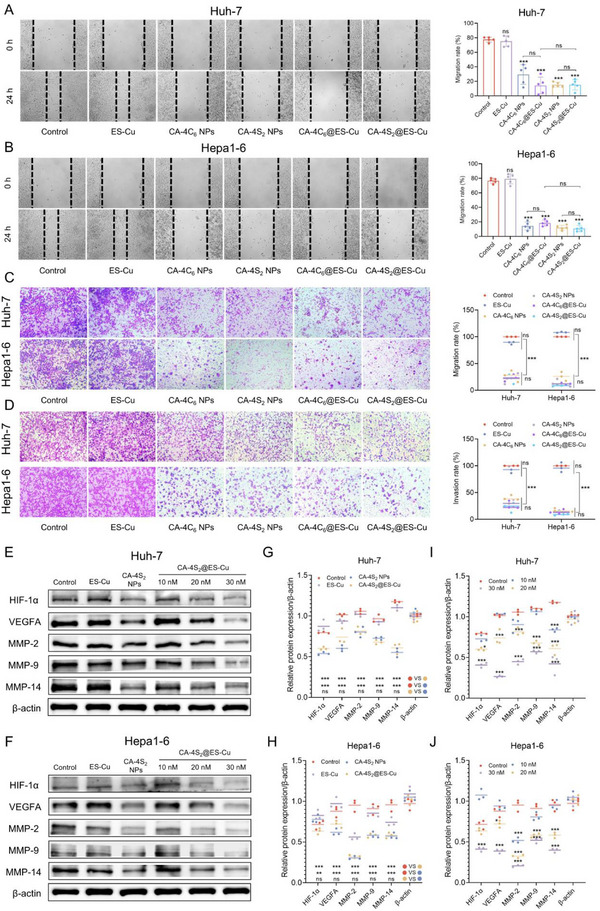
CA‐4S_2_@ES‐Cu suppressed HCC cell migration, invasion and metastasis. A,B) Wound healing ability of HCC cells after various drug treatments. C) Migration ability of HCC cells after various drug treatments. D) Invasion ability of HCC cells after various drug treatments. E,F) Expression levels of proteins related to tumor metastasis in HCC cells after various drug treatments. G–J) Quantitative analysis of protein expression related to cancer metastasis in HCC cells after various drug treatments. *p < 0.05, **p < 0.01, ***p < 0.001, versus the specified group.

The formation of colonies and the process of metastasis were essential properties of tumor survival and progression.^[^ ^39^, ^40^ ^]^ Previous experiments demonstrated that CA‐4S_2_@ES‐Cu effectively inhibited the formation of colonies by HCC cells (Figure 5E,F). Moreover, the primary cause of hyper‐mortality in liver cancer is metastasis, and cell migration and invasion are critical processes in cancer metastasis. CA‐4 is a potent inhibitor of tumor cell proliferation, migration and invasion.^[^ ^41^ ^]^ In comparison with the control group, the wound healing rate of HCC cells after ES‐Cu treatment presented no notable change; however, following CA‐4C_6_ NPs, CA‐4S_2_ NPs, CA‐4C_6_@ES‐Cu, and CA‐4S_2_@ES‐Cu treatments, the wound healing rate of HCC cells was dramatically diminished, with no significant distinction among these four groups (**Figure** **10**A,B). Additionally, the outcome of the cell migration assay was consistent with this (Figure 10C). Similarly, there was no significant alteration in the invasion rate of HCC cells after ES‐Cu treatment, however, the invasion capability of HCC cells was dramatically attenuated by CA‐4C_6_ NPs, CA‐4S_2_ NPs, CA‐4C_6_@ES‐Cu, and CA‐4S_2_@ES‐Cu treatments, with a decrease of ≈60–90% (Figure 10D). Previous evidence has supported that copper ions promoted cancer invasion and metastasis. Despite the capacity of ES to transport large amounts of copper ions into cells, the ES‐Cu group presented no promotion of HCC cell migration and invasion, which probably correlated with the depletion of copper ions by amplifying cuproptosis, and indicated that CA‐4 in nanomedicines performed a primary role in inhibiting HCC cell migration and invasion.

MMP‐2, MMP‐9 and MMP‐14 are intimately associated with cell migration and invasion and serve as drivers of extracellular matrix and tissue destruction.^[^ ^42^ ^]^ Moreover, HIF1‐α facilitates angiogenesis by inducing VEGFA expression.^[^ ^43^ ^]^ After treating HCC cells by ES‐Cu, CA‐4S_2_ NPs and CA‐4S_2_@ES‐Cu with the same molar dose (20 nm), the protein expression in the ES‐Cu group was almost non‐significantly changed when compared with that in the control group, whereas the five protein expressions in the CA‐4S_2_ NPs and CA‐4S_2_@ES‐C groups were all remarkably down‐regulated and non‐systematically difference between the groups (Figure 10E,F). Besides, MMP‐2/9/14, HIF1‐α, and VEGFA protein levels were statistically dramatically declined in a CA‐4S_2_@ES‐Cu dose‐dependent manner (Figure 10G–J). In summary, our findings demonstrated that CA‐4S_2_@ES‐Cu could effectively suppress HCC cell migration, invasion, and metastasis.

### 2.11 CA‐4S_2_@ES‐Cu Suppressed HCC Angiogenesis and Metastasis

Given the pro‐angiogenic and metastatic capacity of copper ions, modulation of copper metabolism probably provides a promising pathway for inhibiting HCC metastasis (Figure 1F). Meanwhile, CA‐4, classified as a typical vascular destructive agent, could selectively damage the vascular endothelial cells in tumor tissues, leading to vascular closure.^[^ ^25^ ^]^ Accordingly, the anti‐angiogenic and metastasis inhibitory capabilities of CA‐4S_2_@ES‐Cu were investigated in vitro and in vivo.

**Figure 11:**
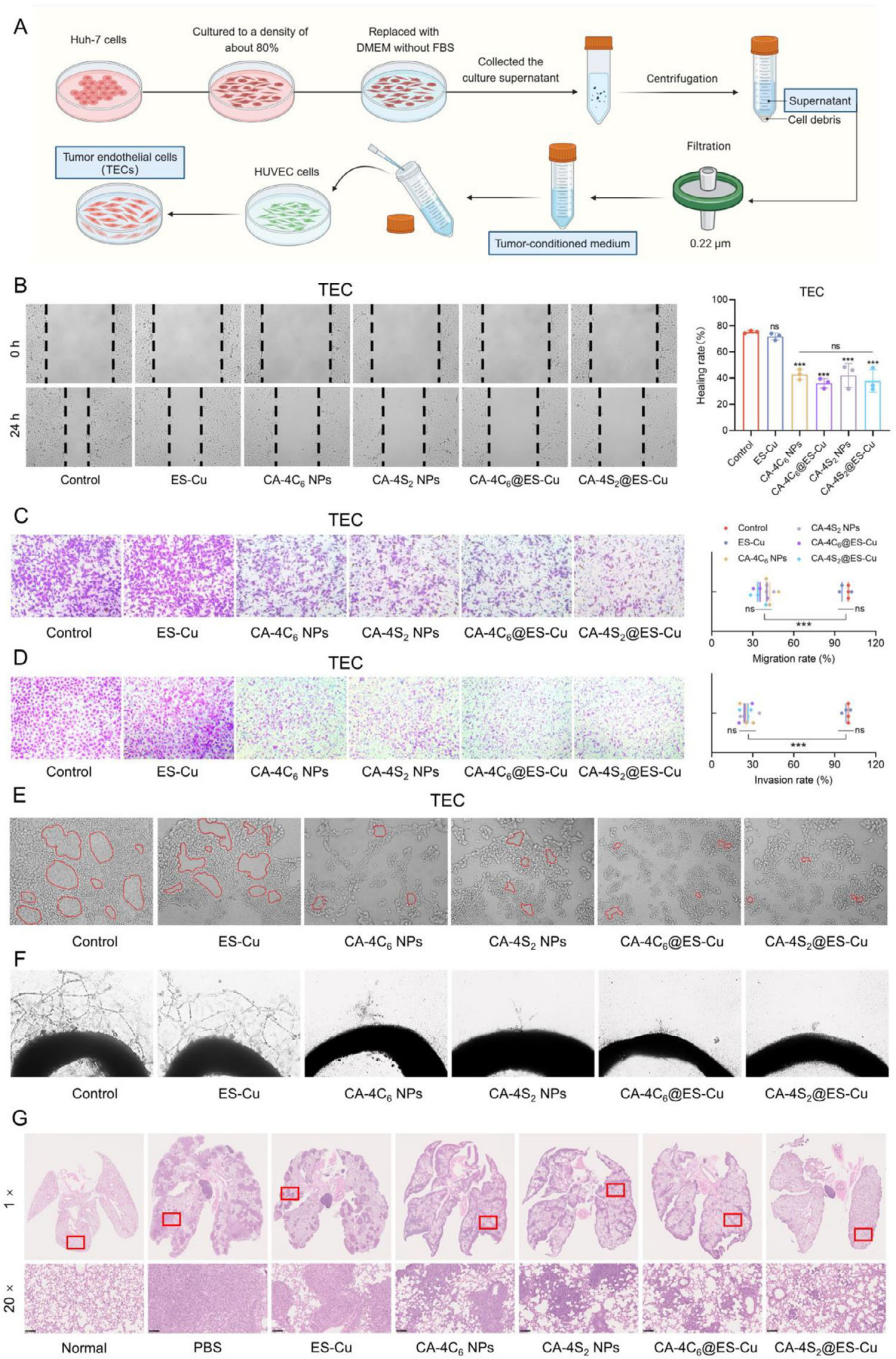
CA‐4S_2_@ES‐Cu suppressed angiogenesis and metastasis in HCC. A) Schematic diagram of tumor endothelial cell induction. B) The wound healing capacity of TEC cells after various drug treatments. C) The migration capacity of TEC cells after various drug treatments. D) The invasion potential of TEC cells after various drug treatments. E) The tube formation capacity of TEC cells after various drug treatments. F) The sprouting capacity of rat aortic rings following various drug treatments. G) Representative images and H&E staining of lung tissues from mice treated with different regimens. ***p < 0.001, versus the specified group.

Human umbilical vein endothelial cells (HUVECs) were induced to differentiate into tumor endothelial cells (TECs) through a supernatant of Huh‐7 cells, and the wound healing ability of TECs was examined by scratch assay (**Figure** **11**A).^[^ ^44^ ^]^ Compared with the control group, wound healing rate of HCC cells was not affected remarkably after ES‐Cu treatment, whereas the wound healing capability of TECs was greatly impaired by CA‐4C_6_ NPs, CA‐4S_2_ NPs, CA‐4C_6_@ES‐Cu, and CA‐4S_2_@ES‐Cu treatments without any obvious distinction (Figure 11B). As well, the CA‐4‐containing groups were able to effectively suppress the TEC cell migration, achieving an ≈60% reduction in the migration ability of TECs compared to the control group (Figure 11C). Similarly, the capacity of TEC cell invasion was dramatically attenuated after treatment in the groups containing CA‐4 compared to the control group, with no significant difference among the four (Figure 11D). Above results indicated that CA‐4 exerted an essential role in the suppression of TEC cell migration and invasion, which was consistent with the previous conclusion that CA‐4 was able to inhibit the migration and invasion of HCC cells.

Endothelial tube formation is a crucial process in tumor angiogenesis, and the tube‐forming capacity of TECs after ES‐Cu treatment exhibited no notable alteration; however, after CA‐4C_6_ NPs, CA‐4S_2_ NPs, CA‐4C_6_@ES‐Cu, and CA‐4S_2_@ES‐Cu treatments, the plurality of TECs aggregated into small clusters, with little tube‐forming capacity (Figure 11E). Simultaneously, with the assistance of rat aortic ring assay, the control and ES‐Cu groups were found to form an elaborate microvascular network around the treated aortic rings, whereas the groups containing CA‐4 nearly completely repressed the aortic ring germination (Figure 11F). Additionally, in the lung metastasis model established by injecting Hepa1‐6 cells through the tail vein, densely aggregated metastatic nodules with a large and tightly spaced solid growth pattern were observed in lung tissues of both the PBS and ES‐Cu groups (Figure 11G). Although tumor metastasis was visualized in the CA‐4‐containing treatment group, the nodules exhibited diffuse distribution characteristics. Strikingly, the CA‐4S_2_@ES‐Cu treatment group manifested the most superior anti‐metastatic effect, with only scattered tiny tumor nodules visible in the lung tissue. Collectively, CA‐4S_2_@ES‐Cu could effectively suppress HCC angiogenesis and metastasis.

## 3 Conclusion

In conclusion, we presented a novel therapeutic strategy for HCC by constructing CA‐4S_2_@ES‐Cu nanomedicines to amplify cuproptosis for intensifying the efficacy of cancer chemotherapy and immunotherapy. In response to the high GSH concentration in tumor cells, CA‐4S_2_@ES‐Cu could release CA‐4 and ES‐Cu, which were capable of exerting synergistic effects and striking multiple impacts on tumor cells. In particular, CA‐4 suppressed cellular microtubule polymerization, blocked tumor cell mitosis, and induced ROS generation. Additionally, CA‐4 was able to down‐regulate the HIF‐1α expression, restrain the neovascularization of tumors, and then interrupt the nutrient supply and restrict the tumor metastasis. Copper ions delivered by ES‐Cu within cellular mitochondria were reduced to Cu^+^ by FDX1, triggering DLAT oligomerization and mitochondrial metabolic disturbances with massive ROS accumulation. Moreover, the process of CA‐4S_2_ rupture to release CA‐4 depleted GSH, which further diminished the cellular antioxidant capacity, lessened the scavenging effect of GSH on Cu^+^, and dramatically augmented cuproptosis and intracellular oxidative stress. Simultaneously, CA‐4 and ES‐Cu jointly evoked the release of DAMPs signaling molecules such as CRT, HMGB1, and ATP, reversing the immunosuppressive tumor environment, which in turn triggered ICD and activated the T‐cell‐mediated immune response, thus further enhancing the anti‐tumor efficacy. This study achieved the integration of chemotherapy and immunotherapy with cuproptosis, which not only solved the deterioration and metastasis of HCC caused by copper ion overload, but also compensated for the deficiency of CA‐4's feeble lethality against residual tumor tissues, and provided a promising and comprehensive strategy for HCC with significant potential. The hybridized prodrug dimer and nanoassembly technology adopted in this study possesses certain potential for large‐scale production. However, systematic research on the optimization of process parameters, the establishment of quality control standards, and the stability of large‐scale production is warranted to guarantee the controllability and feasibility of the process during industrialization.

## Conflict of Interest

The authors declare no conflict of interest.

## Author Contributions

Y.Z., Y.C., S.R., C.Z., J.L., K.L. contributed equally to this work. L.J., Z.C.T. and H.Y. designed and supervised the experiments; Z.Y.J., C.Y.N., R.S.M., Z.C.Z., and L.K. conducted the experiments and drafted the manuscript; L.J.N., W.Y., C.H.Y., Z.F.J., Y.X.L., G.X., Z.T.T., and W.T.B. carried out some experiments and evaluated the data; L.J. and L.D.F. revised this paper. All authors gave their approval to the final manuscript version.

## Supporting information



Supporting Information

## Data Availability

The data that support the findings of this study are available from the corresponding author upon reasonable request.
