# Supplementary material for: Responsive ROS‐Augmented Prodrug Hybridization Nanoassemblies for Multidimensionally Synergitic Treatment of Hepatocellular Carcinoma in Cascade Assaults
Source: Adv Sci (Weinh). 2025 May 5;12(29):2501420. doi: 10.1002/advs.202501420 (PMC12362818; doi:10.1002/advs.202501420)
Supplement: Supplementary file 1 — Supporting Information [file ADVS-12-2501420-s001.docx]

**Supporting information**

**Responsive ROS-augmented prodrug hybridization nanoassemblies for multidimensionally synergitic treatment of hepatocellular carcinoma in cascade assaults**

*Yingjie Zeng^a, 1^, Yuening Cao^a, 1^, Senmiao Ren^a, 1^, Chaozheng Zhang^a, 1^, Jianan Liu^a, 1^, Ke Liu**^b, 1^, Yan Wang^a,^, Hongyu Chen^a, c^, Fengjiao Zhou^b^, Xiuli Yang^b^, Xian Ge^a^, Tingting Zhang^a^, Tianbao Wang^d^,* *Yao He^a, *^, Defang Li^c, *^, Chuantao Zhang^b, *^, Jun Lu**^a, *^*

1. State Key Laboratory of Southwestern Chinese Medicine Resources, School of Pharmacy, Chengdu University of Traditional Chinese Medicine, Chengdu, China;
2. Department of Respiratory Medicine, Hospital of Chengdu University of Traditional Chinese Medicine, Chengdu, China;
3. College of Pharmacy, Hebei University of Chinese Medicine, Shijiazhuang, China;
4. Sichuan Clinical Research Center for Cancer, Sichuan Cancer Hospital & Institute, Sichuan Cancer Center, Affiliated Cancer Hospital of University of Electronic Science and Technology of China, Chengdu, China.

^1^ These authors contributed equally to this work.

* Corresponding addressed to: Jun Lu, ljaaa111@163.com; Chuantao Zhang, zhangchuantao@cdutcm.edu.cn; Defang Li, lidefang@163.com; Yao He, heyao2010@cdutcm.edu.cn.

**Contents**

**Synthesis and characterization of CA-4 prodrugs** 3

**Synthetic Schemes** 6

**Methods and materials** 8

**Supplementary figures (S1-S10)** 21

**^1^H NMR, ^13^C NMR analysis of synthesized compounds** 27

**Supplementary figures (S17-S31)** 33

**Supplementary Table** 39

**Synthesis and characterization of CA-4 prodrugs**

***Synthesis of 3,4,5-trimethoxybenzyl-triphenylphosphonium bromide*** **(2)**

Triphenylphosphine hydrobromide (10.4 g, 30.3 mmol) was added to a solution of 3,4,5-trimethoxybenzyl alcohol **(1)** (5.0 g, 25.2 mmol) in acetonitrile (150 mL). The mixture was heated to 80 ℃ for 4 h. TLC indicated the completion of the reaction. After the evaporation of the solvents, the reaction mixture yielded a crude phosphonium salt (13.0 g, 98.5% yield) as a white solid. The crude product was used directly without the next purification. HRMS (ESI) *m/z* for C_28_H_28_O_3_P^+^ [M]: 443.1776.

***Synthesis of 3-((tert-butyldimethylsilyl) oxy)-4-methoxybenzaldehyde (3)***

Tert-Butyldimethylsilyl chloride (TBDMSCl) (4.2 g, 27.7 mmol) was added portionwise to a solution of 3-hydroxy-4-methoxybenzaldehyde (3.5 g, 23.0 mmol) and Et_3_N (5 mL, 34.5 mmol) in CH_2_Cl_2_ (150 mL), and the resultant mixture was stirred at room temperature for 6 h. After completion of the reaction, the mixture was poured onto ice water (100 mL), and then extracted with CH_2_Cl_2_ (300 mL). The solution was washed with 0.5 M HCl (2 × 50 mL), and saturated brine (3 × 50 mL) successively. The organic phase was dried over sodium sulfate and filtered, followed by the removal of the solvent under reduced pressure. The crude product was purified by column chromatography on silica gel (PE/EA = 5:1) to obtain compound **3** (5.9 g, 96.3% yield) as a yellow oil. **^1^H NMR (600 MHz, CDCl_3_)** δ 9.72 (s, 1H), 7.37 (dd, *J* = 8.3, 2.0 Hz, 1H), 7.29 (d, *J* = 2.0 Hz, 1H), 6.86 (d, *J* = 8.3 Hz, 1H), 3.78 (s, 3H), 0.92 (s, 9H), 0.09 (s, 6H). **^13^C NMR (151 MHz, CDCl_3_)** δ 190.13, 155.96, 144.93, 129.59, 125.78, 119.22, 110.59, 54.86, 25.02, 17.78. HRMS (ESI) *m/z* for C_14_H_22_NaO_3_Si [M+Na]^+^: 289.1230.

***Synthesis of (Z)-tert-butyl(2-methoxy-5-(3,4,5-trimethoxystyryl) phenoxy) dimethylsilane (4).***

NaH (2.4 g, 99.4 mmol) was added to a solution of compound **2** (13.0 g, 24.8 mmol) in CH_2_Cl_2_ (100 mL) at 0 ℃, and the mixture resultant was stirred at 0 ℃ for 0.5 h. Then compound **3** (5.8 g, 21.8 mmol) in CH_2_Cl_2_ (50 mL) was added dropwise to the reaction, and the mixture was stirred at room temperature overnight. Upon complete consumption of compound **3** detected by TLC analysis (PE/EA = 5:1), the reaction mixture was diluted with ice water (100 mL) and extracted with CH_2_Cl_2_ (3 × 80 mL). The solution was washed with brine (3 × 100 mL). The organic phase was dried over sodium sulfate and filtered, followed by the removal of the solvent under reduced pressure. The crude product was purified by column chromatography on silica gel (PE/EA = 20:1) to obtain compound **4** (5.5 g, 51.4% yield) as a yellow oil. **^1^H NMR (600 MHz, CDCl_3_)** δ 6.85 (dd, *J* = 8.3, 2.2 Hz, 1H), 6.79 (d, *J* = 2.1 Hz, 1H), 6.73 (d, *J* = 8.3 Hz, 1H), 6.50 (s, 2H), 6.48 – 6.40 (m, 2H), 3.83 (s, 3H), 3.78 (s, 3H), 3.70 (s, 6H), 0.93 (s, 9H), 0.06 (s, 6H). **^13^C NMR (151 MHz, CDCl_3_)** δ 152.96, 150.30, 144.62, 136.99, 133.12, 130.05, 129.71, 128.77, 122.86, 121.28, 111.65, 105.87, 60.90, 55.90, 55.52, 25.64, 18.36. HRMS (ESI) *m/z* for C_24_H_35_O_5_Si [M+H]^+^: 431.2252.

***Synthesis of (Z)-2-methoxy-5-(3,4,5-trimethoxystyryl) phenol (CA-4)***

Tetrabutylammonium fluoride (TBAF) (6.5 g, 23.2 mmol) was added to a solution of compound **4** (2.5 g, 5.8 mmol) in tetrahydrofuran (THF) (50 mL) at 0 ℃, and the mixture was cooled in an ice-brine bath and stirred for 4 h. In a separatory funnel, cold saturated ammonium chloride (25 mL) was added, and the reaction mixture was poured in. The aqueous layer was extracted with ethyl acetate (4 × 50 mL). The organic phase was washed with brine (3 × 50 mL), dried with magnesium sulfate, and filtered. The crude product was purified by column chromatography on silica gel (PE/EA = 4:1) to obtain CA-4 (1.6 g, 87.1% yield) as a white solid. **^1^H NMR (600 MHz, DMSO-*d*_6_)** δ 8.90 (d, *J* = 1.7 Hz, 1H), 6.81 (dd, *J* = 8.4, 2.3 Hz, 1H), 6.77 (d, *J* = 1.9 Hz, 1H), 6.66 (dd, *J* = 8.1, 2.3 Hz, 1H), 6.55 (d, *J* = 1.9 Hz, 2H), 6.42 – 6.32 (m, 2H), 3.70 (d, *J* = 2.0 Hz, 3H), 3.61 (d, *J* = 1.9 Hz, 3H), 3.57 (d, *J* = 2.1 Hz, 6H). **^13^C NMR (151 MHz, DMSO-*d*_6_)** δ 152.97, 147.55, 146.61, 137.13, 132.75, 130.00, 129.92, 128.71, 120.57, 115.99, 112.45, 106.49, 60.50, 56.01. HRMS (ESI) *m/z* caled for C_18_H_20_O_5_Na [M+Na]^+^: 339.1205.

***Synthesis of*** ***3,3'-disulfanediyldipropionic acid (5)***

3-Mercaptopropionic acid (1.00 g), copper(II) chloride dihydrate (161.0 mg, 0.9 mmol, 0.1 equiv), and TMEDA (4.4 g, 37.7 mmol, 4 equiv) were added to a 100 mL round-bottom flask and dissolved in 40 mL acetonitrile. The reaction mixture was stirred continuously for 12 h. Subsequently, 10 mL of 1 M NaOH solution was slowly added to the reaction system, followed by three extractions with ethyl acetate. The aqueous layer was then acidified to pH 2 with 2 M HCl and extracted five times with ethyl acetate. The organic phases were combined, dried over anhydrous sodium sulfate, and concentrated under reduced pressure to yield the product (1.9 g, 95.9% yield) as a white solid, which was used directly in the subsequent reaction. **^1^H NMR (600 MHz, DMSO-*d_6_*)** δ 2.87 (t, *J* = 6.9 Hz, 4H), 2.61 (t, *J* = 6.9 Hz, 4H). **^13^C NMR (151 MHz, DMSO-*d_6_*)** δ 173.14, 34.03, 33.47. HRMS (ESI) *m/z* for C_6_H_10_O_4_S_2_ [M+H]^+^: 211.0096.

***Synthesis of CA-4C_6_***

Suberic acid (137.7 mg, 0.8 mmol), EDC·HCI (302.9 mg, 1.6 mmol), and DMAP (48.3 mg, 0.4 mmol) were dissolved in Dichloromethane (DCM) (50 mL). CA-4 (500.0 mg, 1.6 mmol) was added to this solution with stirring at 0 ℃ for 6 h. Upon complete consumption of compound CA-4 detected by TLC (PE/EA = 2:1), the solution was washed with saturated NH_4_Cl solution (3 × 20 mL) and saturated brine (1 × 20 mL). The organic phase was dried over sodium sulfate and filtered, followed by the removal of the solvent under reduced pressure. The crude product was purified by column chromatography on silica gel (PE/EA = 3:1) to obtain CA-4C_6_ (450.0 mg, 73.8% yield) as a white solid. **^1^H NMR (600 MHz, DMSO-*d*_6_)** δ 7.14 (dd, *J* = 8.6, 2.1 Hz, 2H), 7.06 (d, *J* = 8.5 Hz, 2H), 6.97 (d, *J* = 2.1 Hz, 2H), 6.53 (s, 4H), 6.51 – 6.46 (m, 4H), 3.73 (s, 6H), 3.63 (s, 6H), 3.60 (s, 12H), 2.51 (d, *J* = 3.6 Hz, 2H), 2.49 (s, 2H), 1.59 (dd, *J* = 8.8, 5.3 Hz, 4H), 1.38 – 1.32 (m, 4H). **^13^C NMR (151 MHz, DMSO-*d*_6_)** δ 171.51, 153.10, 150.54, 139.44, 137.22, 132.45, 129.93, 129.80, 128.72, 127.90, 123.08, 113.10, 106.30, 60.49, 56.27, 55.99, 33.49, 28.29, 24.77. HRMS (ESI) *m/z* for C_44_H_51_O_12_ [M+H]^+^: 771.3378.

***Synthesis of CA-4S_2_***

3,3'-dithiodipropionic acid (168.0 mg, 0.8 mmol), EDC·HCI (306.3 mg, 1.6 mmol), and DMAP (48.8 mg, 0.4 mmol) were dissolved in DCM (50 mL). CA-4 (505.5 mg, 1.6 mmol) was added to this solution with stirring at 0 ℃ for 8 h. Upon complete consumption of compound CA-4 detected by TLC analysis (PE/EA = 5:1), the solution was washed with saturated NH_4_Cl solution (3 × 20 mL), and saturated brine (1 × 20 mL) successively. The organic phase was dried over sodium sulfate and filtered, followed by the removal of the solvent under reduced pressure. The crude product was purified by column chromatography on silica gel (PE/EA = 5:1) to obtain CA-4S_2_ (550.0 mg, 85.3% yield) as a white solid. **^1^H NMR (600 MHz, CDCl_3_)** δ 7.12 (dd, *J* = 8.4, 2.1 Hz, 2H), 7.01 (d, *J* = 2.1 Hz, 2H), 6.84 (d, *J* = 8.5 Hz, 2H), 6.49 (s, 4H), 6.44 (s, 4H), 3.83 (d, *J* = 1.2 Hz, 6H), 3.79 (s, 6H), 3.70 (s, 12H), 3.04 – 3.01 (m, 4H), 3.00 – 2.96 (m, 4H). **^13^C NMR (151 MHz, CDCl_3_)** δ 169.67, 152.99, 150.13, 139.31, 137.18, 132.42, 130.12, 129.61, 128.52, 127.81, 123.11, 112.02, 105.85, 60.92, 55.94, 33.87, 33.09. HRMS (ESI) *m/z* for C_42_H_47_O_12_S_2_ [M+H]^+^: 807.2505.

**Synthetic Schemes**

**Scheme S1** Synthesis of CA-4. **(a)** PPh_3_·HBr, acetonitrile, 80 ℃, 4 h. **(b)** NaH, DCM, 0 ℃ to rt, 8 h. **(c)** TBAF, THF, 0 ℃.

**Scheme S2** Synthesis of 3,3'-Disulfanediyldipropionic acid (5). **(a)** 3-Mercaptopropionic acid, copper (II) chloride dihydrate, TMEDA, 25 ℃, 12h.

**Scheme S3** Synthesis of CA-4C_6_. **(a)** Suberic acid, EDC·HCl, DMAP, 0 ℃, 6 h.

**Scheme S4** Synthesis of CA-4S_2_ **(a)** 3,3'-Dithiodipropionic acid, EDC·HCl, DMAP, 0 ℃, 8 h.

**Methods and materials**

**Materials**

ES was obtained from Rhawn (Chengdu, China). DSPE-PEG_2K_ was bought from Aladdin (Shanghai, China). CuCl_2_, trimethoxybenzyl alcohol, triphenylphosphine hydrobromide, 3-hydroxy-4-methoxybenzaldehyde, TBDMSCl, NaH, Et_3_N, TBAF, suberic acid, 3,3'-dithiodipropionic acid, N-(3-Dimethylaminopropyl)-N'-ethylcarbodiimide hydrochloride (EDC·HCI), 4-Dimethylaminopyridine (DMAP) were all purchased from Shanghai Yuanye Biotechnology Co., Ltd (Shanghai, China). Reactions were monitored by the TLC and visualized under UV light (254 nm). The flash column chromatography was performed using silica gel (200-300 mesh). RPMI 1640 and DMEM medium were purchased from Corning (New York, USA). Fetal bovine serum (FBS) was supplied by ExCell Bio (Shanghai, China). Penicillin-streptomycin and trypsin were provided by Biofrox (Cambridge, UK). Annexin V-FITC Apoptosis Assay Kit, ATP Assay Kit, Reactive Oxygen Species Assay Kit, Enhanced Mitochondrial Membrane Assay Kit with JC-1, EdU Assay Kit, Cell Cycle and Apoptosis Analysis Kit, Total Glutathione Assay Kit, and Tubulin-Tracker Green Staining Kit were provided by Beyotime (Shanghai, China). Alexa Fluor 647–conjugated goat anti-rabbit secondary antibody, Cellular Cuprous Fluorometric Assay Kit (Cu^+^), Cell Copper (Cu^2+^) Colorimetric Assay Kit, and Mouse IFN-γ (Interferon Gamma) ELISA Kit were provided by Elabscience (Wuhan, China). Antibodies against MMP-2, MMP-9, MMP-14, HIF1-α, and VEGFA were bought from Cell Signaling Technology (Boston, USA). Antibodies against DLAT and FDX1 were bought from Abmart (Shanghai, China). Antibodies against β-Actin, HMGB1, and CRT were obtained by Servicebio (Wuhan, China). Flow Cytometry Antibodies against CD3, CD4, and CD8 were obtained by 4A Biotech (Beijing, China). Other solvents and reagents were purchased from Jinshan chemical reagent (Chengdu, China), which are of analytical grade and used directly. Portions of the figures in this manuscript were created using BioRender (http://biorender.com/).

**Methods**

***Preparation of CA-4S_2_@ES-Cu***

ES (6.0 mg), CA-4S_2_ (12.5 mg), and DSPE-PEG_2K_ (10.0 mg) were dissolved in DCM (300 μL). To this solution, CuCl_2_ (1.1 mg) was added and stirred for 2 h. The unreacted CuCl_2_ was removed by filtration, and the resultant mixture was then added dropwise into pure water (20 mL) under stirring. The solution was stirred for another 2 h and then dialyzed for 24 h to remove DCM and other free small molecules such as ES-Cu, ultimately yielding a yellow CA-4S_2_@ES-Cu nanoparticle solution.

***Preparation of CA-4C_6_@ES-Cu***

ES (7.0 mg), CA-4C_6_ (14.0 mg), and DSPE-PEG_2K_ (40.0 mg) were dissolved in DCM (300 μL). To this mixture, CuCl_2_ (1.1 mg) was added and stirred for 12 h. The unreacted CuCl_2_ was removed by filtration and the resulting mixture was then added dropwise into pure water (20 mL) under stirring, followed by stirring for an additional 2 h. The mixture was dialyzed for 24 h to remove DCM and other free small molecules such as ES-Cu, ultimately yielding a yellow CA-4C_6_@ES-Cu nanoparticle solution.

***General measurements***

^1^H NMR spectra were recorded on a Bruker AV-600 NMR spectrometer. The molecular weight of the compounds was determined using high-resolution mass spectrometry (HRMS) on an Agilent 1260-Bruker tims TOF instrument. The morphology of the nanoparticles was investigated using TEM (Ruli, HT-7800, Japan). High-resolution transmission electron microscopy (HRTEM) (Thermo Fisher, TF-20, Japan) was used to evaluate the elemental composition of the nanoparticles. The particle size, size distribution, and zeta potential of the nanoparticles were determined by dynamic light scattering (DLS) using a Zeta-sizer Nano Analyzer (Malvern, U.K.). Ultraviolet-visible (UV-vis) absorption spectra were obtained using a UV-vis spectrophotometer (MAPADA, UV-3100PC). The concentration of CA-4-related formulations and their release profiles were determined using high-performance liquid chromatography (HPLC) (Shimadzu, CBM-20A). Fourier transform infrared (FTIR) spectroscopy (Thermo Scientific, Nicolet iN10, USA) was employed to characterize CA-4 formulations, with a wavenumber range of 500-4000 cm^-1^. X-ray photoelectron spectroscopy (XPS) (Shimadzu, AXI ULTRA DLD, Japan) was used to analyze the elemental composition of the nanoparticles.

***Drug Loading Efficiency***

Standard curves for ES, CA-4S_2_, and CA-4C_6_ were established using HPLC at a wavelength of 254 nm, with the concentration of CA-4S_2_, CA-4C_6_, and ES as the x-axis and the peak area as the y-axis. To analyze the encapsulated compounds, 1 mL of CA-4S_2_@ES-Cu or CA-4C_6_@ES-Cu was mixed with 1 mL of acetonitrile, and the mixture was vortexed for 20 minutes to disrupt the nanoparticle structure and release CA-4S_2_, CA-4C_6_, and ES. The resulting solution was then transferred to an HPLC vial for analysis. The drug loading content (DLC) and Encapsulation efficiency (EE) were calculated using the following formulas: DLC % = m (Encapsulated)/m (Total Nanoparticle Mass); EE % = m (Encapsulated)/m(Initial).

***Stability testing***

To evaluate the stability of the nanoparticles, 1 mL of CA-4S_2_@ES-Cu or 1 mL of CA-4C_6_@ES-Cu was added to phosphate-buffered saline (PBS, pH 7.4) and stored at 4 °C for 15 days. The change in particle size was monitored by DLS. Additionally, 1 mL of CA-4S_2_@ES-Cu or 1 mL of CA-4C_6_@ES-Cu was added to PBS (pH 7.4) containing 10% fetal bovine serum (FBS) or to PBS (pH 7.4) containing 10% rat plasma/heparin. These mixtures were incubated at 37 °C for 72 h, and the particle size changes were monitored by DLS.

***GSH-responsive release of CA-4S_2_ NPs and CA-4C_6_ NPs***

To evaluate the release of CA-4 from self-assembled nanoparticles, 1 mL of CA-4S_2_ NPs and 1 mL of CA-4C_6_ NPs solutions were each added to PBS (pH 7.4) with or without 10 mM GSH. The mixtures were incubated at 37℃ under constant stirring for 24 h. At predetermined time points, 200 μL samples were taken for HPLC quantitative analysis of CA-4 release.

***GSH-responsive release of*** ***CA-4S_2_@ES-Cu and CA-4C_6_@ES-Cu***

To assess the release profiles, 1 mL of nanoparticles was placed into a dialysis bag (MW = 3500) and immersed in 30 mL of release medium containing 0, 1, 5 or 10 mM GSH at 37 °C. At predetermined time intervals, the drug release was measured by HPLC at 254 nm. Chromatographic separation was performed on a C18 column (4.6 × 250 mm, 5 μm). The mobile phase consisted of a 75:25 (v/v) mixture of methanol and water, with a flow rate of 1 mL min⁻¹.

***Spectrophotometric titration of ES with Cu^2+^***

First, an ES solution (3 mM) was prepared, and 10 μL of this solution was added to 3 mL of PBS buffer containing 25% DMSO. A CuCl₂ solution was then added in a gradient concentration manner. The changes in absorbance were monitored using UV-Vis spectrophotometry.

***Bioinformatics analysis***

We downloaded STAR-counts data and corresponding clinical information for LIHC from the TCGA database (https://portal.gdc.cancer.gov). We then extracted data in TPM format and performed normalization using the log2(TPM+1) transformation. The TCGA-LIHC standardized pan-cancer dataset was derived from UCSC (https://xenabrowser.net/). The protein expression data were collected from Human Protein Atlas ( https://www.proteinatlas.org). GEPIA2 (http://gepia2.cancer-pku.cn) provides tumor/normal differential expression and survival analysis.

***Pharmacokinetics (PK) of CA-4S_2_@ES-Cu.***

Intravenous administration of CA-4S_2_@ES-Cu (5 mg/kg, based on CA-4 content) was performed in C57BL/6 mice (8 weeks old, male, tumor volume: 150 mm³) bearing Hepa1-6 tumors. Blood samples were collected via orbital sinus puncture at predetermined time points of 1 min, 5 min, 15 min, 30 min, 1 h, 2 h, 4 h, 6 h, 8 h, 12 h, 16 h, 24 h, 36 h, 48 h, 60 h, 72 h, and 84 h post-injection. Plasma (100 μL) was separated and mixed with an equal volume (100 μL) of 1 M NaOH solution. The mixture was subjected to alkaline hydrolysis for 4 h, followed by neutralization with 100 μL of 1.4 M H₃PO₄. Subsequently, 200 μL of the resulting solution was extracted using 800 μL of acetonitrile, vortexed thoroughly, and centrifuged. The supernatant was passed through a 0.22 μm membrane filter and analyzed via LC-MS.

***In vivo biodistribution***

To evaluate the biodistribution of CA-4S_2_@ES-Cu, equimolar amounts of RhB and RhB-labeled CA-4S2@ES-Cu were intravenously injected into Hepa1-6 tumor-bearing mice. *In vivo* bioluminescence imaging was performed at specified time points (2 h, 4 h, 8 h, 12 h, and 24 h) using a multifunctional imaging system (iBox Scientia, Analytik Jena, Germany). Simultaneously, mice were sacrificed at different time points (2 h, 4 h, 8 h, 12 h, and 24 h) post-injection. Tumors and major organs were collected, and the biodistribution of CA-4S_2_@ES-Cu conjugates was assessed by measuring fluorescence intensity using the multifunctional imaging system.

***Cell lines and culture***

Huh-7 and Hepa1-6 cell lines were purchased from the Cell Bank of Type Culture Collection of the Chinese Academy of Sciences China (Shanghai, China). AML12 cell lines were purchased from Servicebio (Wuhan, China). THLE-2 cell lines were purchased from AoRuicell (Shanghai, China). Huh-7 and Hepa1-6 cells were cultured in DMEM medium, while THLE-2 and AML12 cells were cultured in THLE-2-specific medium and AML12-specific medium, respectively. All media were supplemented with 10% FBS and 1% penicillin-streptomycin. Cells were incubated at 37 ℃ in a humidified incubator containing 5% CO_2_.

***Tumor cell conditioned medium***

Huh-7 conditioned medium (CM) was prepared by culturing Huh-7 cells to approximately 80% confluency and replacing the medium with serum-free DMEM for an additional 24 h. The supernatant was collected and centrifuged at 2,500 g for 10 min at room temperature to remove cell debris. It was then filtered through a 0.22 μm membrane and stored at -80 ℃ for further experiments.

***Intracellular Cu^2+^ content***

Huh-7, Hepa1-6, THLE-2, and AML12 cells (1 × 10^6^ each) were seeded in 6-well plates and incubated overnight. Cells were then collected by trypsin digestion, lysed on ice, and the intracellular Cu^2+^ content was determined using a Cell Copper Colorimetric Assay Kit. Additionally, cells treated with ES-Cu, CA-4C_6_ NPs, CA-4S_2_ NPs, CA-4C_6_@ES-Cu, and CA-4S_2_@ES-Cu (all at 20 nM) for 48 h were subjected to the same procedure for Cu^2+^ content determination.

***Cytotoxicity assay***

Huh-7, Hepa1-6, THLE-2, and AML12 cells were seeded at a density of 2 × 10^3^ cells per well in 96-well plates and incubated overnight. The cells were treated with different concentrations of the specified drugs for 48 h. MTT (5 mg/mL) was added (20 μL/well), and the cells were incubated at 37 ℃ for 2 h. After removing the culture medium, 100 μL of DMSO was added to dissolve the formazan crystals. Absorbance values were measured at 490 nm using a SpectraMax ID5 (Molecular Devices, USA) to determine the IC_50_ values.

***Cell morphology***

1 × 10^6^ Huh-7, Hepa1-6, THLE-2, and AML12 cells were seeded in 6-well plates and incubated overnight. After treatment with ES-Cu, CA-4C_6_ NPs, CA-4S_2_ NPs, CA-4C_6_@ES-Cu, and CA-4S_2_@ES-Cu (all at 20 nM) for 12 h, cell morphology was observed using an inverted microscope.

***Cell proliferation***

An EeyoClink™ EdU cell proliferation kit containing Alexa Fluor 647 was used to evaluate cell proliferation ability. 5 × 10^5^ HCC cells were seeded in 12-well plates containing coverslips and incubated overnight. The cells were treated with ES-Cu, CA-4C_6_ NPs, CA-4S_2_ NPs, CA-4C_6_@ES-Cu, and CA-4S_2_@ES-Cu (all at 20 nM) for 24 h. The cells were then incubated with EdU for 2 h, fixed with 4% paraformaldehyde, and permeabilized with 0.3% Triton X-100. Afterward, the cells were incubated with the click reaction mixture in the dark at room temperature for 30 min. Following labeling, the cell nuclei were stained with DAPI. Experimental results were visualized using a confocal laser scanning microscope.

***Colony formation***

We implemented colony formation experiments to investigate the colony-forming ability. After overnight cell adherence, various drugs were added to the wells for 48 h. Then, cells were incubated in a serum-free medium for 14 days and fixed in 4% polyformaldehyde, stained with 0.1% crystal violet solution. The total number of colonies was observed under microscopy.

***Microtubule morphology***

Microtubule morphology was assessed using the Tubulin-Tracker Green Staining Kit. 1 × 10^6^ HCC cells were seeded in 6-well plates and incubated overnight. After treatment with ES-Cu, CA-4C_6_ NPs, CA-4S_2_ NPs, CA-4C_6_@ES-Cu, and CA-4S_2_@ES-Cu (all at 20 nM) for 12 h, the cells were incubated with Tubulin-Tracker for 30 min. Microtubule morphology was observed using a confocal microscope.

***Cell cycle analysis***

Cell cycle distribution and DNA content were analyzed by PI staining. 1 × 10^6^ HCC cells were seeded in 6-well plates and incubated overnight. After exposure to ES-Cu, CA-4C_6_ NPs, CA-4S_2_ NPs, CA-4C_6_@ES-Cu, and CA-4S_2_@ES-Cu (all at 10 nM) for 24 h, the cells were collected by trypsinization and fixed overnight in 70% ethanol at 4 ℃. The fixed cells were centrifuged and incubated with a staining buffer containing PI and RNase. Cell cycle progression was measured by flow cytometry.

***Apoptosis detection***

Apoptosis detection was performed using an Annexin V-FITC Apoptosis Assay Kit. 1 × 10^6^ HCC cells were seeded in a 6-well plate and incubated overnight. After 48 h of treatment with ES-Cu, CA-4C_6_ NPs, CA-4S_2_ NPs, CA-4C_6_@ES-Cu, and CA-4S_2_@ES-Cu (all at 20 nM), the cells were stained with Annexin V, FITC, and PI at room temperature for 15 min. Subsequently, apoptosis was analyzed by flow cytometry.

***Intracellular Cu⁺ content***

1 × 10^6^ HCC cells were seeded in a 6-well plate and incubated overnight. After 48 h of treatment with ES-Cu, CA-4C_6_ NPs, CA-4S_2_ NPs, CA-4C_6_@ES-Cu, and CA-4S_2_@ES-Cu (all at 20 nM), the cells were harvested by trypsin digestion. After cell disruption on ice, the intracellular Cu⁺ content was measured using the Cellular Cuprous Fluorometric Assay Kit.

***Cell immunofluorescence***

5 × 10^5^ HCC cells were seeded in 12-well plates containing coverslips and incubated overnight. The cells were treated with ES-Cu, CA-4C_6_ NPs, CA-4S_2_ NPs, CA-4C_6_@ES-Cu, and CA-4S_2_@ES-Cu (all at 20 nM) for 24 h. The cells were fixed with 4% paraformaldehyde for 30 min. After fixation, the cells were incubated overnight at 4 ℃ with DLAT/HMGB1/CRT primary antibodies (dilution 1:500), followed by incubation with Alexa Fluor 647-conjugated goat anti-rabbit secondary antibody (dilution 1:500) at room temperature for 2 h. The cells were then stained with DAPI for 5 min at room temperature. The results were observed using a confocal laser scanning microscope or flow cytometry.

***GSH detection***

The Total Glutathione Assay Kit was used to measure the GSH levels in HCC cells. 1 × 10^6^ HCC cells were seeded in 6-well plates and incubated overnight. After 24 h of treatment with ES-Cu, CA-4C_6_ NPs, CA-4S_2_ NPs, CA-4C_6_@ES-Cu, and CA-4S_2_@ES-Cu (all at 20 nM), the cells were harvested by trypsin digestion. The samples were subjected to two rapid freeze-thaw cycles using liquid nitrogen and a 37 ℃ water bath. After centrifugation at 10,000g for 10 min at 4 ℃, the supernatants were collected for the total glutathione assay.

***Western blot***

HCC cells treated with ES-Cu, CA-4C_6_ NPs, CA-4S_2_ NPs, CA-4C_6_@ES-Cu, and CA-4S_2_@ES-Cu (all at 20 nM) for 24 h were washed with cold PBS and collected using RIPA buffer containing a cocktail of protease inhibitors and phenylmethanesulfonyl fluoride. The lysates were centrifuged by high-speed and low-temperature centrifugation. Protein samples were separated by 12.5%, 10%, or 7.5% SDS-PAGE and transferred to polyvinylidene fluoride membranes. The membranes were blocked with 5% nonfat dry milk for 2 h at room temperature and then incubated overnight at 4 ℃ with the corresponding primary antibodies in Tris-buffered saline with Tween-20 (TBST). The membranes were washed three times with TBST buffer and incubated with appropriate horseradish peroxidase-conjugated secondary antibodies at room temperature for 2 h. Finally, the protein bands were detected using an ECL kit, and the results were quantified using ImageJ software.

***ROS detection***

The Reactive Oxygen Species Assay Kit was used to evaluate ROS generation. HCC cells were treated with ES-Cu, CA-4C_6_ NPs, CA-4S_2_ NPs, CA-4C_6_@ES-Cu, and CA-4S_2_@ES-Cu (all at 20 nM) for 24 h. After labeling, the cells were incubated with a serum-free medium containing DCFH-DA solution (10 μM) at 37 ℃ for 30 min in the dark. Following two washes with PBS, excess DCFH-DA was removed. The intensity of DCF fluorescence was detected by flow cytometry.

***Mitochondrial membrane potential detection***

Mitochondrial membrane potential was assessed using the Enhanced Mitochondrial Membrane Assay Kit with JC-1. HCC cells were treated with ES-Cu, CA-4C_6_ NPs, CA-4S_2_ NPs, CA-4C_6_@ES-Cu, and CA-4S_2_@ES-Cu (all at 20 nM) for 48 h. The cells were then washed twice with PBS, trypsinized, suspended in JC-1, and incubated in the dark for 20 min at 37 ℃ followed by two washes with the JC-1 working solution and suspension in a basic medium. The final results were detected using a laser confocal microscope.

***ATP detection***

Extracellular ATP levels were measured using an ATP Assay Kit. A total of 1 × 10^6^HCC cells were seeded in a 6-well plate and incubated overnight. After treatment with ES-Cu, CA-4C_6_ NPs, CA-4S_2_ NPs, CA-4C_6_@ES-Cu, and CA-4S_2_@ES-Cu (all at 20 nM) for 48 h, the cell supernatants were collected and incubated with the ATP detection working solution for 10 min. ATP levels were calculated from the luminescence signals using a multi-function measuring instrument (FlexStation 3).

***Immune cells and cytokines analysis***

Immune cell proportions were measured using a cocktail antibody method. Tumor tissues were obtained and digested into a single-cell suspension. The cell suspension was added to the flow cytometry staining buffer (FCSB). The cells were blocked with Mice TruStain FcX (Fc Receptor Blocking Solution, BioLegend) on ice for 10 min. They were then stained with the following anti-mouse antibodies: FVS780, CD45-Pacific Blue, CD11b-PE-CY7, F4/80-FITC, CD86-PE, CD206-APC, CD11c-FITC, CD80-APC, CD3-PE, CD4-FITC, CD49b-FITC, Foxp3-APC, and CD8-APC for 30 min. The final results were detected by flow cytometry. FACS data were analyzed using FlowJo 10 flow cytometry analysis software (FlowJo LLC). For cytokine measurement, tumor tissues were homogenized, and the supernatant was collected to measure IFN-𝛾 levels using the Mouse IFN-γ (Interferon Gamma) ELISA Kit.

***Wound healing assay***

HUVECs at the logarithmic growth phase were suspended in DMEM and seeded at a density of 40,000 cells per well in 6-well plates. Cells were cultured until they reached approximately 90% confluence. A scratch was made using a 10 μL pipette tip, followed by two washes with sterile PBS. Drug-containing or drug-free culture medium was added and incubated for 24 h. The healing process was observed at 0 and 24 h using an inverted microscope, and images of the wound area were captured. The healing area was analyzed and calculated using ImageJ software. The wound healing rate was calculated as follows:

Wound healing rate = [(0 h wound area - 24 h wound area) / 0 h wound area] × 100%.

***Cell migration and invasion assay***

DMEM containing 10% FBS was added to 24-well plates, and sterile Transwell inserts were placed into each well. HUVECs at the logarithmic growth phase were resuspended in a drug-containing or drug-free culture medium and seeded into the upper chambers of the Transwell inserts at a density of 40,000 cells per well. After 24 h, cells were fixed with 4% paraformaldehyde for 15 min, followed by staining with 0.1% crystal violet for 15 min. After removing excess crystal violet from the upper membrane, cells that migrated to the lower membrane were observed and imaged using an inverted microscope. For the invasion assay, the upper membrane of the Transwell insert was coated with Matrigel, and the other experimental procedures were the same as described above.

***Tubule formation assay***

Fifty microliters of Matrigel was added to each well of a 96-well plate and allowed to solidify at 37 ℃ for 30 min. HUVECs at the logarithmic growth phase were resuspended in a drug-containing or drug-free culture medium and seeded at a density of 40,000 cells per well on top of the Matrigel. After 4–5 h of incubation, the tube formation was observed and photographed using an inverted microscope. The images were analyzed using ImageJ software.

***Rat aortic ring assay***

Male rats weighing 180-200 g were deeply anesthetized with 4% chloral hydrate and euthanized. The aorta was carefully isolated, and the surrounding fat was removed. The aorta was then digested with 0.2% type II collagenase for 15 min. After further cleaning the surrounding tissues, the aorta was cut into 1-2 mm rings and placed individually into a 96-well plate pre-coated with Matrigel. Each aortic ring was embedded in 50 μL of Matrigel and incubated at 37 ℃ for 30 min to solidify. The rings were then cultured in DMEM containing 10% FBS for 3 days, with the medium changed every other day. After this, the rings were incubated with either a drug-containing or drug-free culture medium until day 10. The aortic sprouting was observed and photographed using an inverted microscope.

***In* *vivo* *antitumor activity***

All animal procedures were approved by the Experimental Animals Administrative Committee of Chengdu University of Traditional Chinese Medicine (Registration number: SYXK-2020-124, Chengdu, China) and were conducted under relevant animal regulations. C57BL/6J mice (6-8 weeks old, 18-20 g) were provided by Beijing SiPeiFu Biotechnology Co., Ltd. (Beijing, China). The HCC mouse model was established by injecting 1 × 10^6^ Hepa1-6 cells into the left flank of C57BL/6J mice. After one week, the mice were randomly divided into six groups, with 5 mice per group, and treated with normal saline, ES-Cu, CA-4C_6_ NPs, CA-4S_2_ NPs, CA-4C_6_@ES-Cu, and CA-4S_2_@ES-Cu (all at 5 mg/kg, based on CA-4 content) via tail vein injection, once every two days. Upon completion of the experiment, all mice were euthanized, and tumor volume, weight, and organ weights were measured. Blood samples were collected for serum biochemical analysis. Tumor tissues were fixed in formalin, embedded in paraffin, and sectioned. Hematoxylin and eosin (H&E) staining, as well as immunohistochemical staining for Ki67, DLAT, MMP-14, and VEGFA, were performed, along with immunofluorescence staining for CD4 and CD8.

***Establishment of the liver cancer lung metastasis model and evaluation of drug efficacy***

1 × 10^6^ Hepa1-6 cells were injected via the tail vein into C57BL/6 mice. One week later, the mice were divided into 6 groups, with 5 mice per group. Mice were administered 4 doses (on days 0, 3, 6, and 9) of the following treatments via intravenous injection: ES-Cu, CA-4C_6_ NPs, CA-4S_2_ NPs, CA-4C_6_@ES-Cu, and CA-4S_2_@ES-Cu (all at 5 mg/kg, based on CA-4 content). On day 12 post-treatment, lung tissues were harvested, and H&E staining were performed as described previously.

***Statistical analysis***

All data were expressed as the mean ± standard deviation (SD) of three independent experiments. Differences between groups were assessed using one-way analysis of variance (ANOVA), followed by the Bonferroni post-test for pairwise comparison. Statistical significance between the control and test groups was evaluated using GraphPad Prism 9 for correlation analysis. Sample size (n ≥ 3) for each statistical analysis. The significance levels were defined as follows: * *p* < 0.05, ** *p* < 0.01, *** *p* < 0.001, compared with the specified group.

**Supplementary figures (S1-S10)**


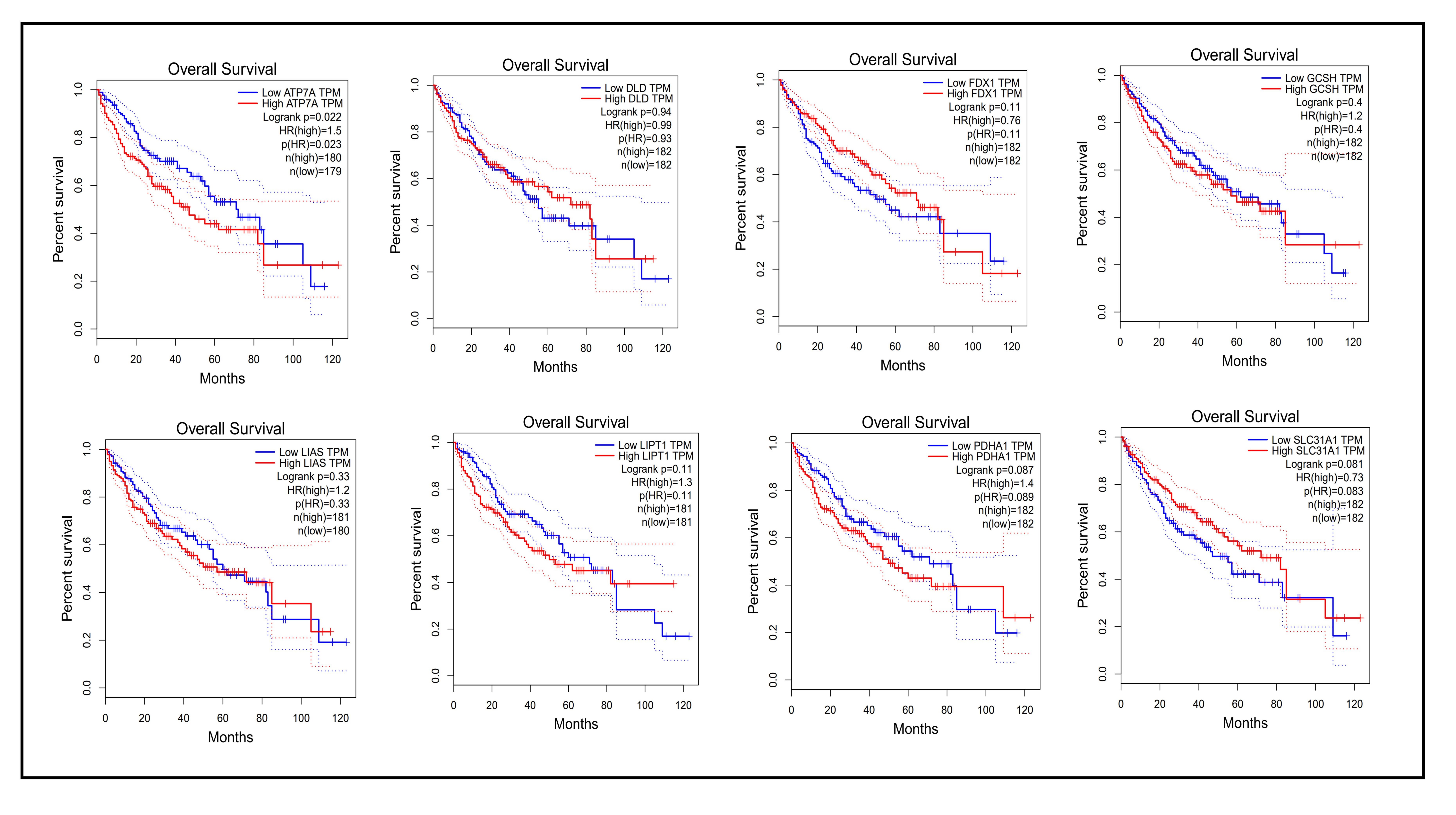


**Fig. S1** Kaplan-Meier survival analysis of the gene signature from TCGA dataset. Log-rank test was used to compare different groups. (HR represented the hazard ratio of the low-expression sample relatives to the high-expression sample. HR > 1 indicated the gene was a risk factor, and HR < 1 indicated the gene was a protective factor).


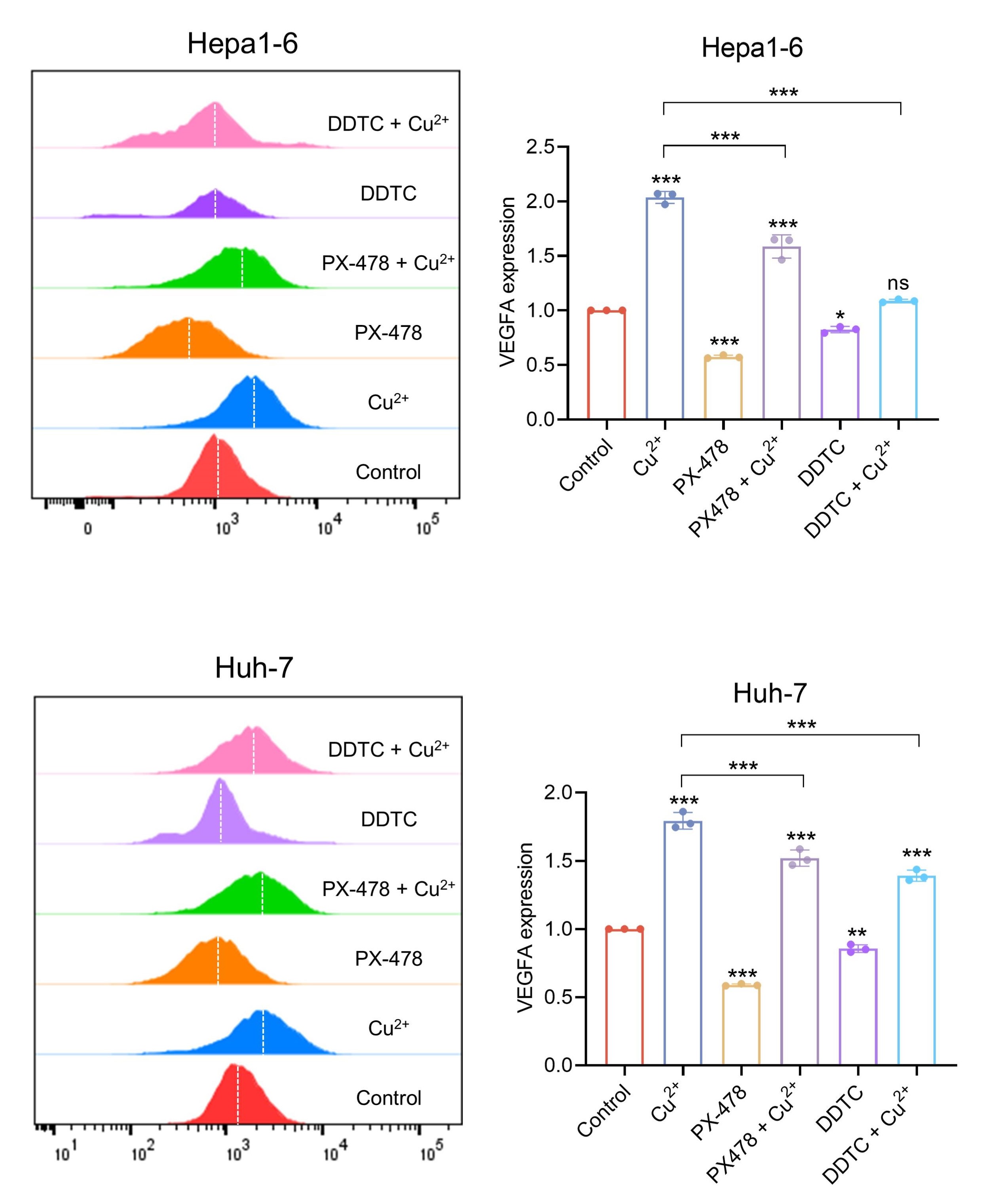

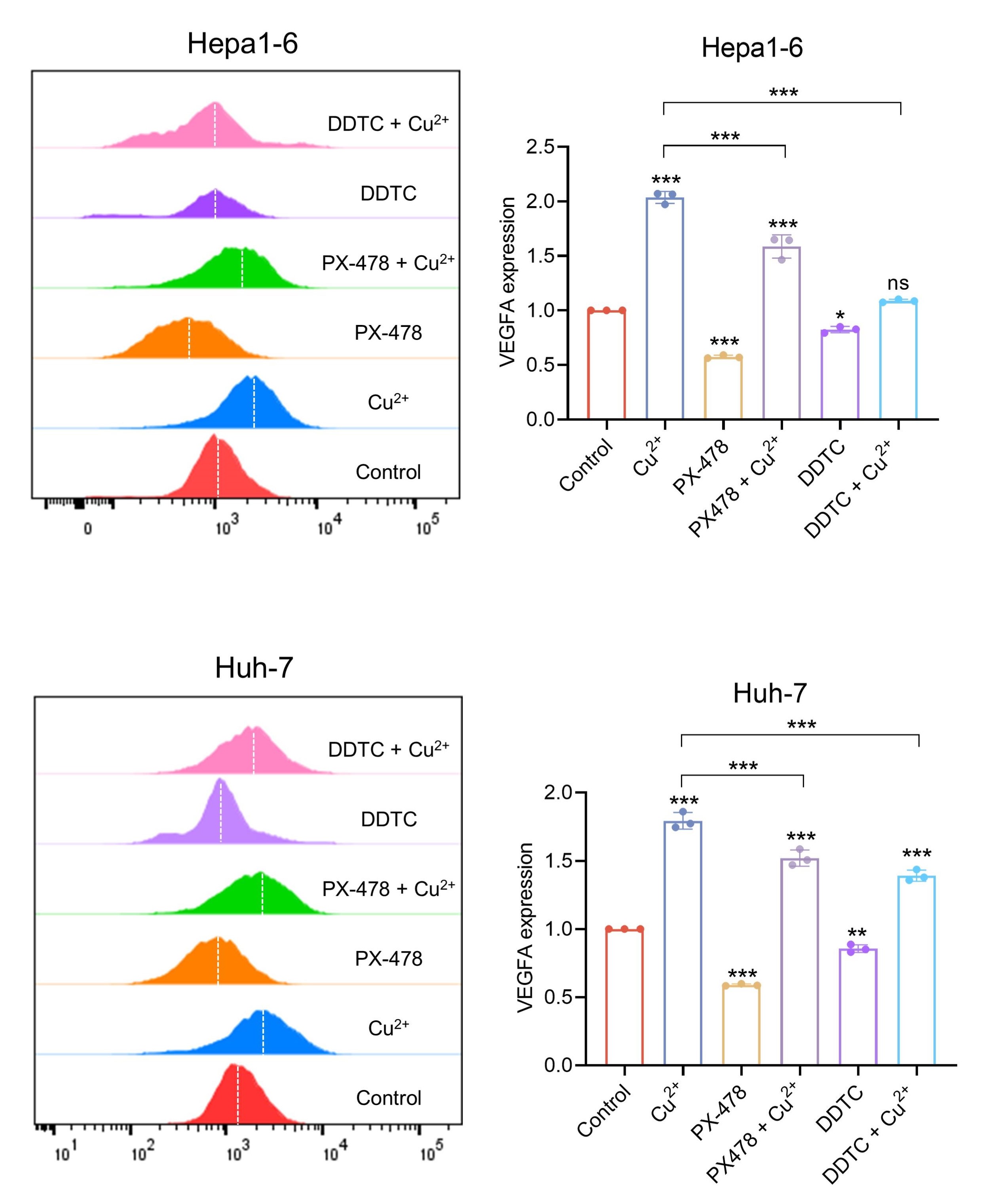


**Fig. S2** Flow cytometric analysis of VEGFA expression in HCC cells following distinct treatments. * *p* < 0.05, ** *p* < 0.01, *** *p* < 0.001, vs. the specified group.

**
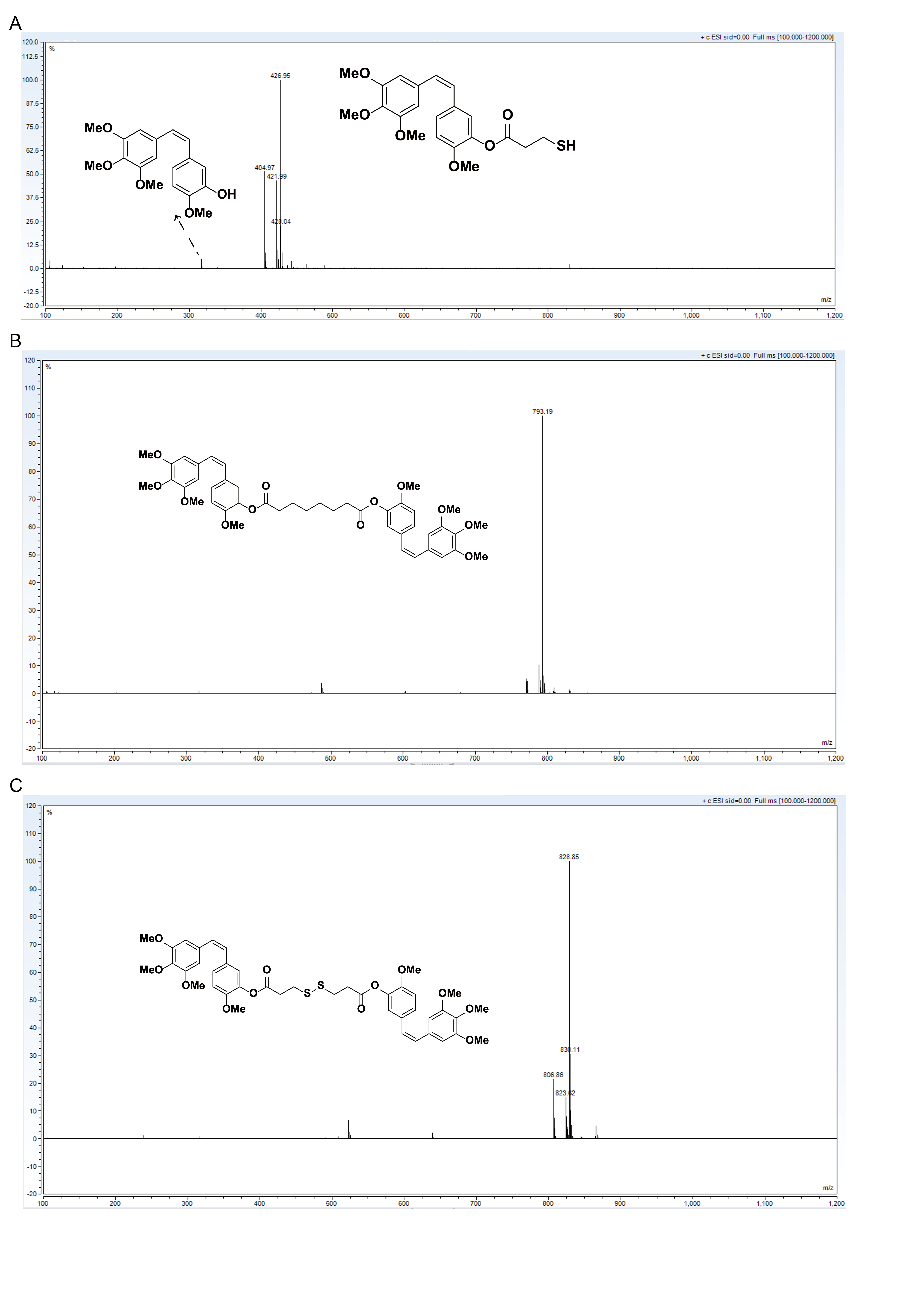
**

**Fig. S3** MS detecting drug release triggered by GSH. **(A)** Detection of CA-4S_2_ solution by MS after 4 h treatment with 10 mM GSH. **(B)** Detection of CA-4C_6_ solution by MS after 4 h treatment with 10 mM GSH. **(C)** Detection of CA-4S_2_ solution by MS after 4 h treatment without GSH.


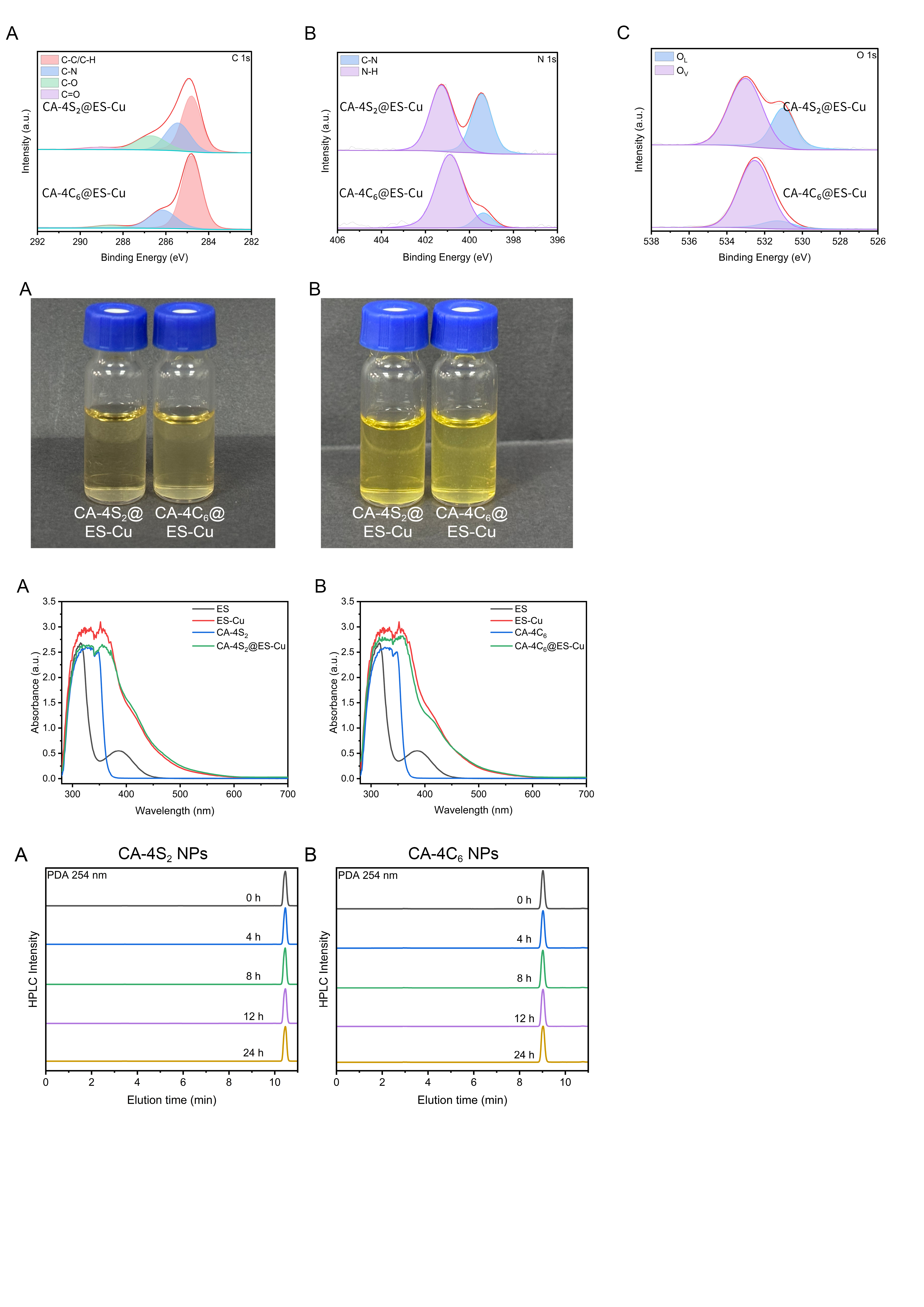


**Fig. S4** HPLC detecting drug release triggered by GSH. **(A)** The degradation of CA-4S_2_ NPs without GSH monitored by HPLC. **(B)** The degradation of CA-4C_6_ NPs without GSH monitored by HPLC.

**
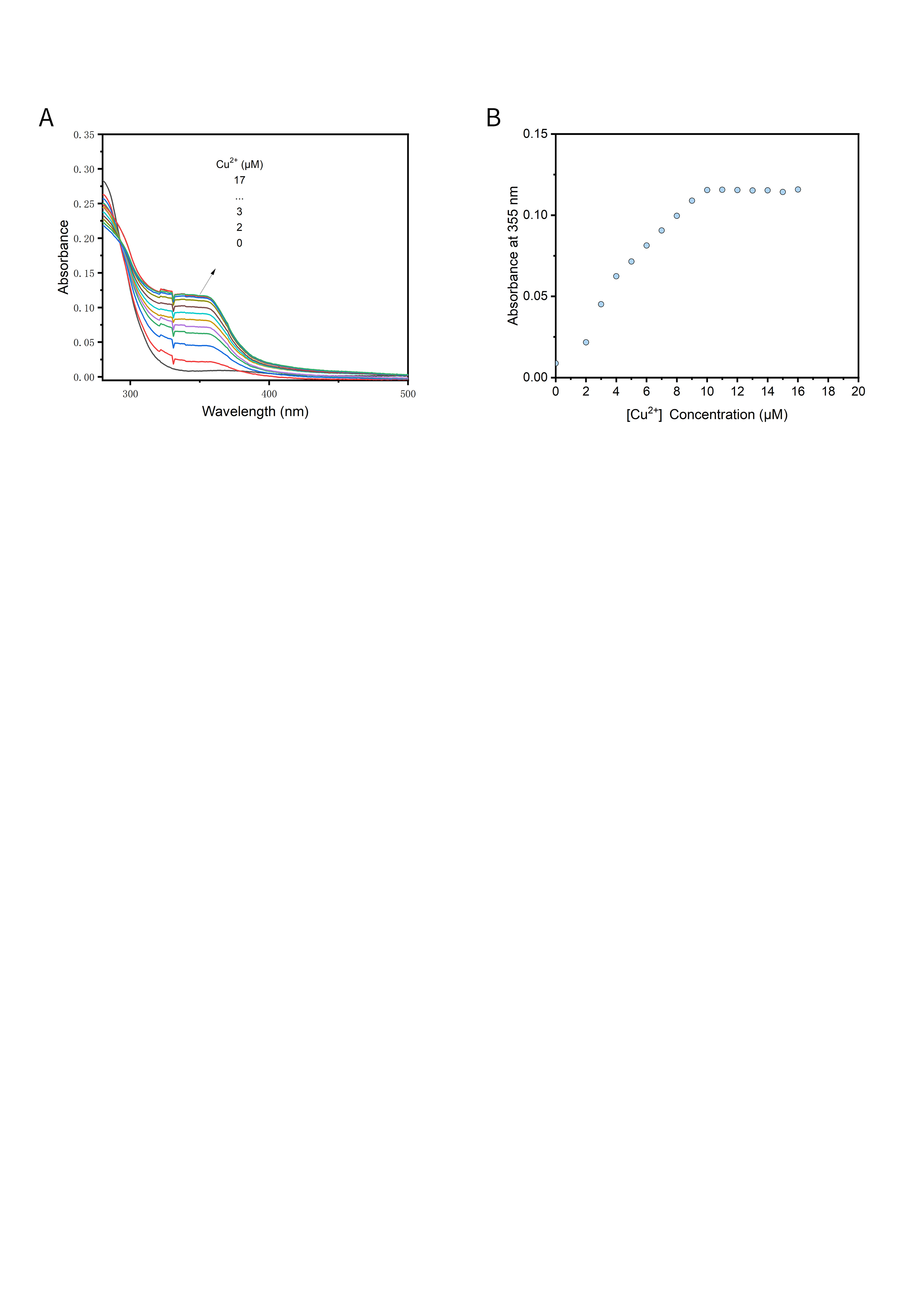
**

**Fig. S5** Titrimetric analysis of ES by spectrophotometry employing Cu²⁺**. (A)** Absorbance variation curves of ES after coordination with Cu²⁺. The arrow indicated the absorption intensity at the 355 nm shoulder. **(B****)** Absorption value trends of ES on Cu²⁺ at 355 nm.


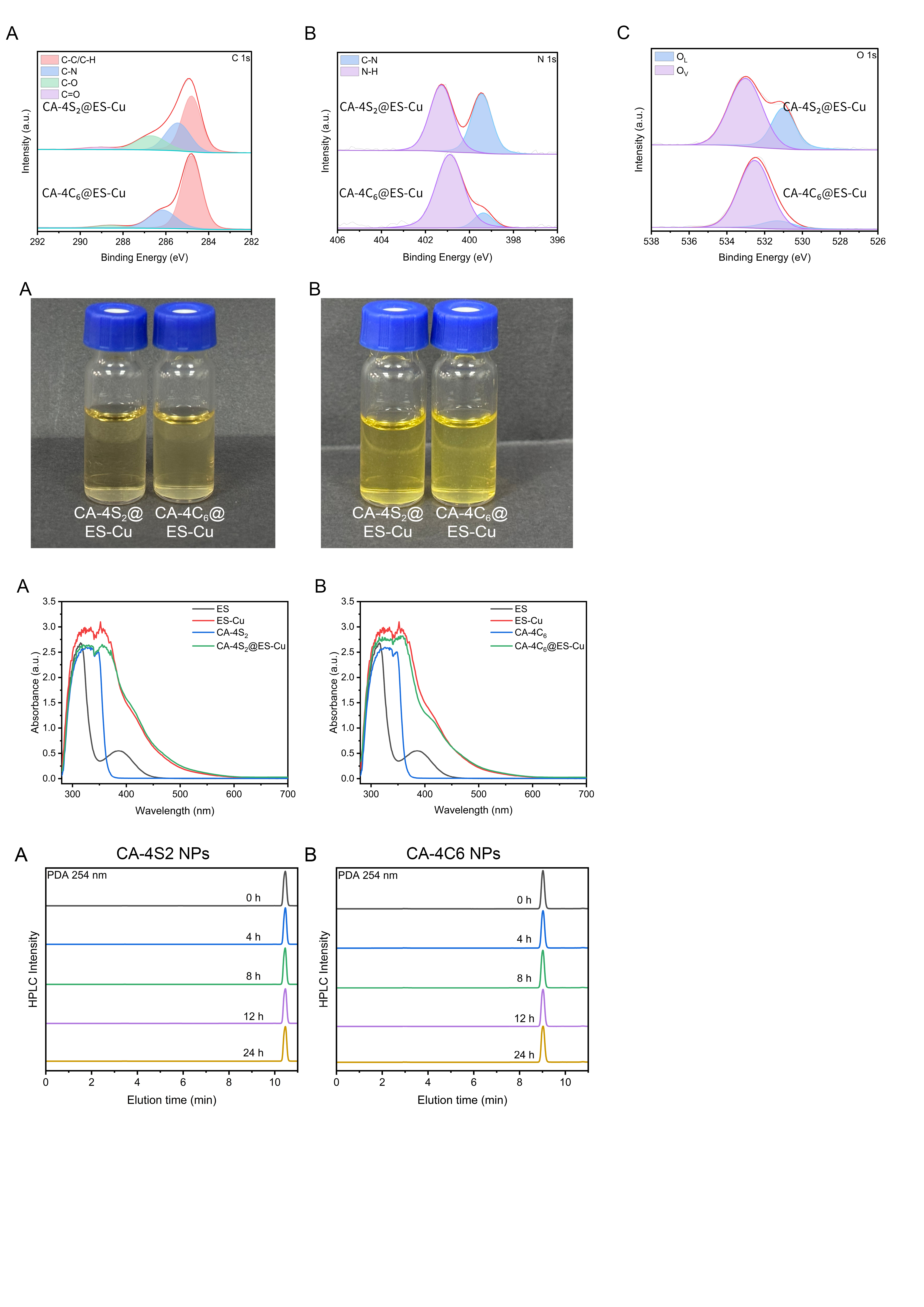


**Fig. S6** The UV-vis absorption spectra of drugs. **(A)** The UV-vis absorption spectra of ES (in DMSO), ES-Cu (in CH_2_Cl_2_), CA-4S_2_ (in CH_2_Cl_2_), and CA-4S_2_@ES-Cu(in ultrapure water). **(B)** The UV-vis absorption spectra of ES (in DMSO), ES-Cu (in CH_2_Cl_2_), CA-4C_6_ (in CH_2_Cl_2_), and CA-4C_6_@ES-Cu (in ultrapure water).

**
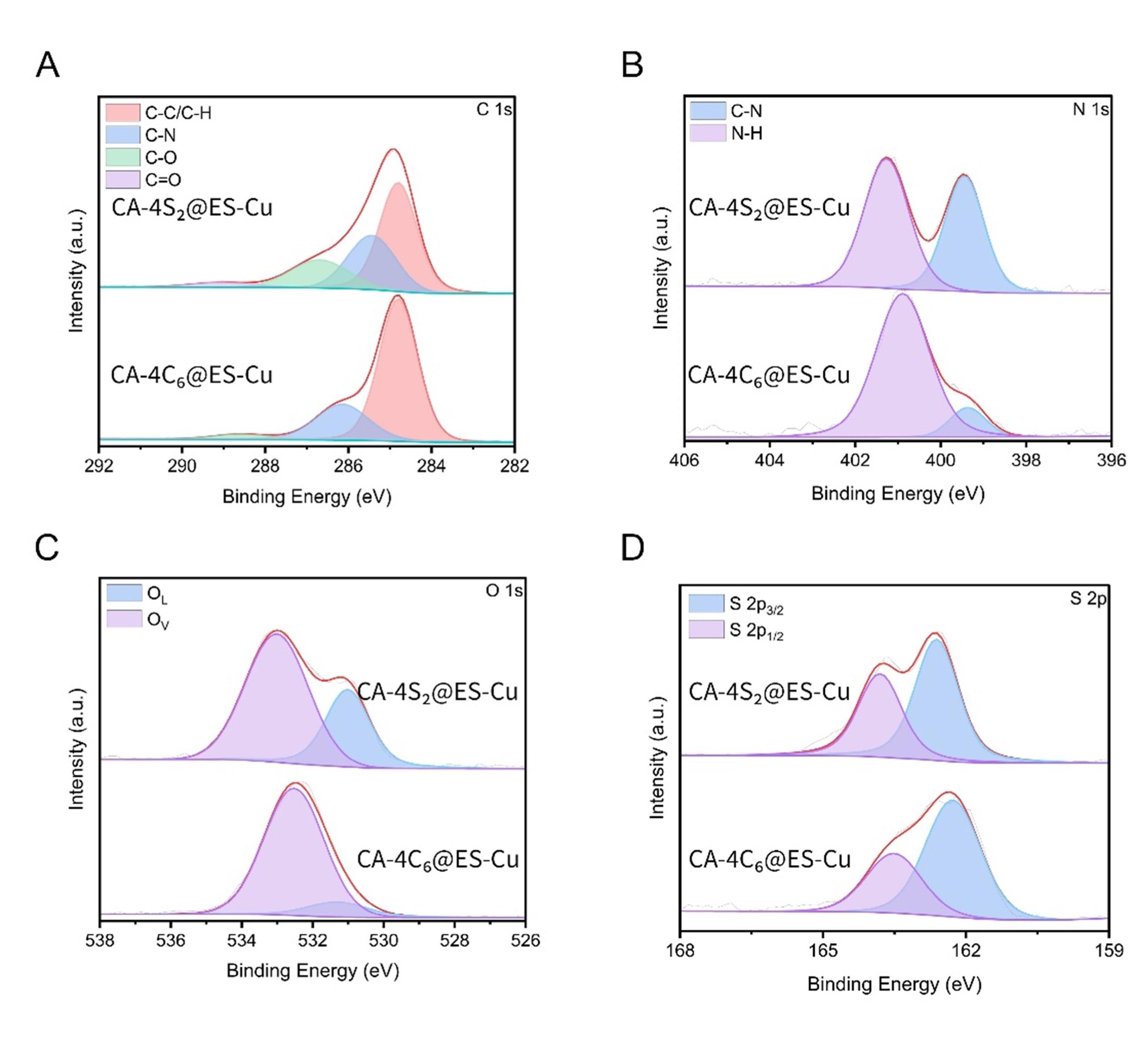
**

**Fig. S7** The XPS spectra of CA-4S_2_@ES-Cu and CA-4C_6_@ES-Cu. **(A**) High-resolution C 1s XPS spectra. **(B)** High-resolution N 1s XPS spectra. **(C)** High-resolution O 1s XPS spectra. **(D)** High-resolution S 2p XPS spectra.


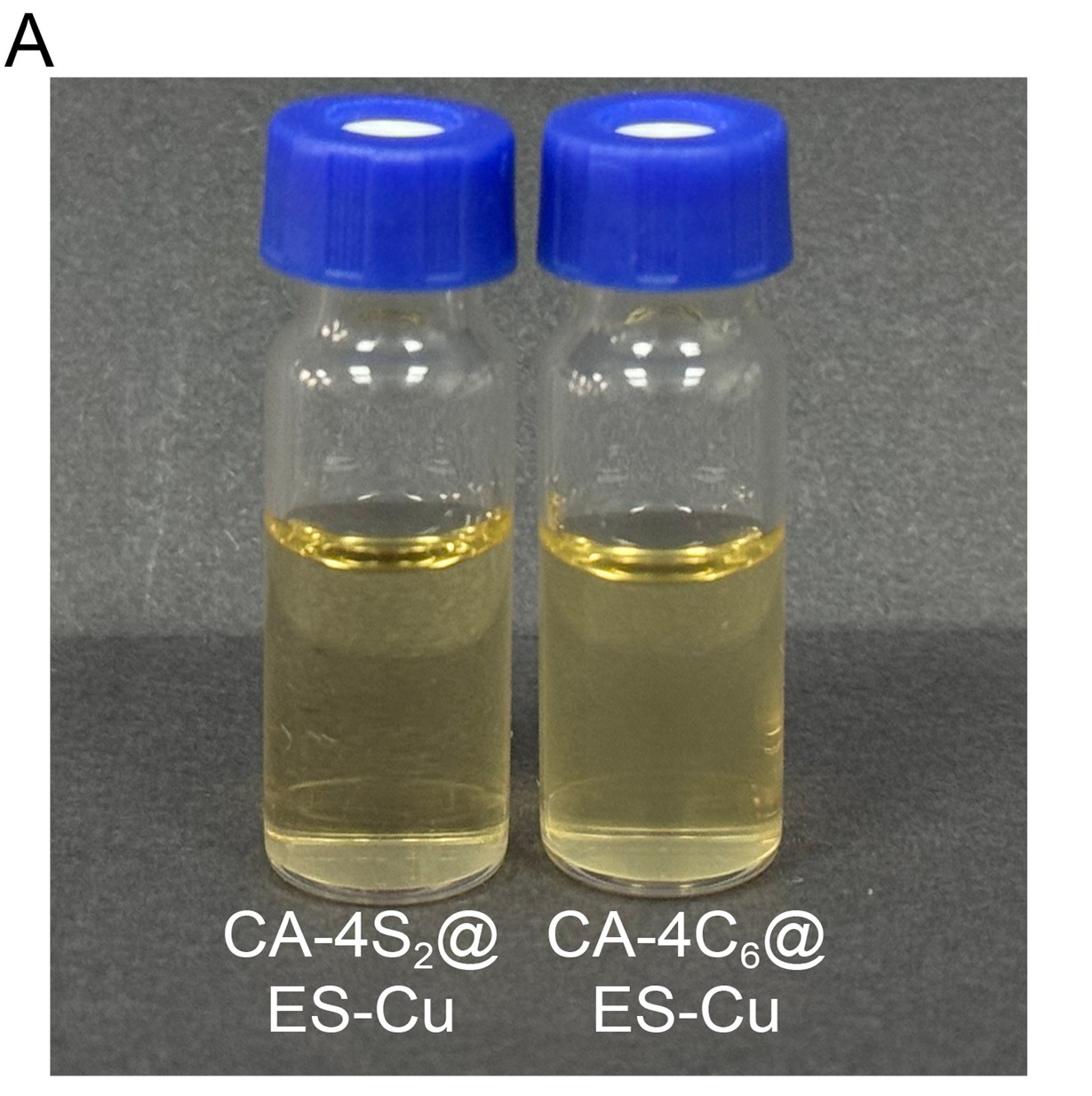


**Fig. S8** The Appearance of prepared nanoparticles. **(A)** The appearance of freshly prepared CA-4S_2_@ES-Cu and CA-4C_6_@ES-Cu (0.5 mg/mL) stored at 4 °C for 1 d.


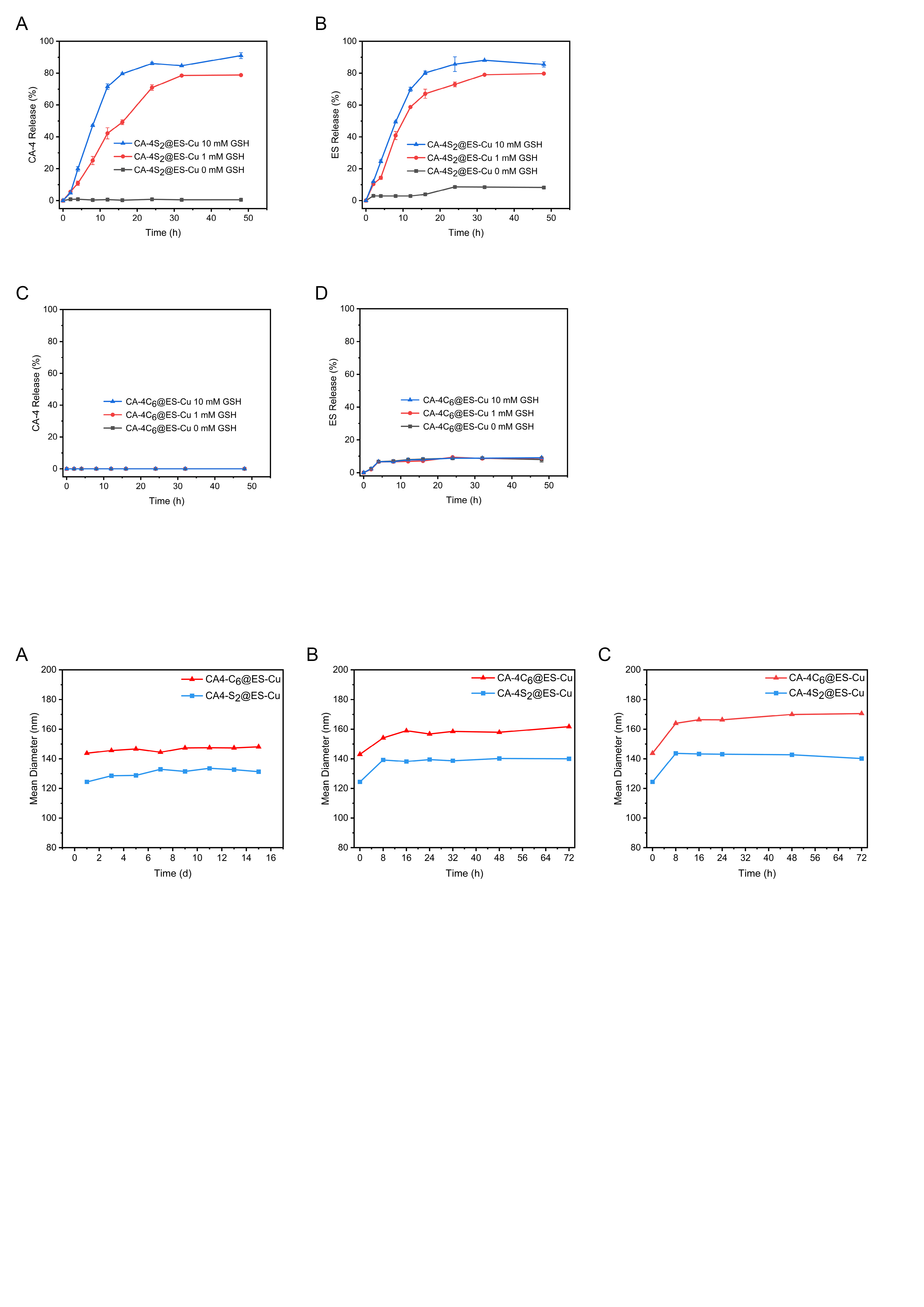


**Fig. S9** The Stability of nanoparticles. **(A)** Size changes of CA-4S_2_@ES-Cu and CA-4C_6_@ES-Cu in PBS (pH 7.4). **(B)** Size changes of CA-4S_2_@ES-Cu and CA-4C_6_@ES-Cu in PBS (pH 7.4) solution containing with 10 % FBS. **(C)** Size changes of CA-4S_2_@ES-Cu and CA-4C_6_@ES-Cu in PBS (pH 7.4) solution containing with 10 % rat plasma/heparin.


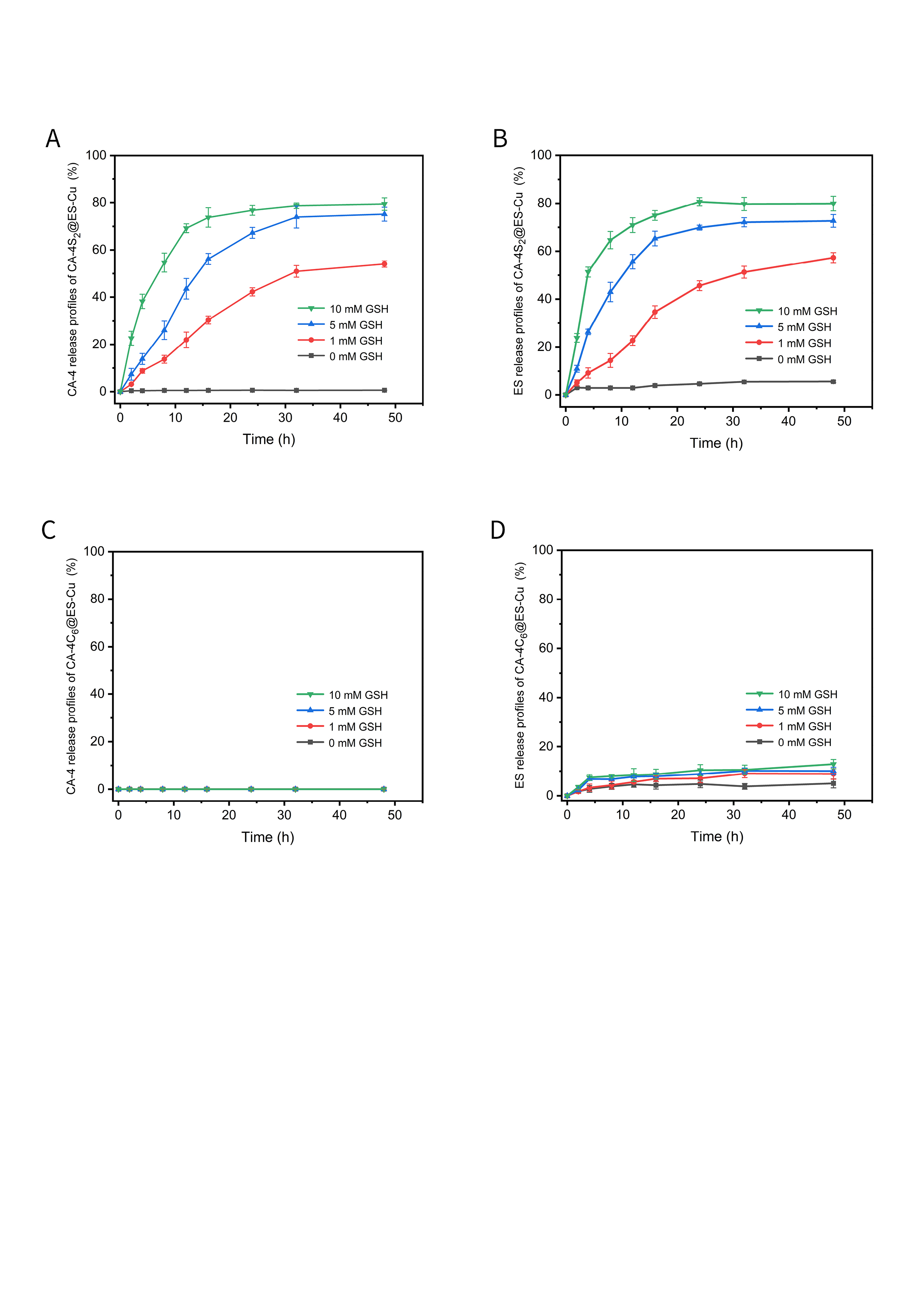


**Fig. S10** Glutathione effect on nanoparticles and the active drug release. **(A, B)** Release profiles for CA-4 or ES from CA-4S_2_@ES-Cu treated with varied concentration of GSH, respectively. **(C, D)** Release profiles for CA-4 or ES from CA-4C_6_@ES-Cu treated with varied concentration of GSH, respectively.

**^1^H NMR, ^13^C NMR analysis of synthesized compounds**

**A**


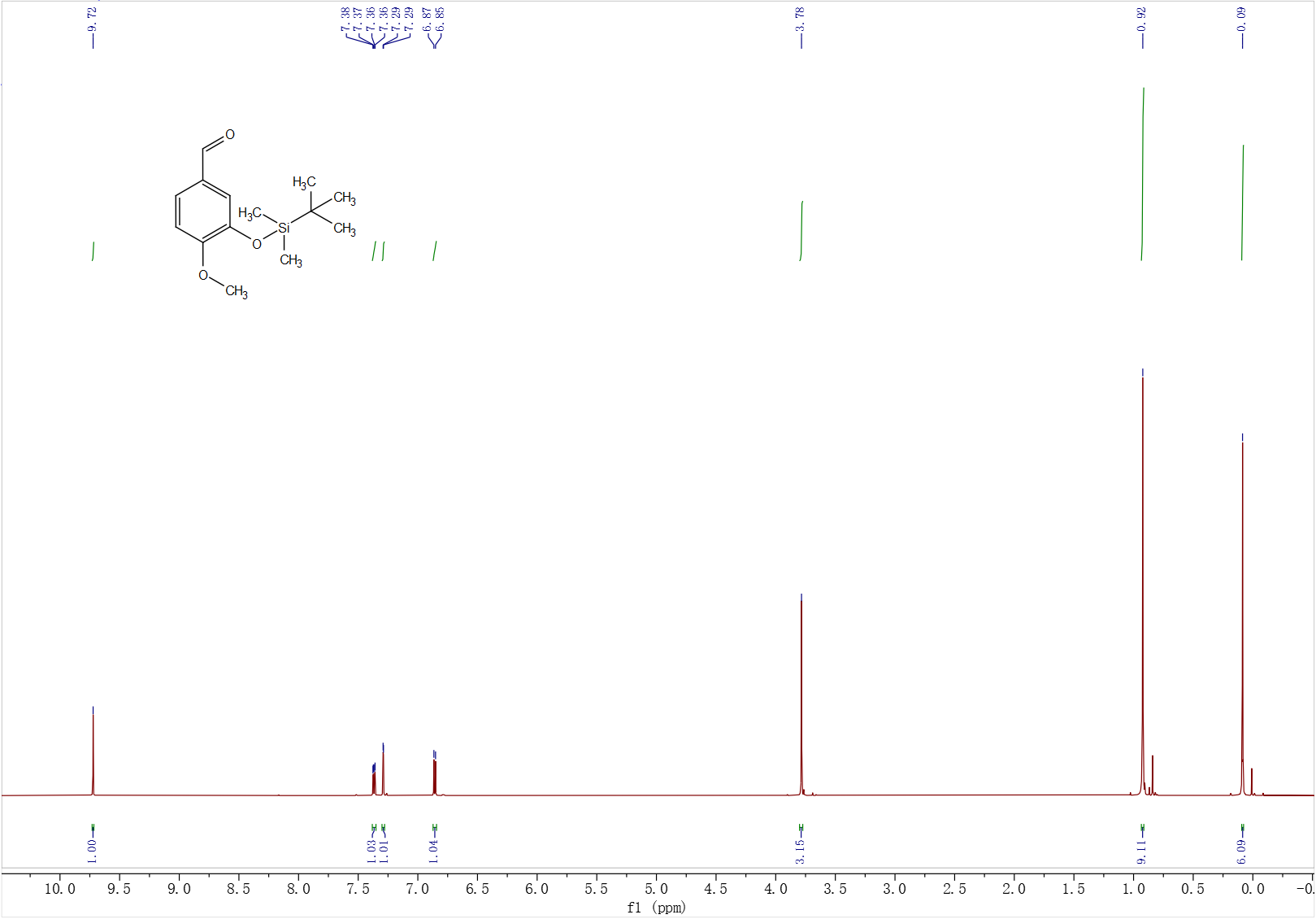


**B**


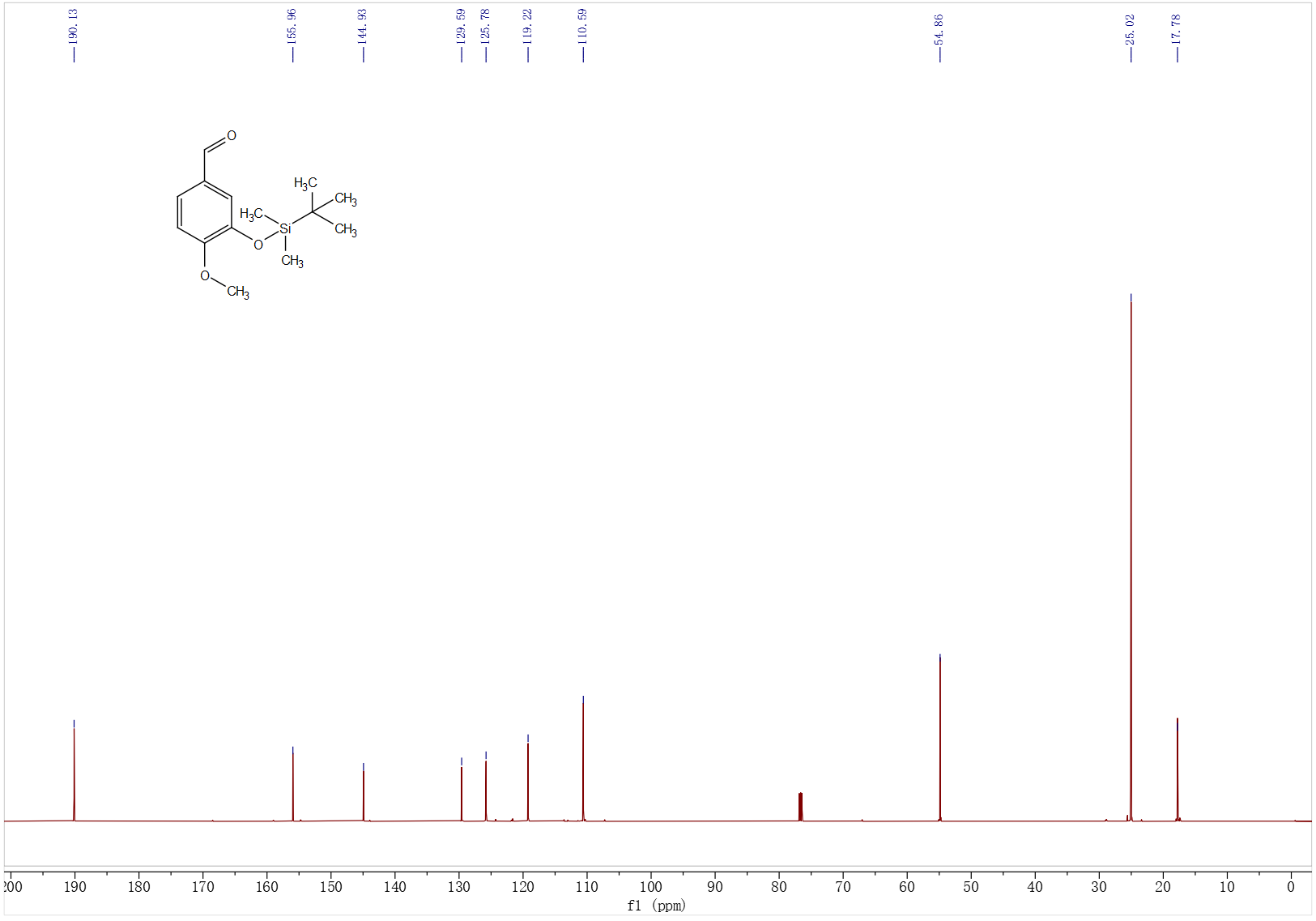


**Fig. S11 (A)** ^1^H-NMR and **(B)** ^13^C-NMR analysis of compound 3.

**A**

**
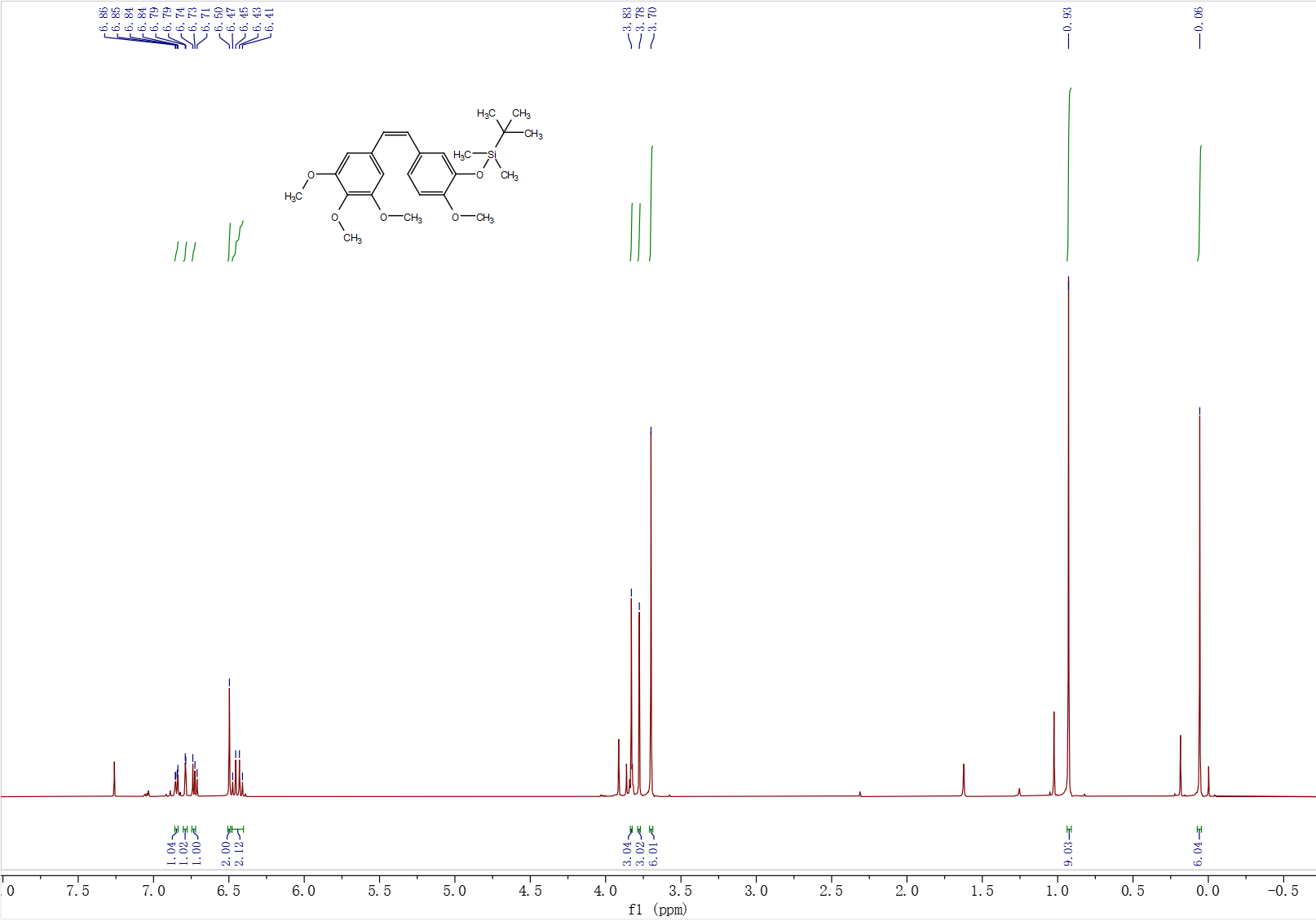
**

**B**

**
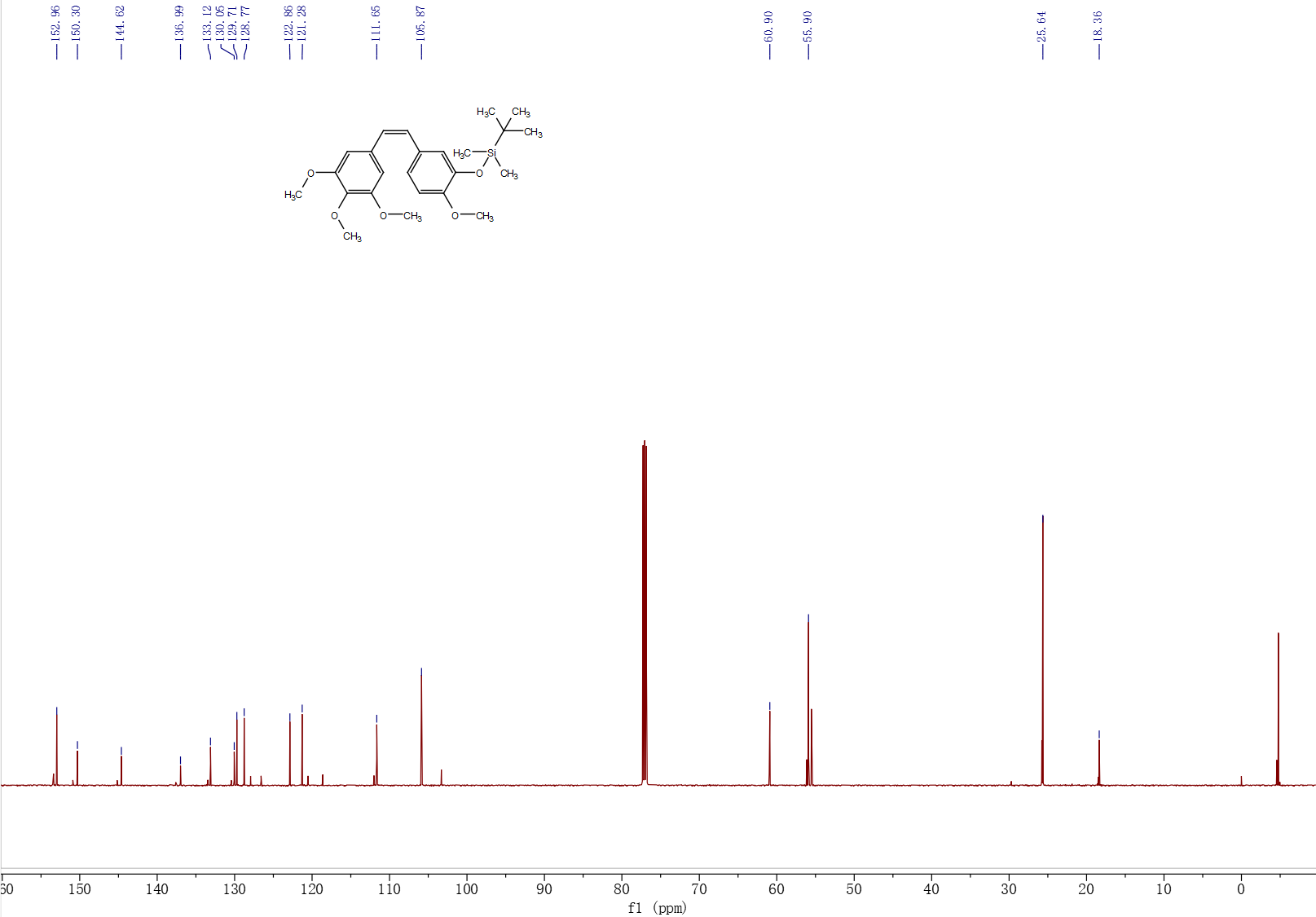
**

**Fig. S12 (A)** ^1^H-NMR and **(B)** ^13^C-NMR analysis of compound 4.

**A**

**
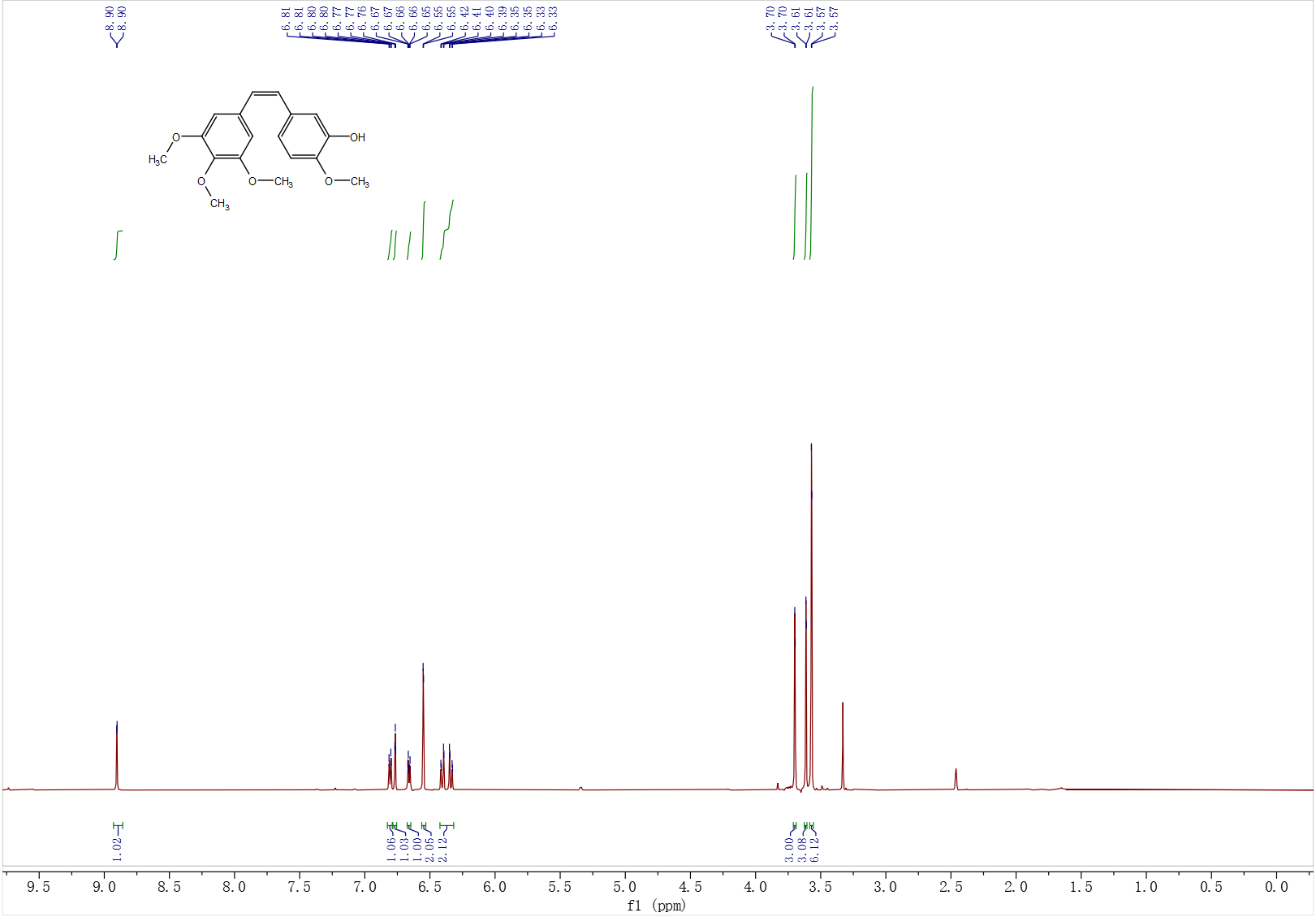
**

**B**

**
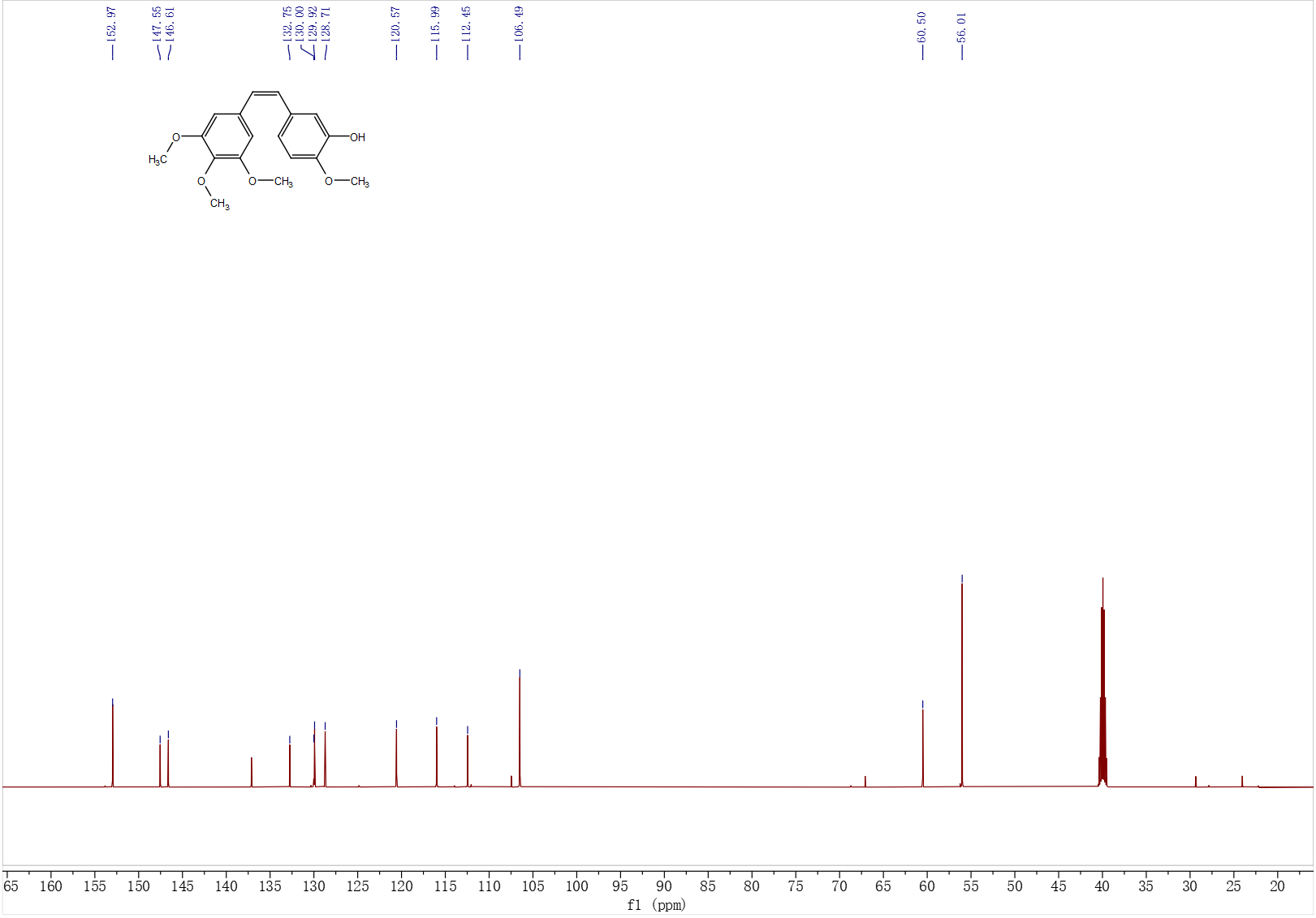
**

**Fig. S13 (A)** ^1^H-NMR and **(B)** ^13^C-NMR analysis of CA-4.

**A**

**
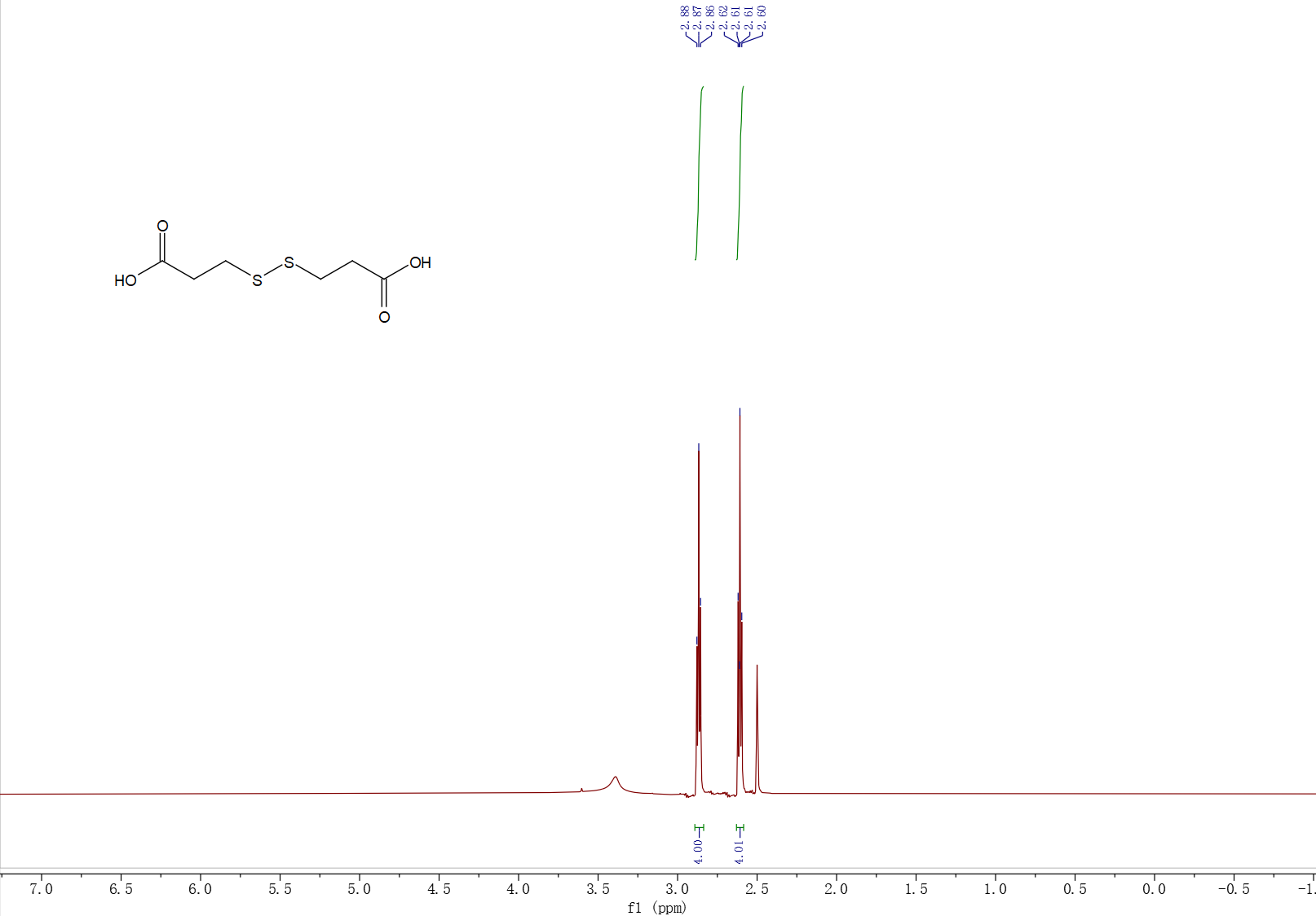
**

**B**

**
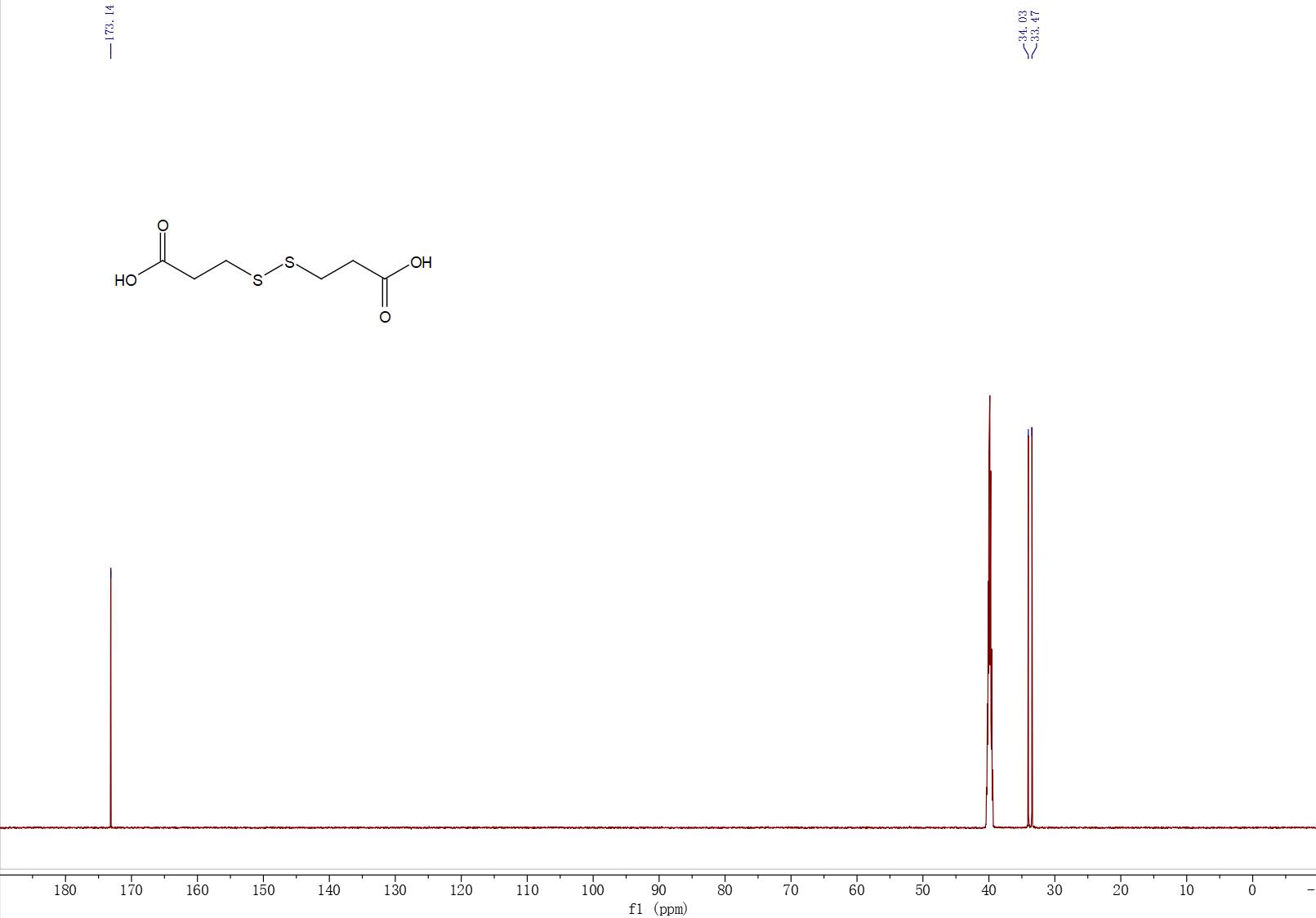
**

**Fig. S14 (A)** ^1^H-NMR and **(B)** ^13^C-NMR analysis of 5.

**A**

**
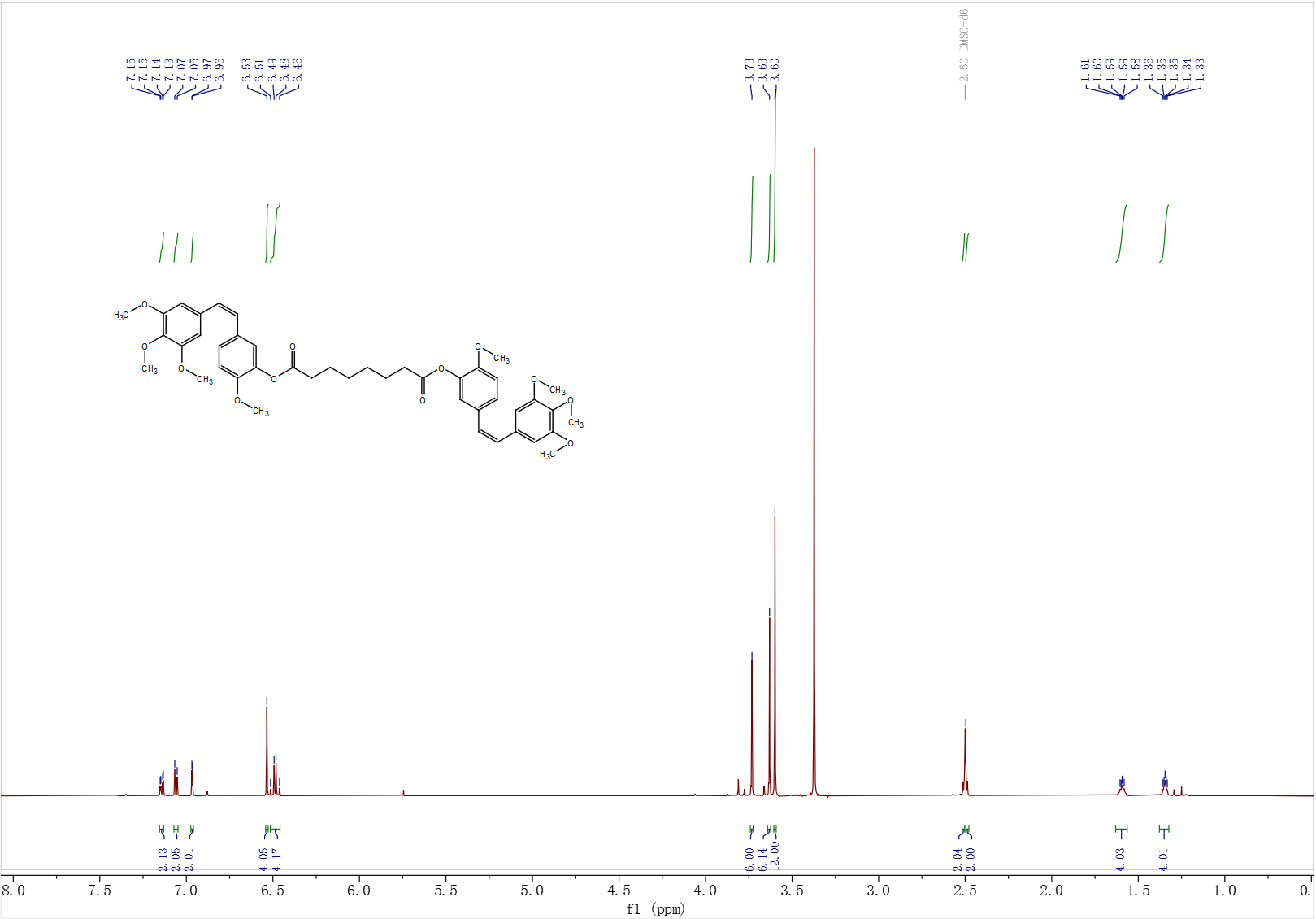
**

**B**

**
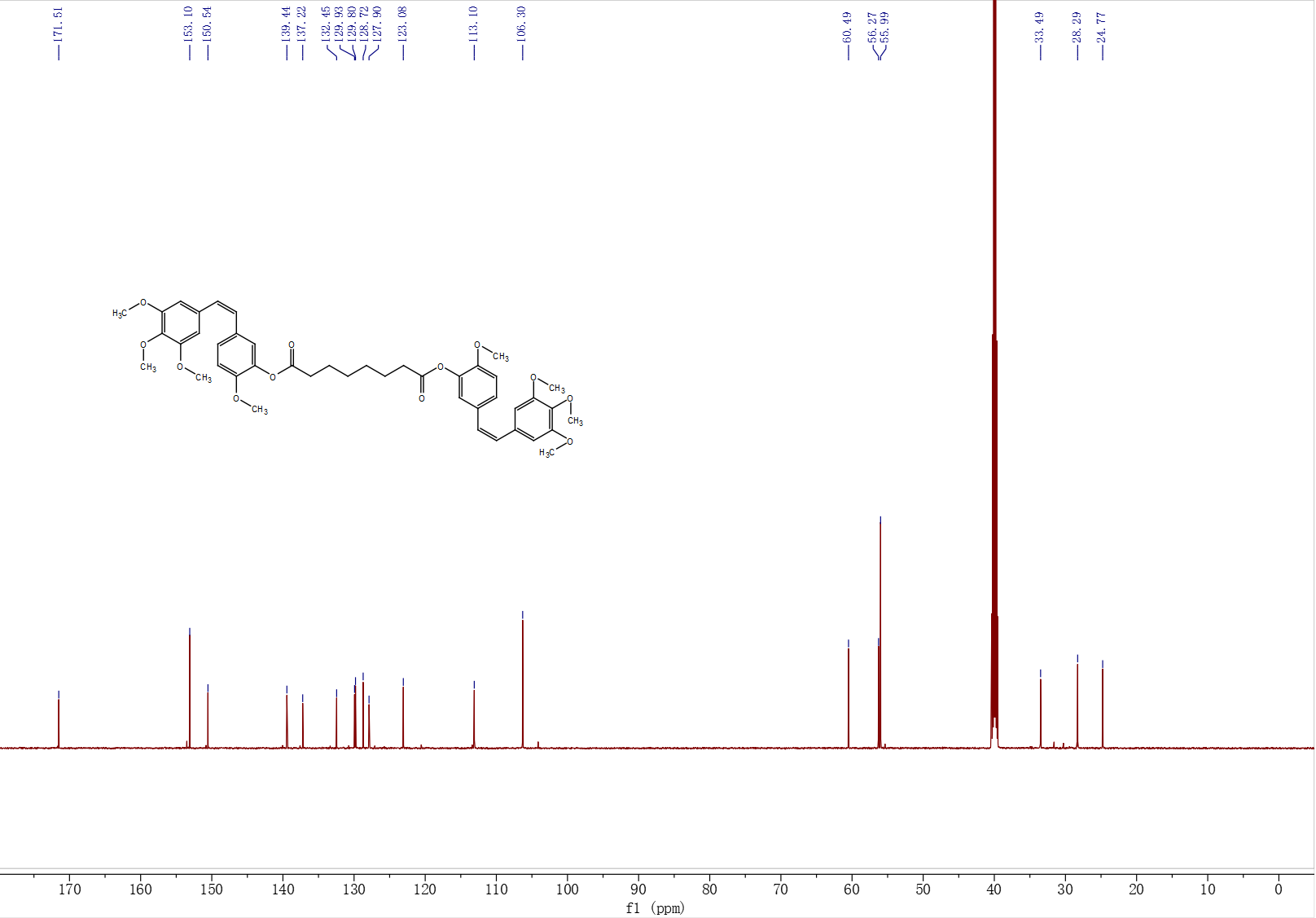
**

**Fig. S15 (A)** ^1^H-NMR and **(B**) ^13^C-NMR analysis of CA-4C_6_.

**A**

**
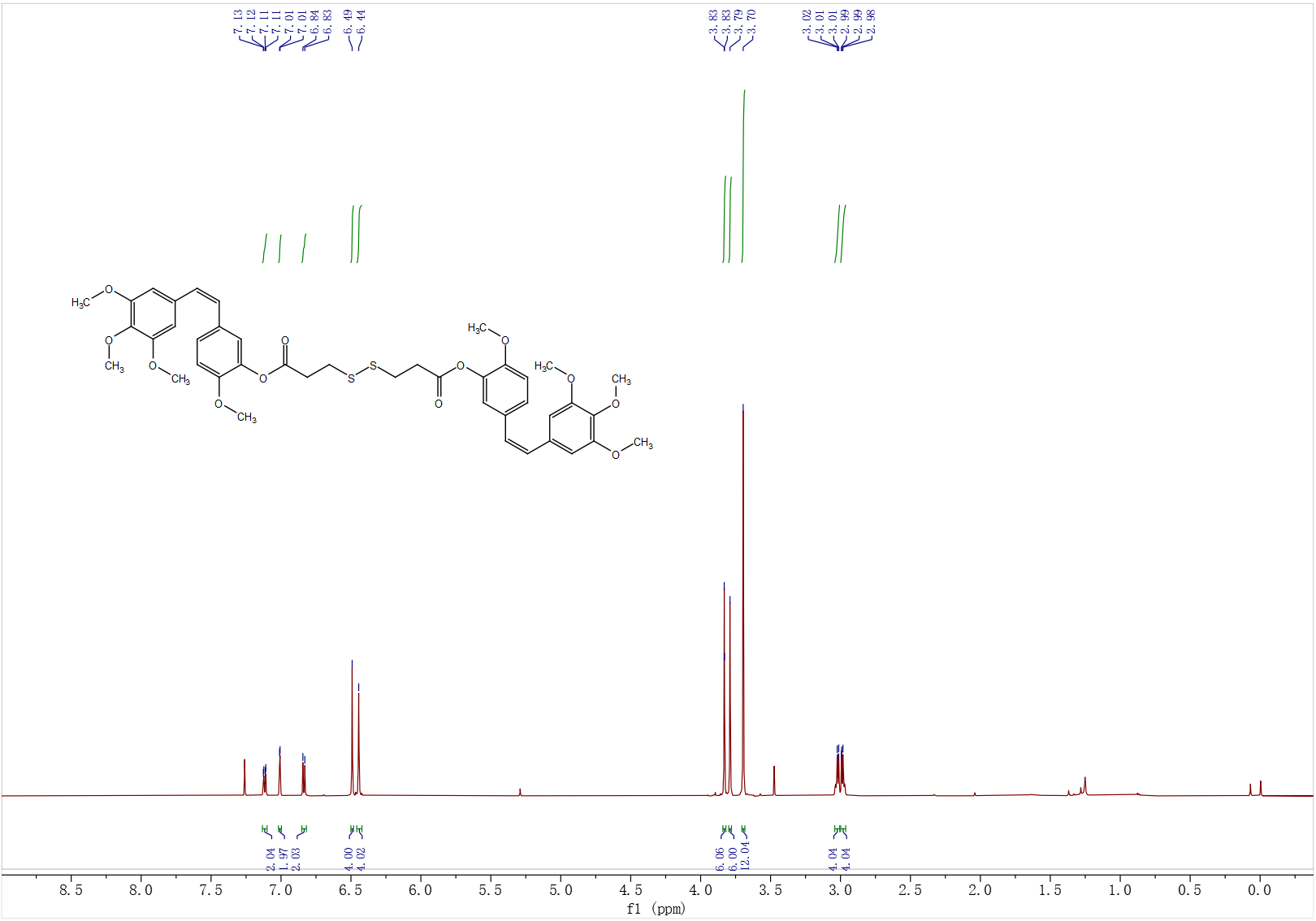
**

**B**

**
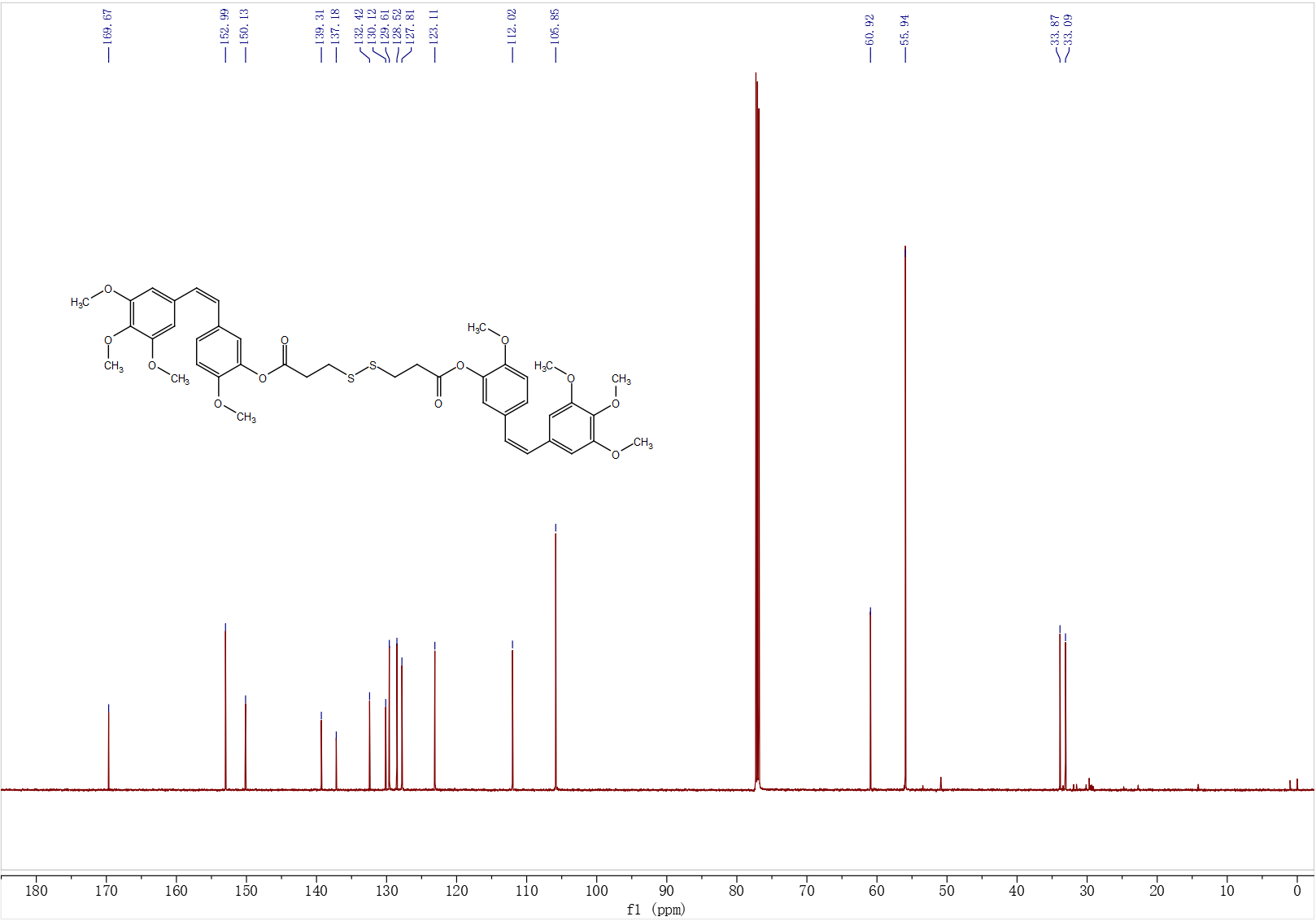
**

**Fig. S16 (A)** ^1^H-NMR and **(B)** ^13^C-NMR analysis of CA-4S_2_.

**Supplementary figures (S17-S31)**


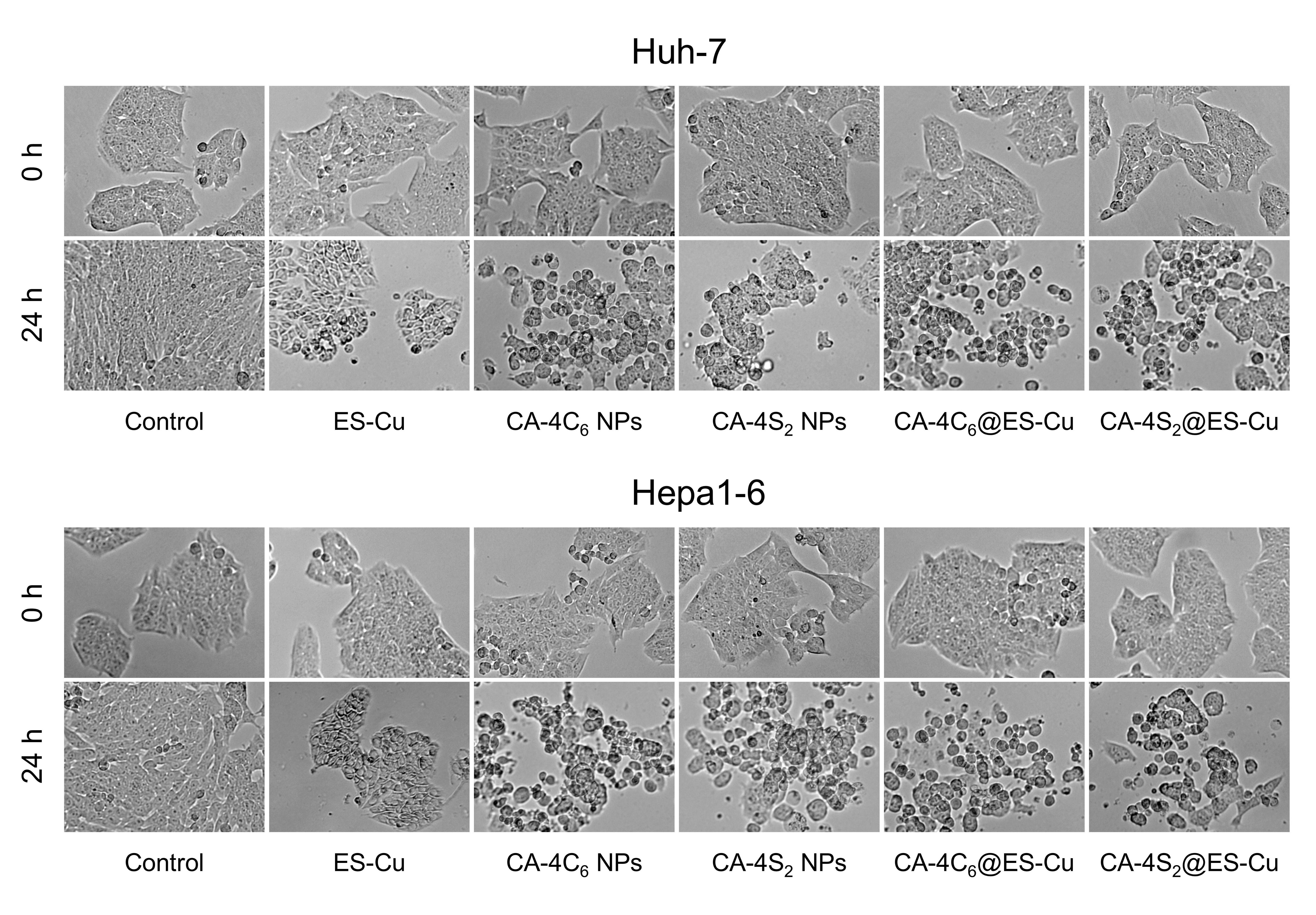


**Fig. S17** Morphologic changes in HCC cells after various drug treatments (×10).


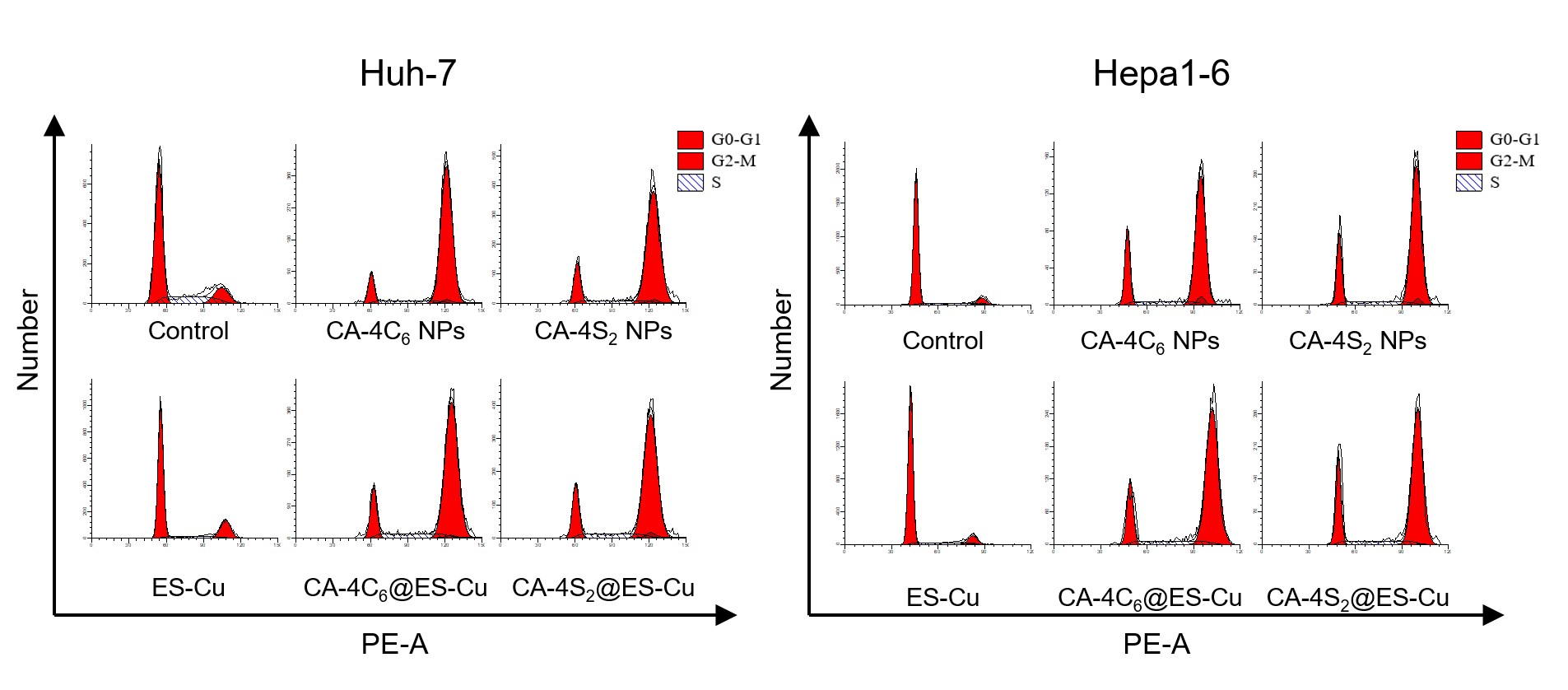


**Fig. S18** HCC cell cycle distribution after different drug treatments.


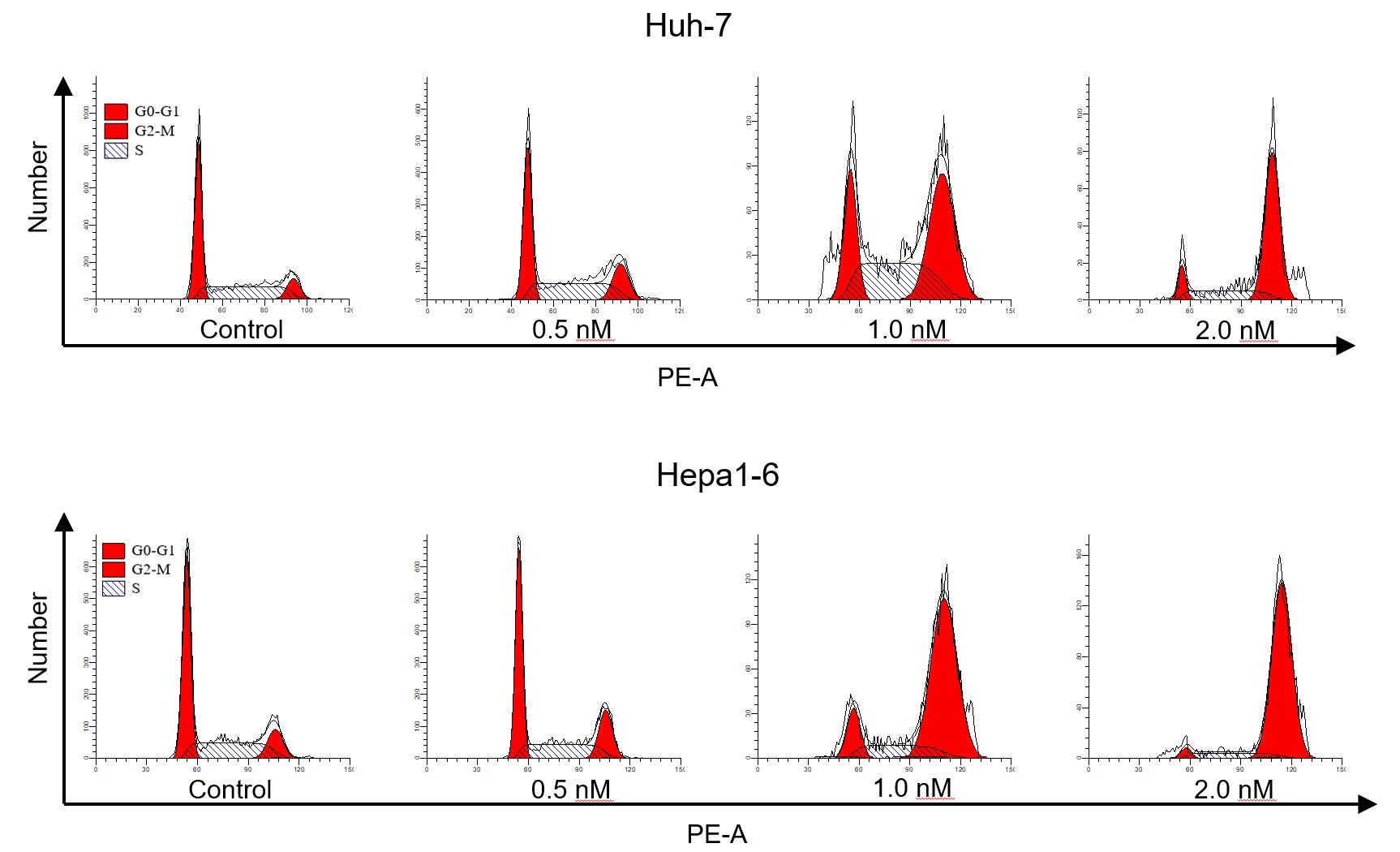


**Fig. S19** HCC cell cycle distribution treated with different concentrations of CA-4.


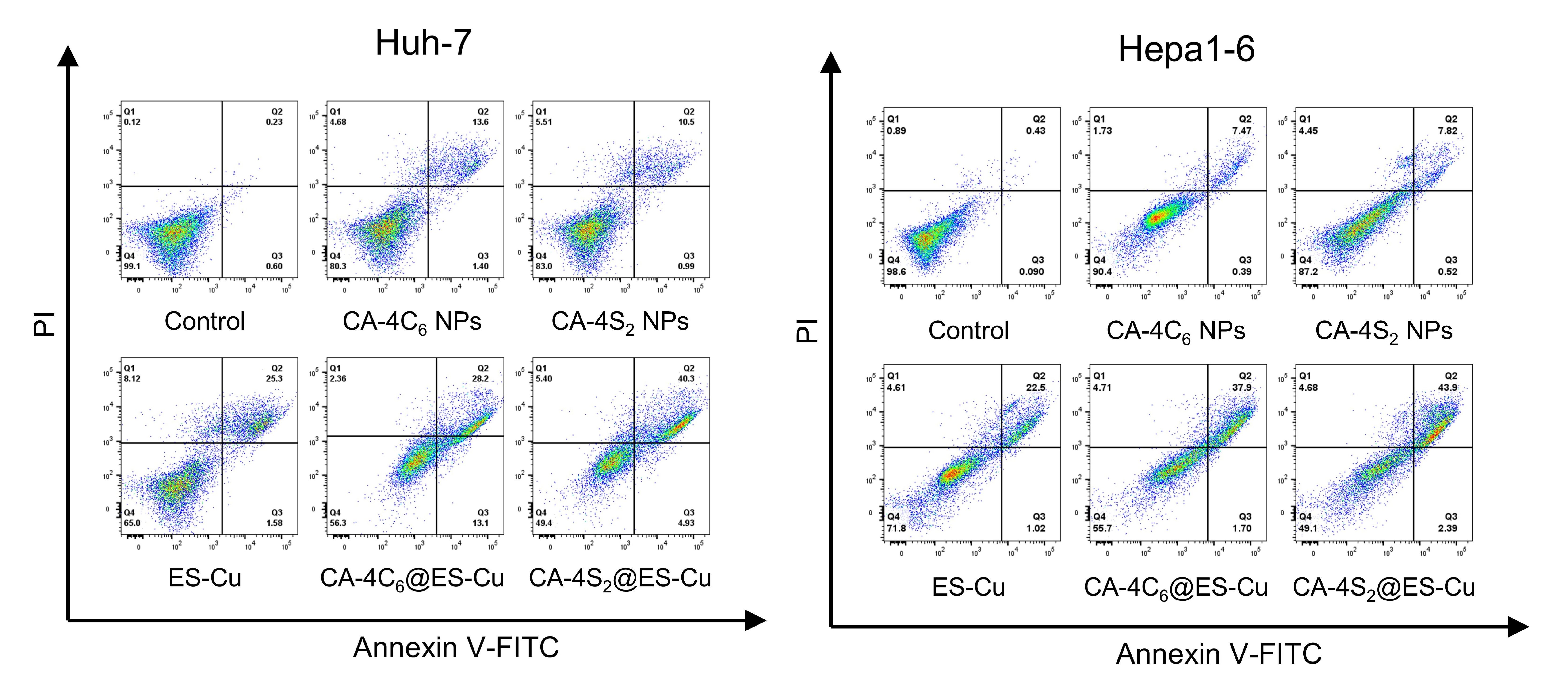


**Fig. S20** Apoptosis of HCC cells treated with different drugs analyzed by flow cytometry.


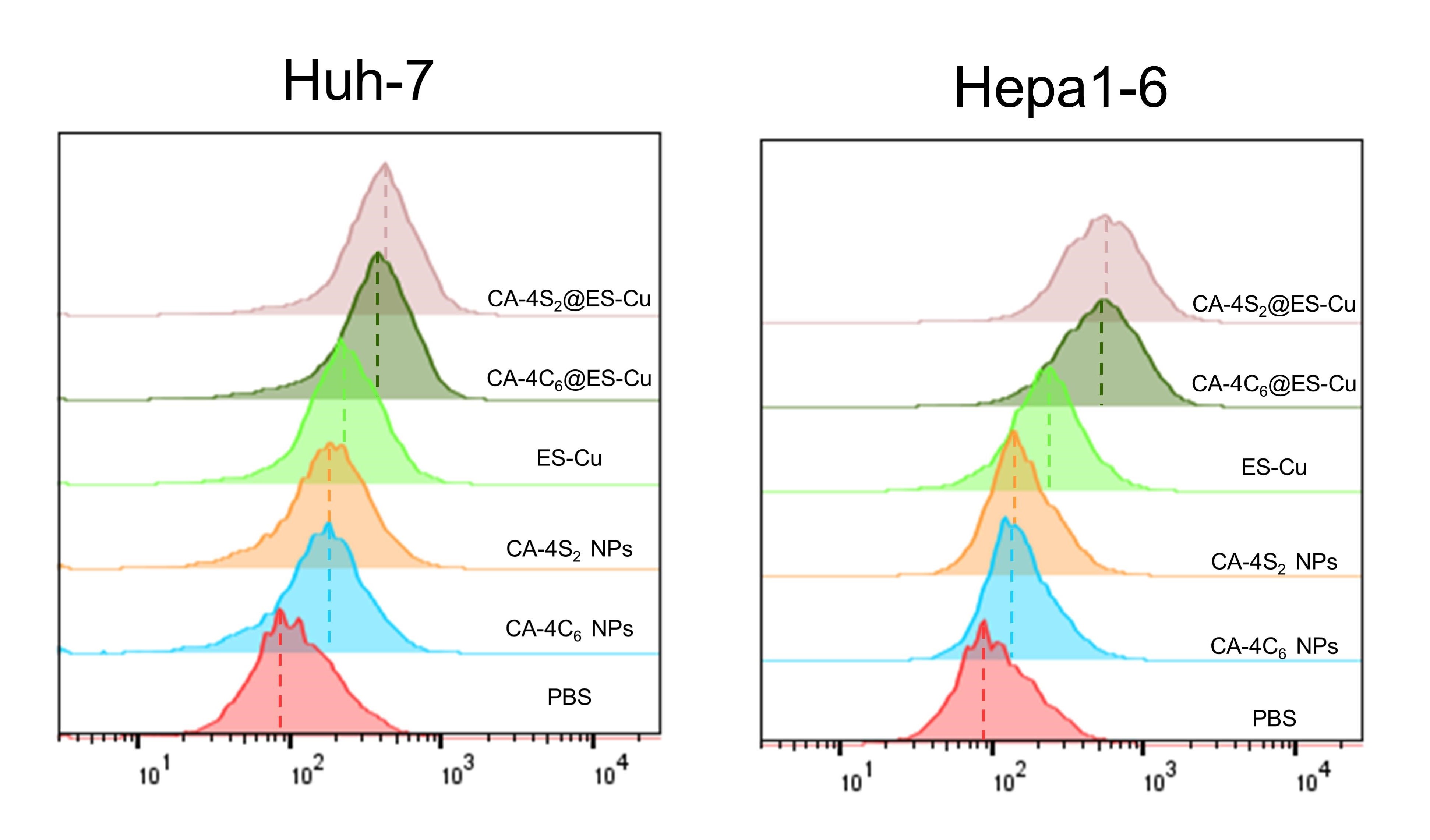


**Fig. S21** ROS levels in HCC cells after different treatments analyzed by flow cytometry.


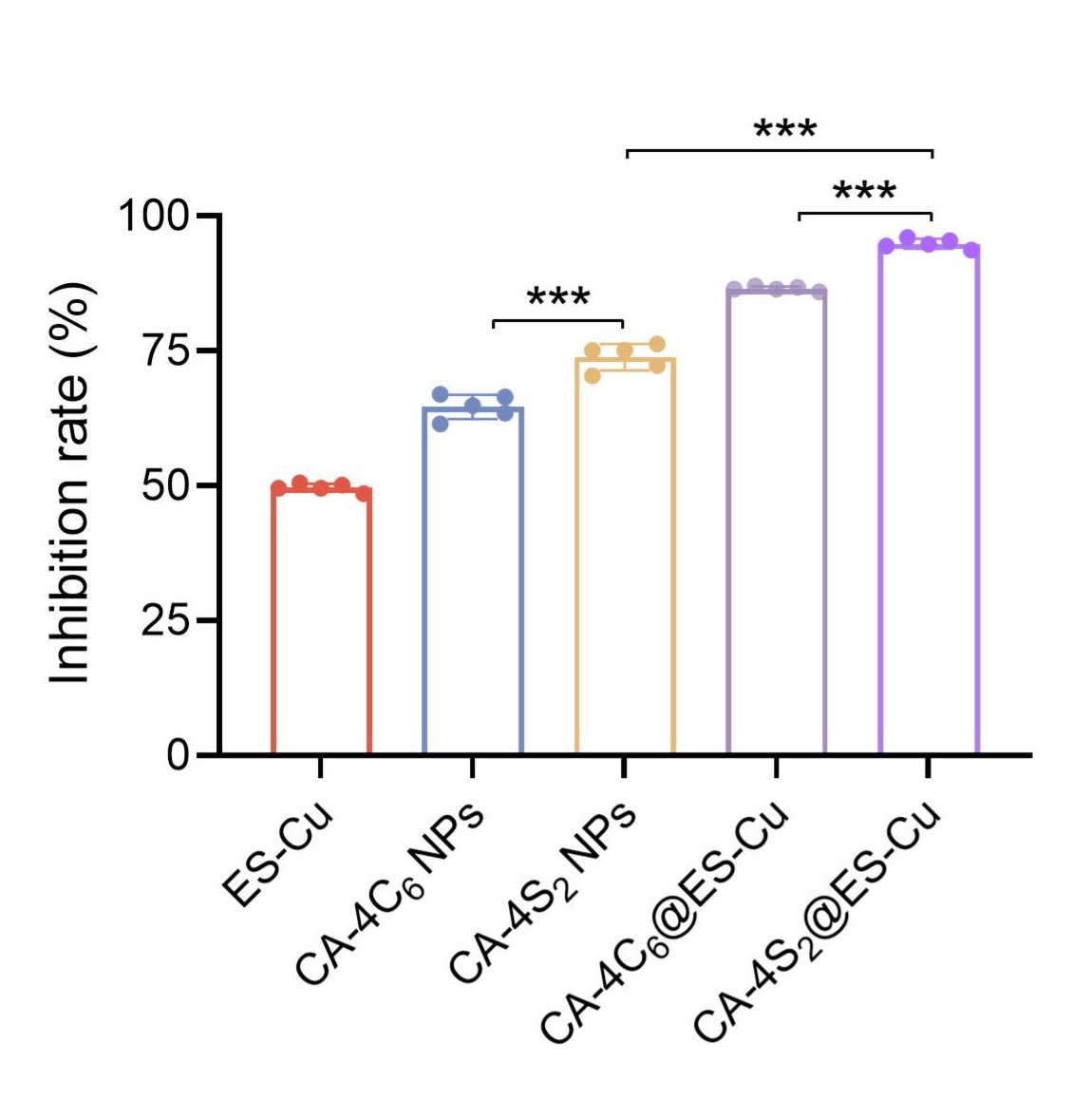


**Fig. S22** Tumor inhibition rates for different treatment groups.


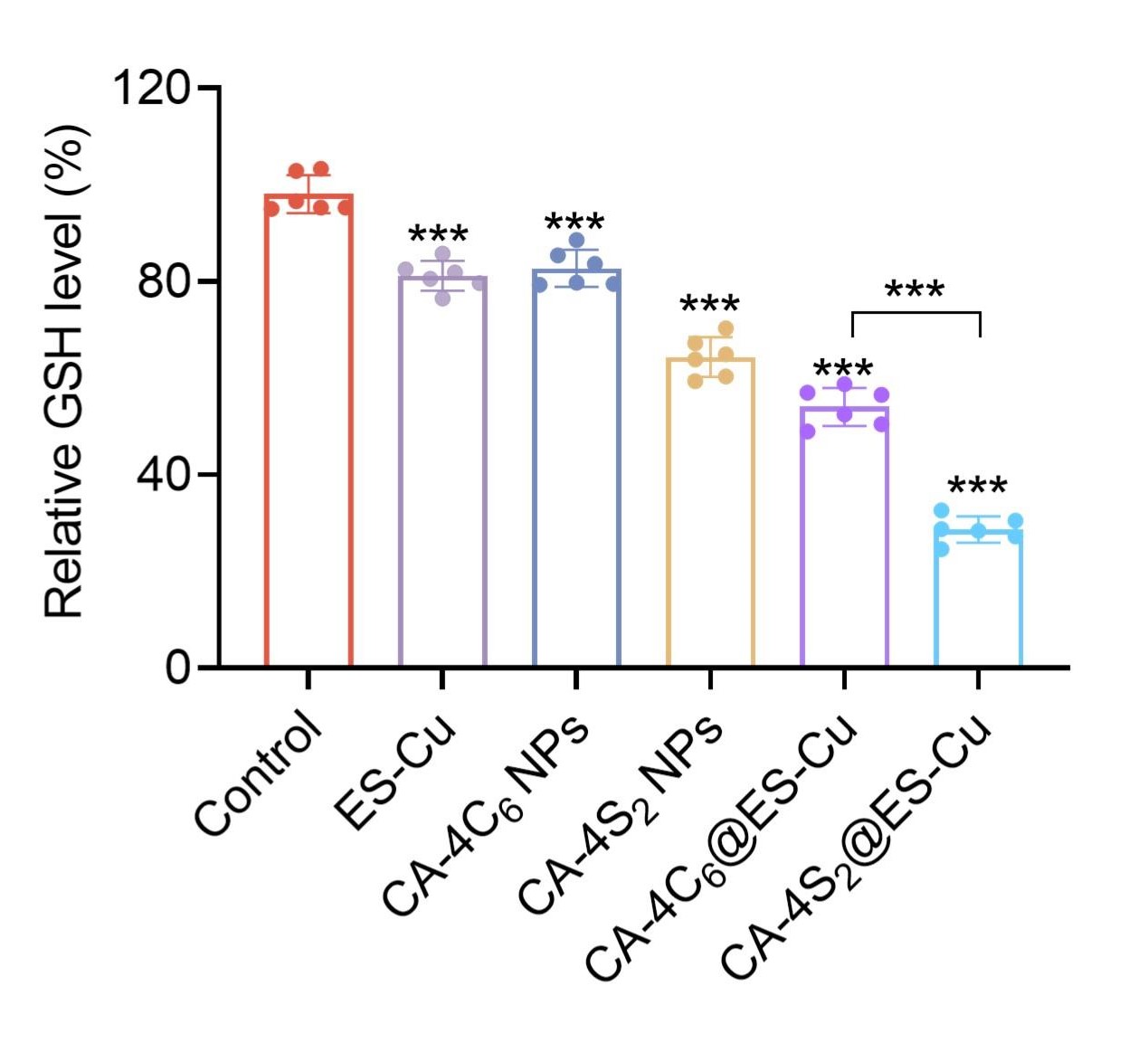


**Fig. S23** GSH levels in tumor tissues.


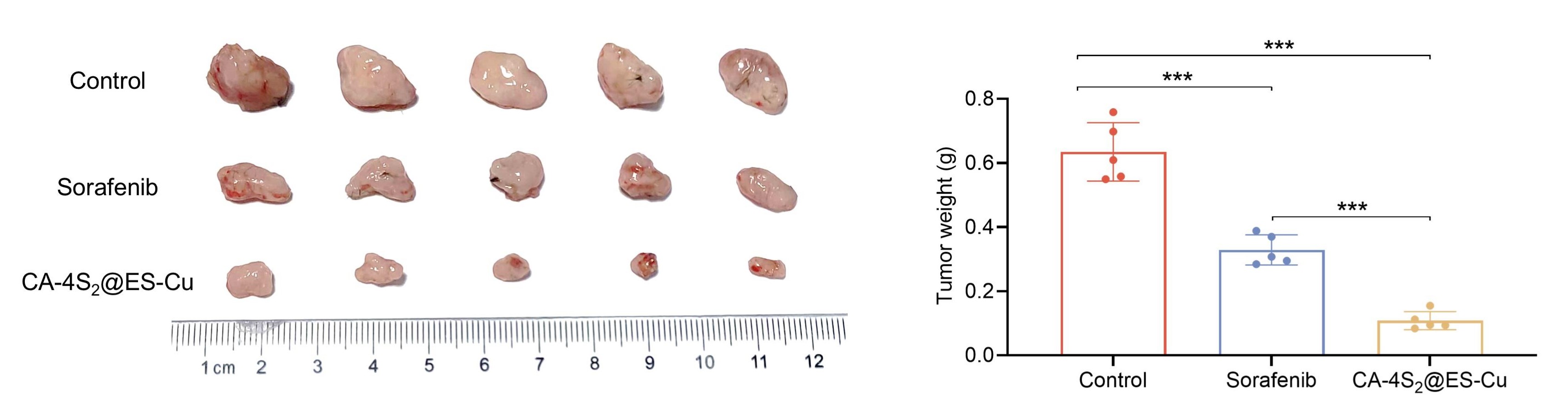


**Fig. S24** Representative images of tumor tissues and tumor weight from different treatment groups after 14 days of treatment.


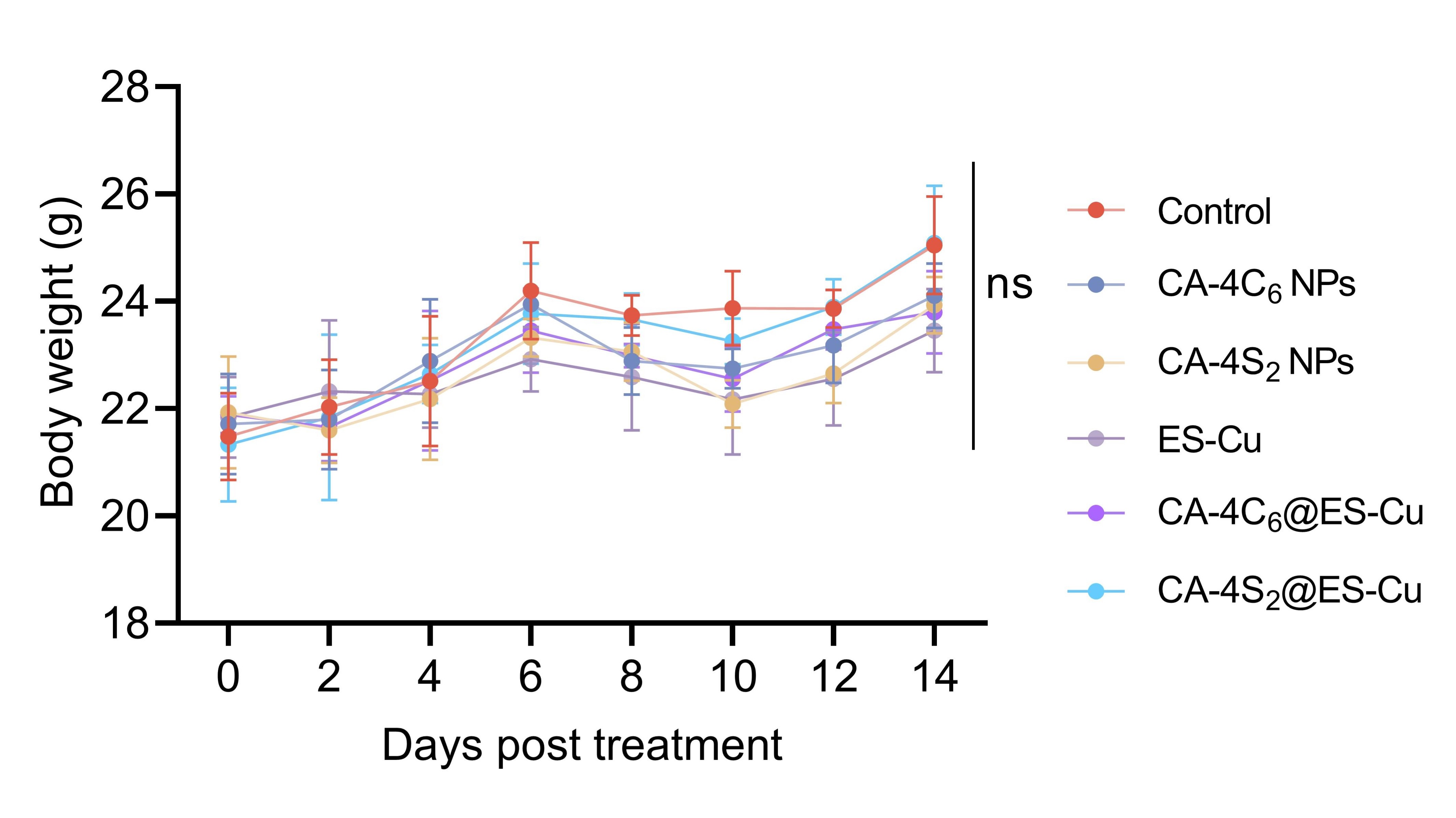


**Fig. S25** Body weight changes in each treatment group.


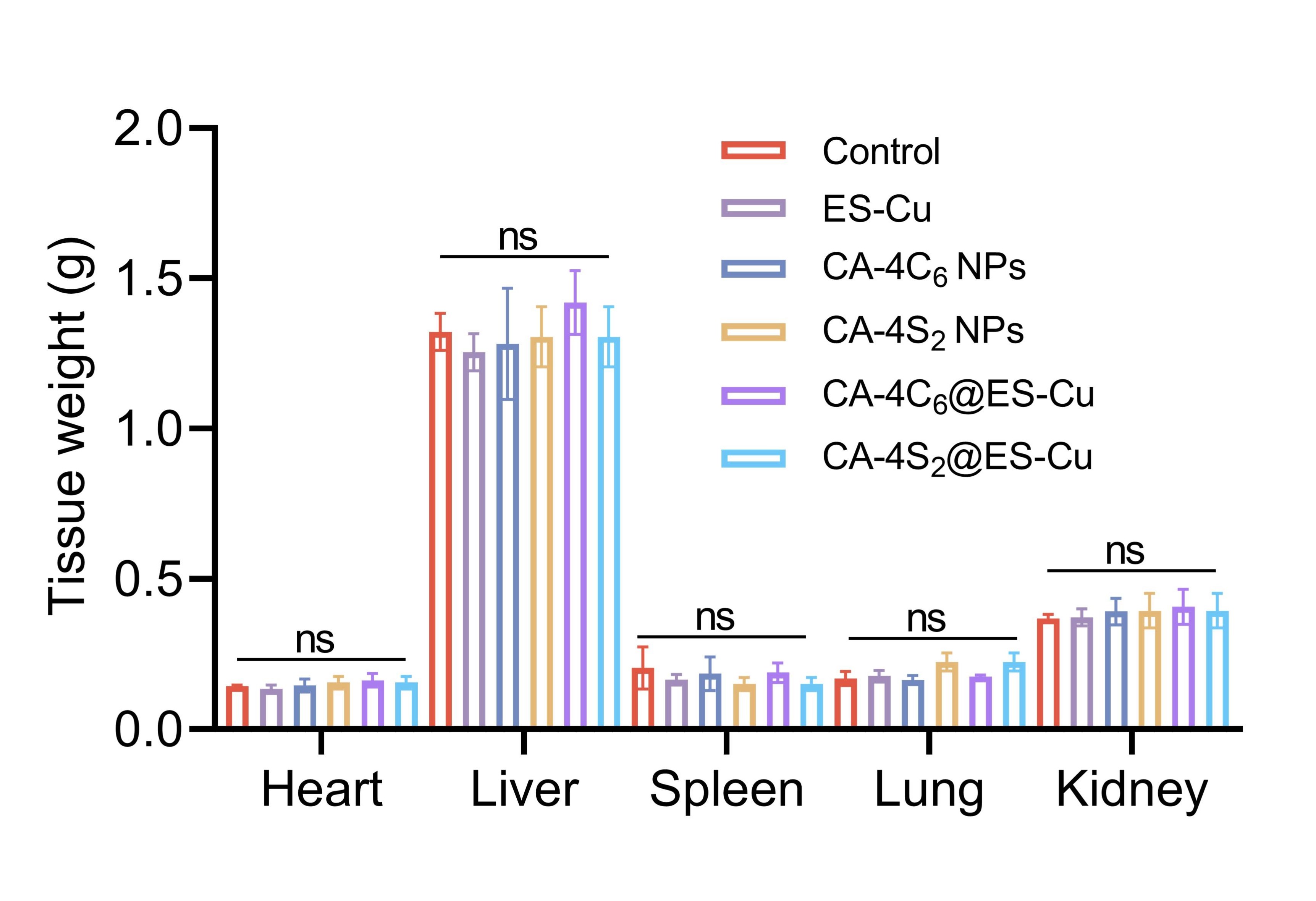


**Fig. S26** The tissue weight of the heart, liver, spleen, lung, and kidney after treatments.

**
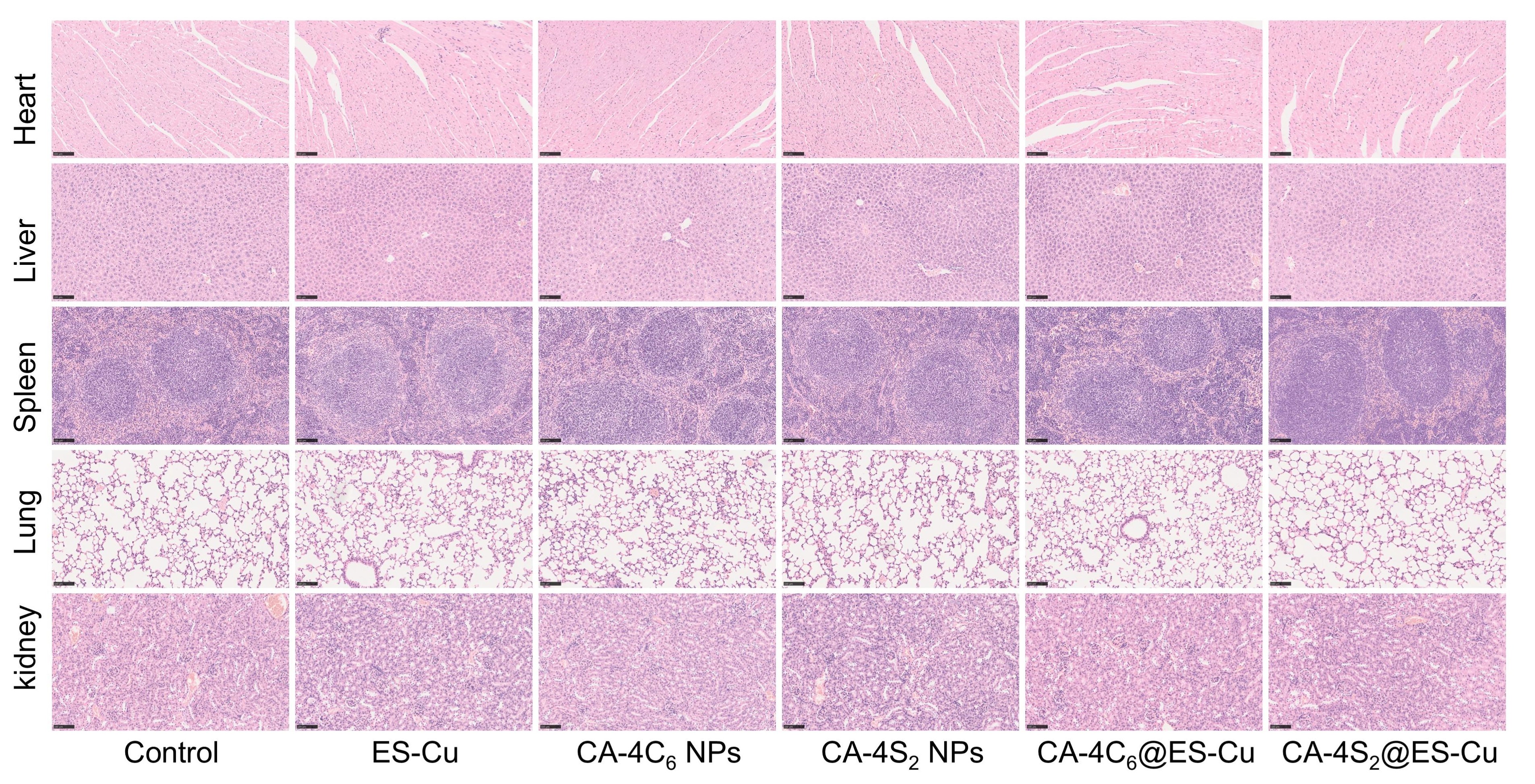
**

**Fig. S27** H&E staining images of major organs (heart, liver, spleen, lung, and kidney.


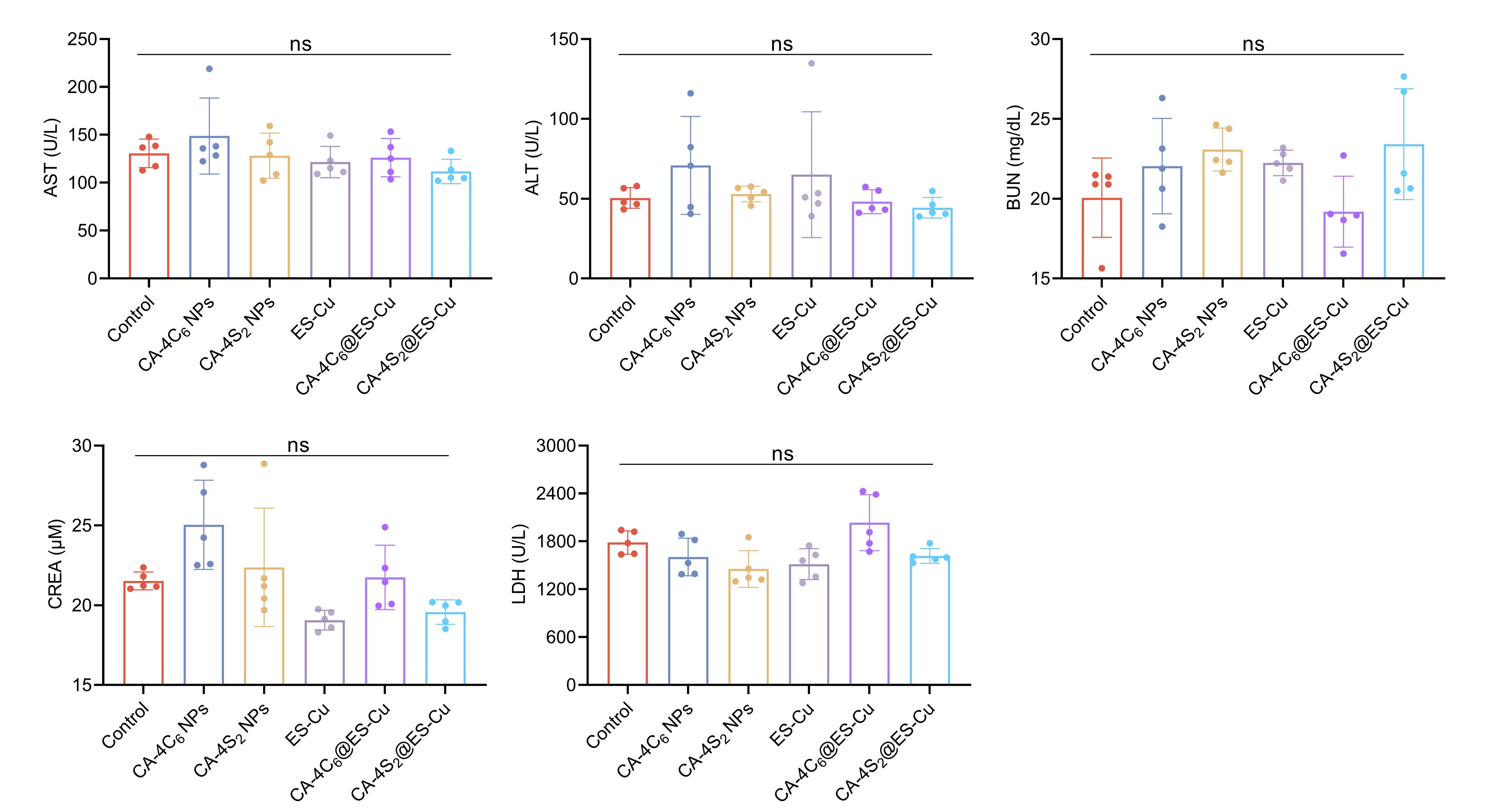


**Fig. S28** Biochemical analysis of serum from various treated mice.


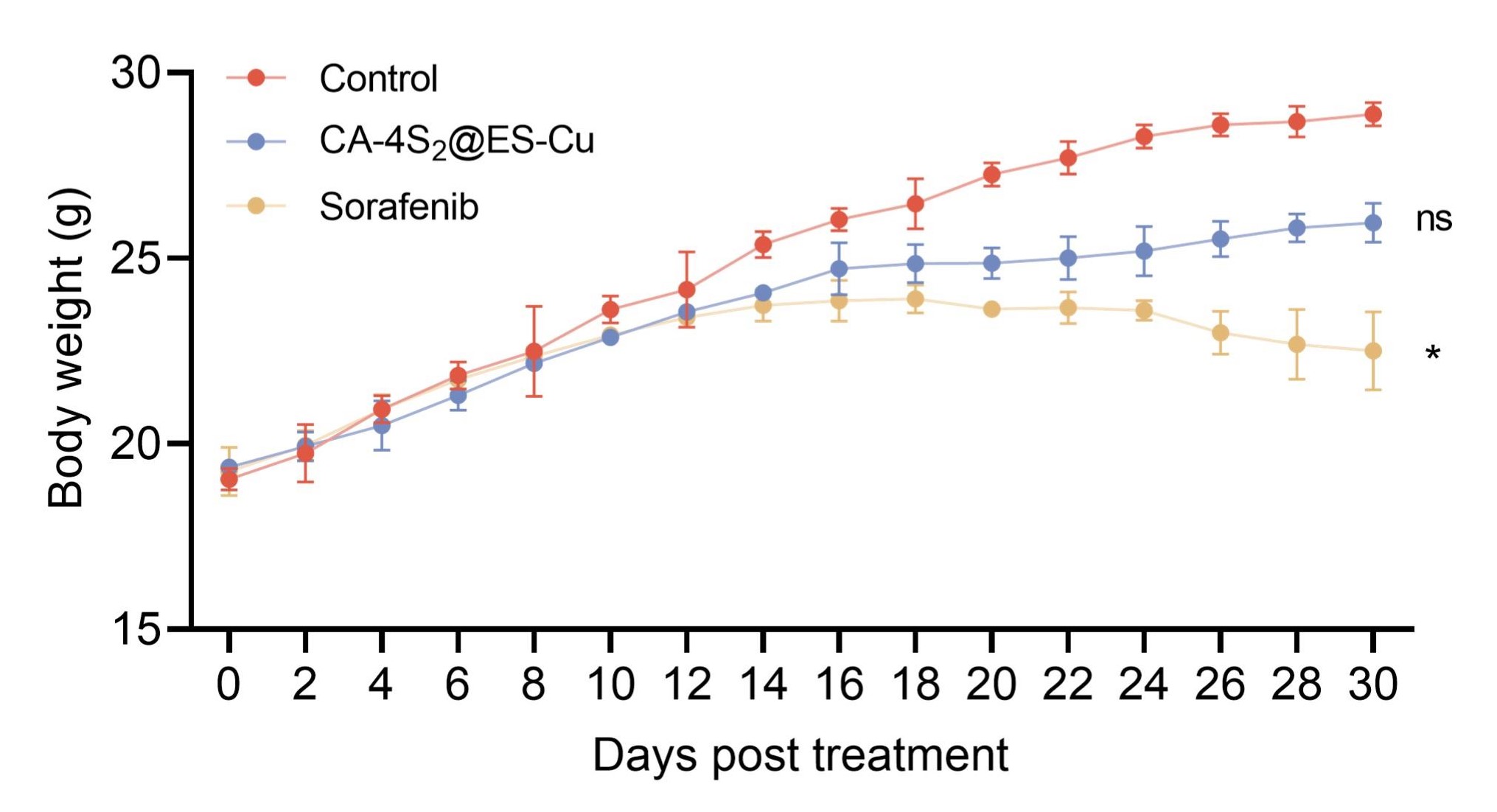


**Fig. S29** Body weight changes in each treatment group.


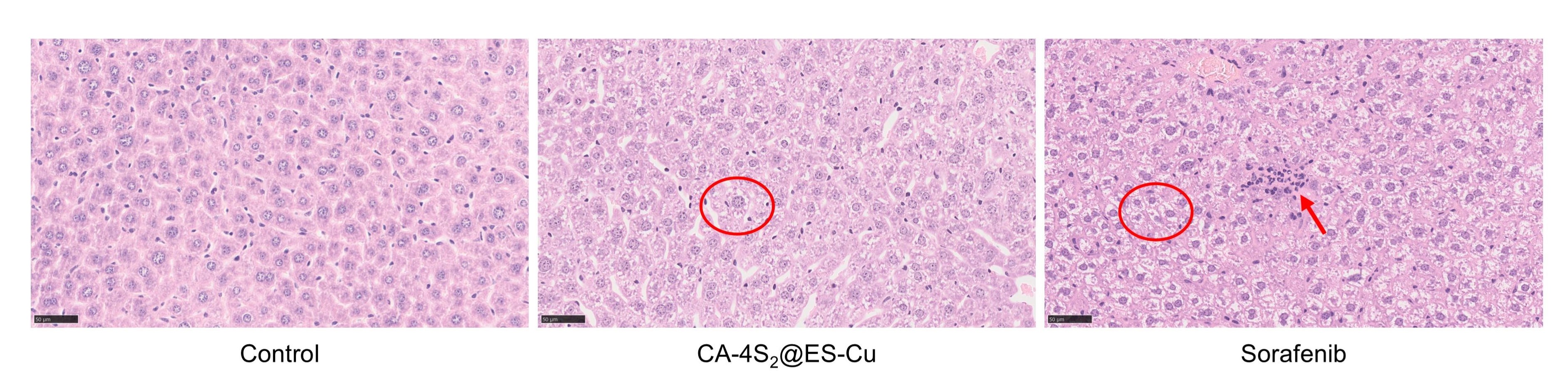


**Fig. S30** Representative H&E staining images of liver (40×). Circles indicated cytoplasmic laxity and arrows indicated inflammatory response areas.


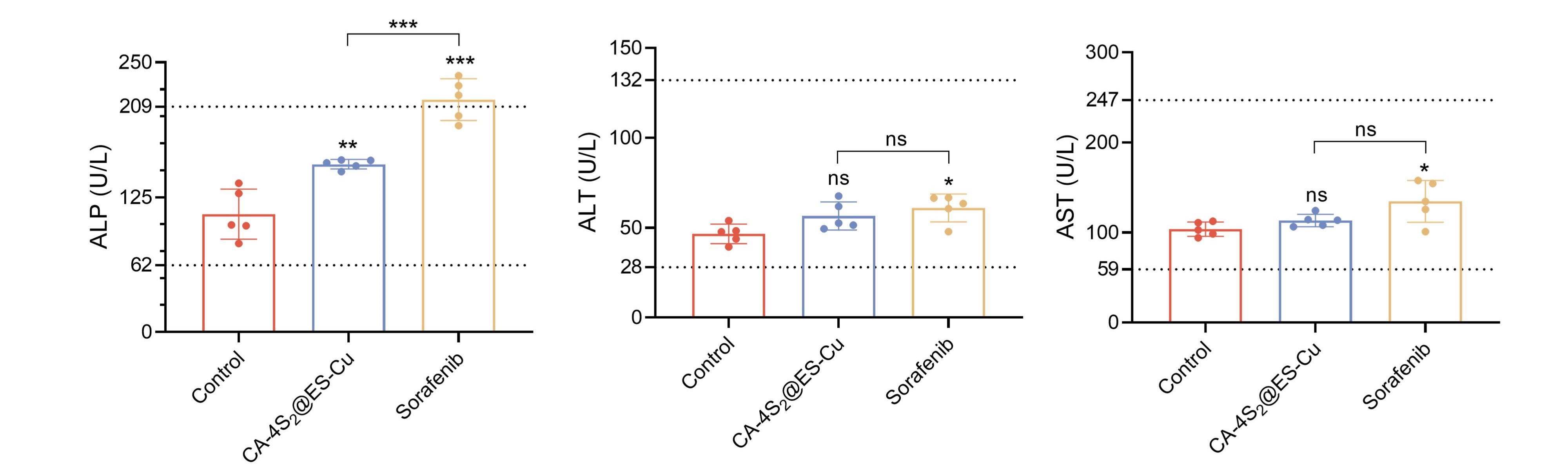


**Fig. S31** Biochemical analysis of serum from various treated mice.


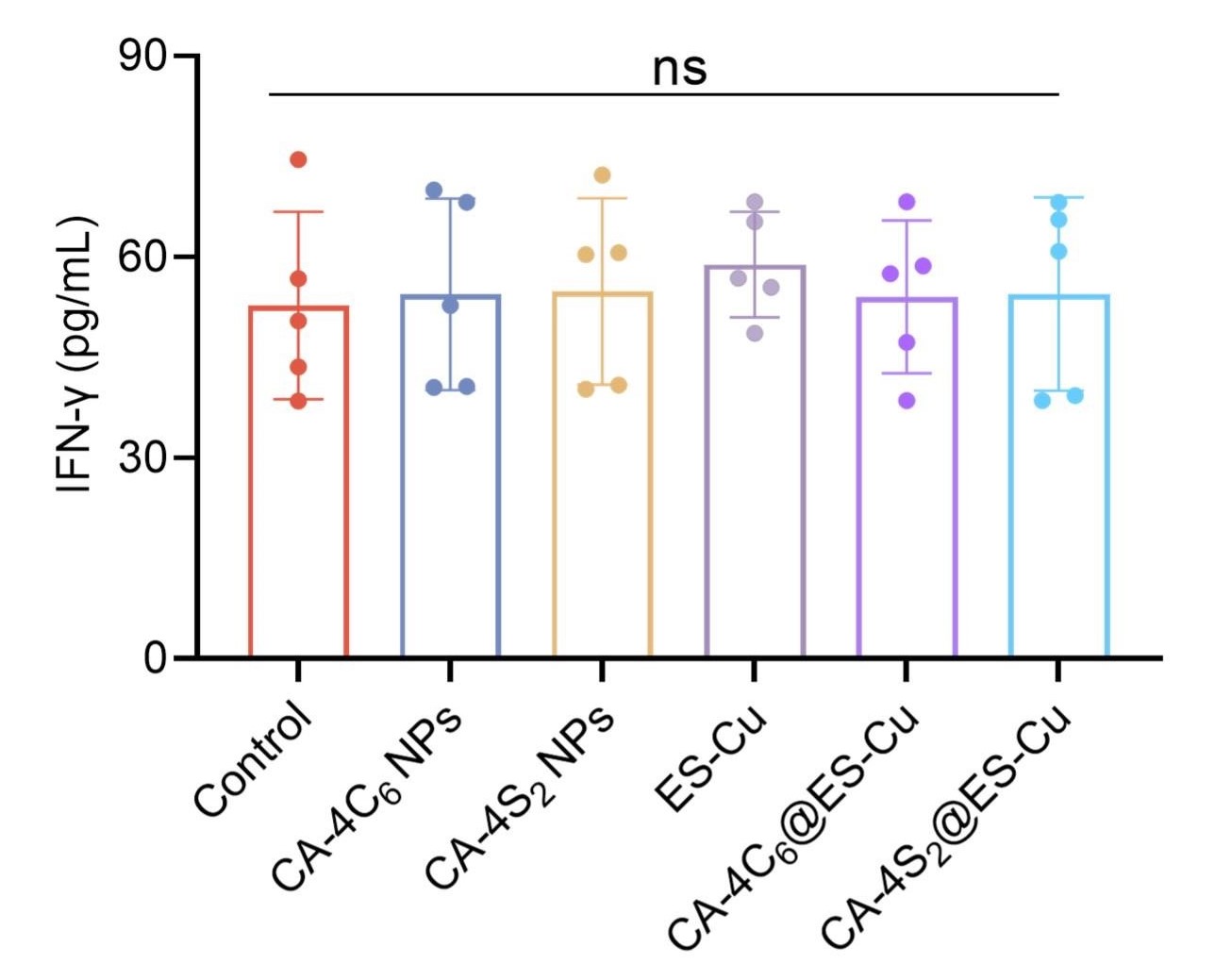


**Fig. S32** IFN-γ levels in mouse serum.

**Supplementary Table**

**Table S1** IC_50_ values for different cell lines treated with various drugs

| Drugs | IC_50_ (nM)^a^ | | | |
| --- | --- | --- | --- | --- |
|  | Huh-7 | Hepa1-6 | THLE-2 | AML-12 |
| CA-4 | 2.65±0.24 | 2.17±0.52 | 3.31±0.28 | 2.52±0.40 |
| CA-4C_6_ NPs | 3.19±0.09 | 2.90±0.12 | 3.34±0.14 | 3.40±0.34 |
| CA-4S_2_ NPs | 2.72±0.12 | 2.69±0.08 | 3.53±0.68 | 3.35±0.44 |
| ES-Cu | 12.84±0.52 | 14.64±0.14 | 15.51±0.54 | 16.73±0.45 |
| CA-4+ ES-Cu | 2.11±0.09 | 1.73±0.12 | 2.06±0.31 | 1.74±0.10 |
| CA-4C_6_ NPs+ ES-Cu | 2.89±0.04 | 1.51±0.03 | 5.31±0.79 | 1.94±0.37 |
| CA-4S_2_ NPs +ES-Cu | 2.39±0.08 | 1.96±0.02 | 3.78±0.15 | 2.21±0.22 |
| CA-4C_6_@ES-Cu | 8.46±0.59 | 9.33±0.16 | 23.8±0.32 | 26.21±1.46 |
| CA-4S_2_@ES-Cu | 10.96±0.39 | 9.45±0.19 | 31.62±0.66 | 28.76±1.00 |
| DSPE-PEG2000 | >1000 | >1000 | >1000 | >1000 |

*^a^* Data were the mean ± SEM of n ≥ 3.
